# Prediction of Protein–Ligand Binding Affinities Using Atomic Surface Site Interaction Points

**DOI:** 10.1021/acs.jcim.5c02628

**Published:** 2026-01-14

**Authors:** Katarzyna J. Zator, Maria Chiara Storer, Christopher A. Hunter

**Affiliations:** Yusuf Hamied Department of Chemistry, 2152 University of Cambridge, Lensfield Road, Cambridge CB21EW, U.K.

## Abstract

Atom surface site Interaction Points (AIP) which were previously used to predict association constants for synthetic host–guest systems has been extended to protein–ligand complexes. AIP descriptions of protein binding sites were obtained by combining a library of precomputed AIP descriptors for all protein functional groups with a graph-based substructure matching algorithm. The corresponding AIP description of ligands was obtained directly by footprinting the molecular electrostatic potential surface calculated using density functional theory. These AIP descriptions were projected onto X-ray crystal structures of protein–ligand complexes to identify pairs of AIPs that were sufficiently close in space to constitute an intermolecular interaction. The overall free energy of binding was calculated by summing the contributions of each AIP contact and associated desolvation. Application to the 94 complexes involving uncharged ligands in CASF benchmark data set showed that the method achieves a Pearson correlation coefficient of 0.76 and an RMSD of 11 kJ mol^–1^ for absolute free energies of binding.

## Graphical abstract

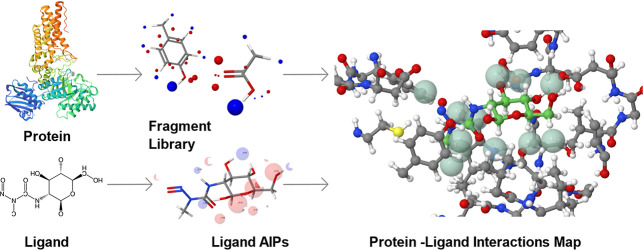

## Introduction

Computational methods for analysis of the protein–ligand interactions are an important aspect of modern drug discovery, and many different tools have been developed for prediction of binding affinity and screening large libraries of candidate small molecules.^1–10^ There is a clear trade-off between speed and accuracy.^11–16^ Free energy perturbation methods use molecular dynamics simulations, full atomistic solvation models and force-fields derived from ab initio calculations to make reasonably accurate predictions of the relative binding affinities of series of closely related compounds for a particular protein of interest.^17–22^ At the other end of the spectrum, scoring functions based on identification of close contacts between the atoms on the protein and the ligand in a candidate structure provide a rapid method for evaluating large numbers of different potential ligands and large numbers of different poses (different arrangements of the ligand in the binding pocket).^23–33^ These docking methods generally provide a ranking of compounds for the first round of in silico screening.^34,35^

We recently reported a new approach to predicting binding affinities for intermolecular complexes based on Atomic surface site Interaction Points (AIPs).^36^ The AIP approach is a computational tool that predicts an absolute value of the association constant for formation of an intermolecular complex but at relatively low computational cost. The method has been validated for a set of supramolecular host–guest complexes for which X-ray crystal structures and experimental association constants in a range of different solvents were available.^37^ Binding affinities in water and in organic solvents were accurately predicted to within 1 order of magnitude. Here we extend the AIP methodology to the analysis of protein–ligand complexes and show that starting from the X-ray structure of the complex, it is possible predict the binding affinity in water to within 2 orders of magnitude.

In the AIP approach, every atom in a molecule is described by a set of points that represent all of the noncovalent interactions that can be made with the surroundings. The location of the AIPs in space and the interaction parameters that describe their polarity, ε_i_, are both obtained from the molecular electrostatic potential surface (MEPS) calculated using density functional theory (B3LYP) in a process called footprinting (Figure 1).^36^ Each AIP represents approximately 9 Å^2^ on the van der Waals surface of the molecule, which is the surface footprint of a H-bonding interaction, and the value of ε_i_ is based on the electrostatic potential of the corresponding patch of the MEPS.^38,39^

**Figure 1:**
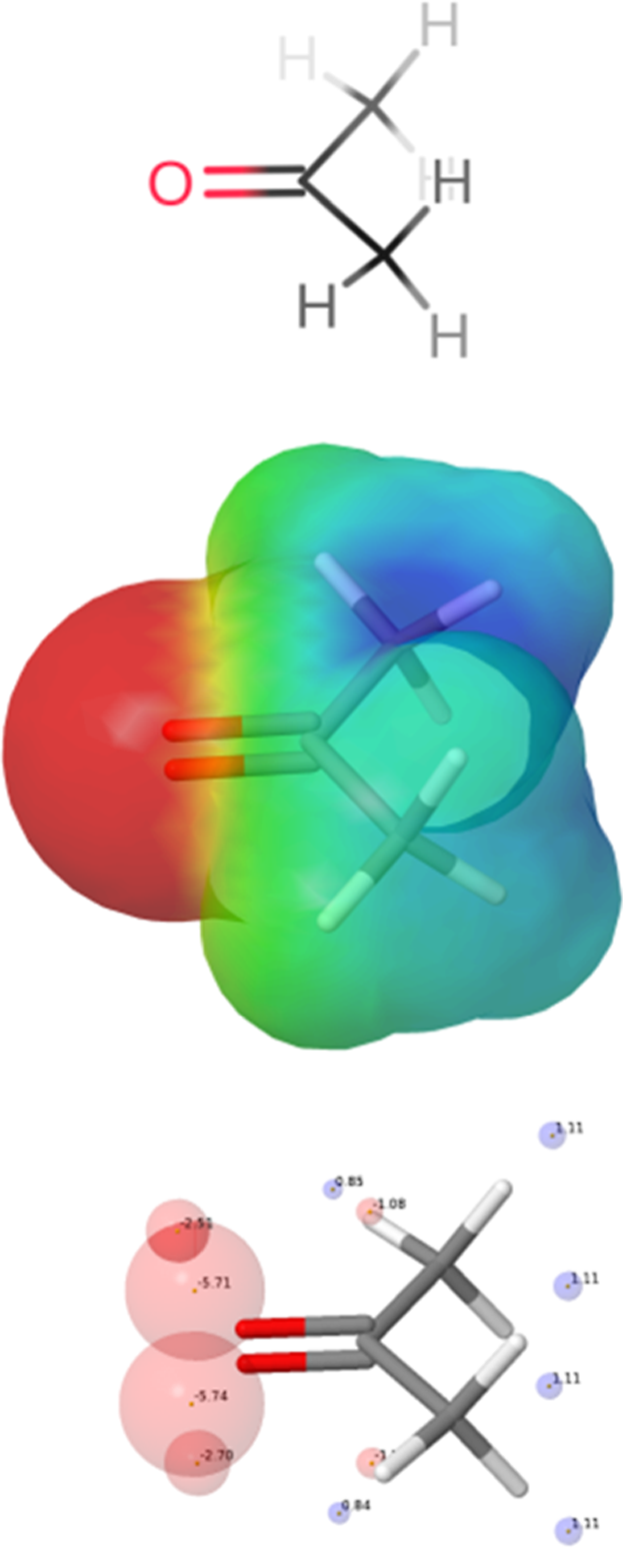
Footprinting process for the calculation of AIPs for acetone. The molecular structure is used to calculate the MEPS using DFT, and local values of the MEP are used to obtain AIPs that represent lone pairs (large red balls), π-sites (small red balls) and CH groups (blue balls).

When two molecules make a single point noncovalent interaction, the free energy change for the interaction is given by the product of the associated interaction parameters, i.e. ε_i_ε_j_.^39^ Thus, a liquid phase solution can be treated as a Boltzmann ensemble of pairwise interactions between AIPs, and AIP descriptions of solvents and solutes have been used in the SSIMPLE algorithm to calculate solvation energies in any solvent or solvent mixture.^39^ We have previously shown that AIPs can be used in SSIMPLE to make accurate predictions of phase transfer free energies between two solvents (e.g., water and *n*-hexadecane).^38,40^ For complexes that make multiple intermolecular interactions, the overall free energy change for formation of the complex is obtained by summing over all of the AIP contacts (Figure 2). Since the solvation energy of each AIP can be obtained from SSIMPLE, solution phase free energy changes can be obtained by including the desolvation energy, and this approach has been used to predict association constants for formation of host–guest complexes in organic solvents and in water.^37^ Here we develop this approach further by introducing a new methodology for obtaining AIP descriptions of macromolecules without the need for a full DFT calculation.

**Figure 2:**
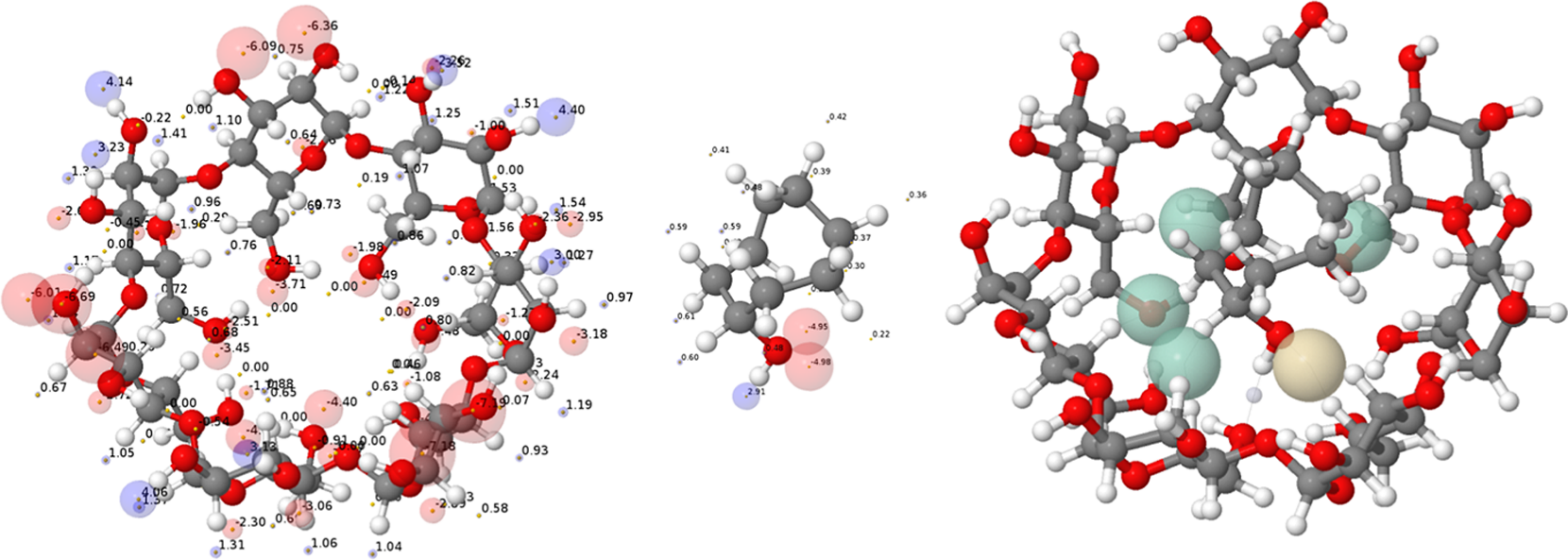
AIP analysis of the 1:1 complex formed by β-cyclodextrin and cyclooctanol in water. The AIP description of β-cyclodextrin and cyclooctanol are shown on the left: positive or H-bond donor sites are represented as blue balls, negative or H-bond acceptor sites are represented as red balls, and the size of the ball corresponds to the polarity of the site (i.e., the value of ε_i_). The AIP interaction map of the X-ray crystal structure of the complex shown on the right highlights the most important intermolecular interactions: green balls represent AIP contacts that contribute more than 0.5 kJ mol^–1^ to the stability of the complex, yellow balls represent AIP contacts that destabilize the complex by more than 0.5 kJ mol^–1^, and any contacts that make smaller free energy contributions are not shown.^37^

### AIP Description of Proteins

Calculation of the AIPs shown in Figures 1 and 2 is based on DFT calculation of MEP surfaces, which is not viable for macromolecules. However, if a macromolecule is composed of repeats of a small number of different building blocks, as in a protein, an AIP description of the macromolecule can be built up from AIP descriptions of the individual building blocks. Molecular fragments that represent each building block are first footprinted, and then a graph matching algorithm can be used to project the AIPs of each fragment onto the structure of the macromolecule. This approach requires that electronic communication between the fragments is minimal in the macromolecule, so that the AIPs are transferrable. Proteins are an ideal target from this point of view, because electronic communication between the functional groups present in each amino acid fragment is broken by the sp^3^ α-carbon atom.

Another important assumption in the fragment approach is that differences in the conformation of the fragment found in the macromolecule does not significantly perturb the functional group AIP values. We have found that through-space effects on AIP values are only important when two polar groups are very close in space. For example, an intramolecular H-bond that brings two AIPs into close proximity results in elimination of these AIPs from the description of the molecular surface. Thus, fragments were footprinted in an extended conformation, and we assume that these AIPs can be used for all amino acid conformations found in a protein.

In order to obtain AIP descriptions of the neutral functional groups present in amino acid side-chains, the α-carbon was replaced by a hydrogen, and the resulting compounds were footprinted in an extended conformation. Similarly, the AIP description of the protein backbone was obtained by footprinting *N*-methylacetamide. The results are illustrated in Figure 3 (see Supporting Information for details of the AIP interaction parameters). AIP descriptions of charged functional groups require a different treatment (see below), because the MEPS of a charged molecule calculated using DFT is dominated by the overall molecular charge.

**Figure 3:**
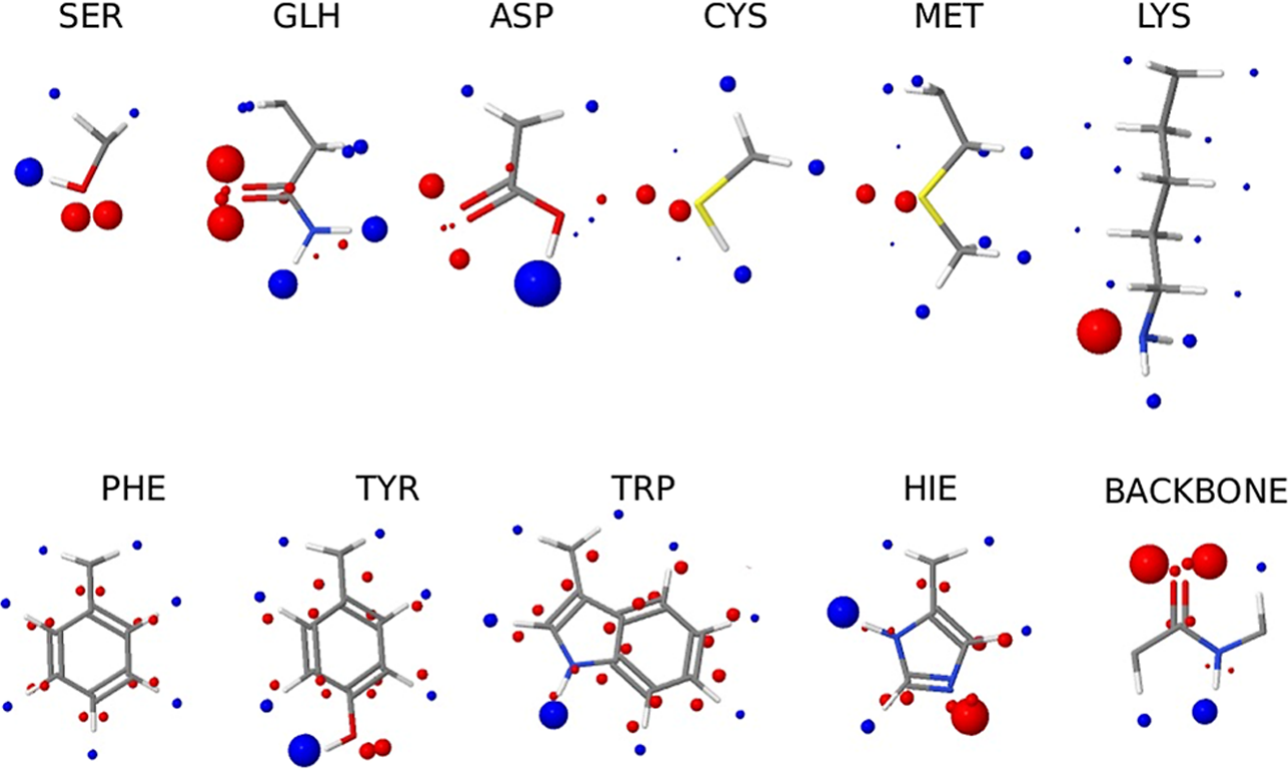
AIP description of functional groups present in neutral amino acid side chains and the backbone of a protein. Blue represents a positive AIP and red a negative AIP. The size of the ball is proportional to the corresponding interaction parameter, ε_i_.

In order to map the AIPs assigned to these fragments onto a protein structure a subgraph matching algorithm was employed.^42^ The protein and the fragments are each treated as a graph, i.e. a network of vertices that represent the atoms, connected by edges that represent the bonds. The subgraph matching algorithm recognizes when a graph in the fragment library matches exactly, by atom type and connectivity, to a subsection of the graph of the protein. Note that, from a chemical point of view, molecules are individual, complete entities and by definition cannot be fragments of a larger molecule. Therefore, for subgraph matching to work, at least one atom should be removed from the fragment molecules to allow for connection to other fragments in order to build up the larger molecule. However, a simpler approach is to alter the subgraph matching rules by allowing CH hydrogens in the fragment to match to sp^3^ carbon atoms that break electronic communication in the larger molecule. To increase the scope of the approach, the rule can be extended to all carbon atoms in the larger molecule bonded to a sp^3^ carbon. This modification of the subgraph matching algorithm allows us to treat small molecules as fragments without removing any atoms. Figure 4 illustrates the concept in more detail. Figure 4a shows a small molecule recognized as a subgraph of the larger molecule shown in Figure 4b. The arrows highlight the hydrogen atom of the small molecule matched with the carbon atom of the large molecule.

**Figure 4:**
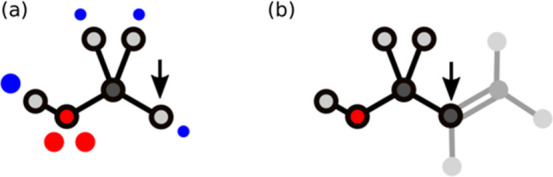
Subgraph matching with complete molecules. (a) Graph representation of the small molecule recognized as a fragment. (b) Graph representation of the larger molecule. The highlighted atoms indicate the subgraph match. The arrows indicate a hydrogen matched to a carbon atom.

The next step requires transferring the AIPs from the small molecule onto the larger molecule. This requires a description of the location of the AIPs that does not depend on the coordinates or orientation of the molecules. A solution has been implemented in the field of Molecular Dynamics.^43^ The method describes the location of virtual sites in terms of the location of atoms present in the molecule, rather than in an absolute frame of reference. eq 1 defines the position of an AIP, *r*_AIP_, relative to atom 1, the atom closest to the AIP, and atoms 2 and 3, the two atoms the shortest number of bonds away from atom 1 (Figure 5).

1 $$r_{AIP}=w_{12}r_{12}+w_{13}r_{13}+w_{cross}r_{12}\times r_{13}$$

where *r*_12_ represents the raw distance vector connecting atoms 1 and 2, *r*_13_ represents the raw distance vector connecting atoms 1 and 3, and the contribution of these vectors to the position of the AIP is defined by the weights *w*_12_, *w*_13,_ and *w*_cross_.

**Figure 5:**
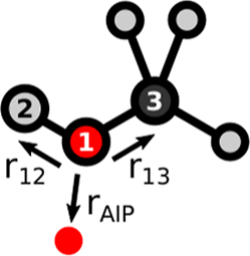
Position of the AIP shown in red is defined by the vector *r*_AIP_, which depends on the relative positions of the three atoms labeled 1, 2 and 3, defined by the vectors *r*_12_ and *r*_13_.

Figure 6 shows how the fragment-based approach works end-to-end, including the fragment library searching, the subgraph matching and the mapping of AIPs onto the molecule of interest. Figure 6a represents a larger molecule which will be described by employing the fragment-based approach. Figure 6b highlights the subgraph match between this molecule and a small molecule in a library. The set of small molecules identified as potential matches in the library is represented in the box in Figure 6b, and the molecule that was selected as the match was obtained by searching the library in such order that the fragment with the largest number of atoms is matched first. Figure 6c shows the AIPs added to the larger molecule by employing eq 1. AIPs are not added to atoms that already have AIPs or to carbon atoms that were matched to a CH hydrogen. Figure 6d shows the next match identified in the library. In this iteration, the compounds of the library that are considered are limited to the molecules that have the same number of atoms or fewer than the atoms of the larger molecule that are remaining without an AIP description. When a match is found, the AIPs are added as shown in Figure 6e. These steps are repeated until a complete description of the larger molecule is obtained.

**Figure 6:**
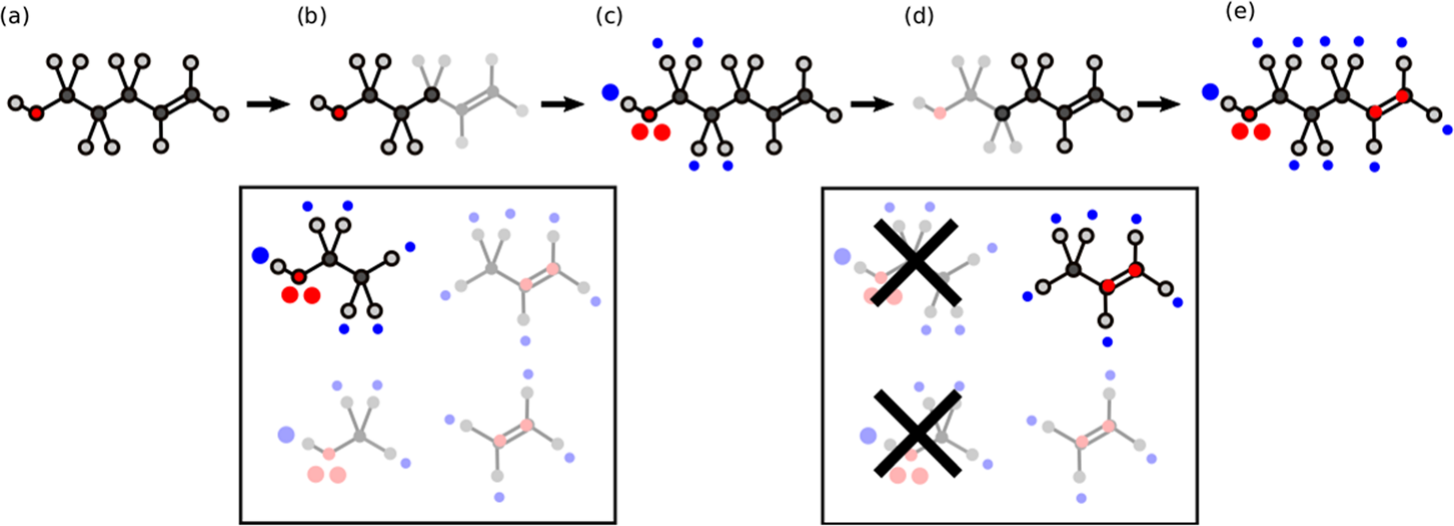
Fragment-based approach to calculation of AIPs. (a) The aim is to obtain an AIP description of a large molecule. (b) A library of small molecules (shown in the box) is screened to find a subgraph match, and the molecule with the largest number of atoms is selected as a match (highlighted). (c) AIPs of the matched small molecule are mapped onto the larger molecule. (d) The library of compounds is searched for a second match (highlighted). (e) Final AIP description of the large molecule.

This subgraph matching algorithm was also used to obtain AIP descriptions of the charged functional groups present in amino acid side chains and at the chain ends of the backbone. The approach is illustrated in Figure 7. The charged functional groups present in proteins are shown in Figure 7a, and neutral analogues that contain the same arrangement of atoms are shown in Figure 7c. The neutral analogues in Figure 7c were footprinted to obtain the locations of the AIPs required to describe the charged functional groups in Figure 7a. In order to obtain the interaction parameters for these AIPs, the values of the AIPs calculated for the neutral analogues in Figure 7b were used. The color-coding in Figure 7b indicates which atoms of the neutral molecules were employed to obtain the AIPs for the corresponding atoms in the charged functional groups. For the protonated amine, the experimental value of the H-bond donor parameter, α, for a neutral primary amine was used for the NH AIPs.^44^

**Figure 7:**
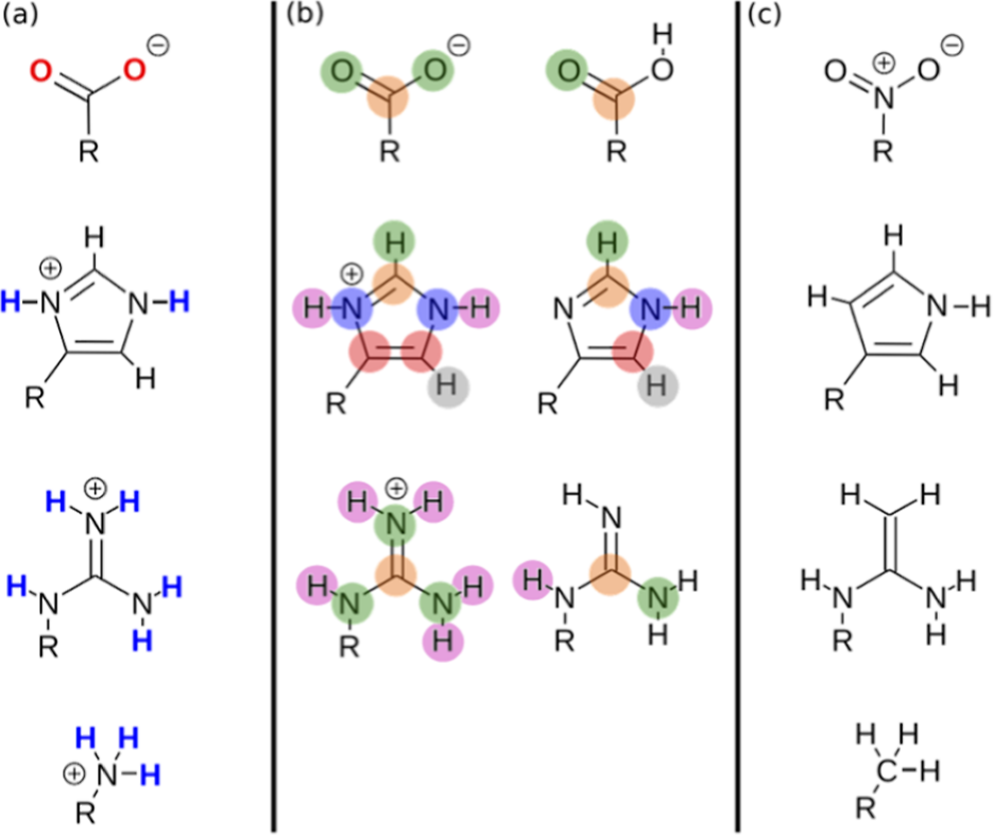
Calculation of AIPs for charged functional groups. (a) Charged functional groups present in proteins. (b) Atom mapping between the charged functional groups and the neutral analogues used to obtain the AIP interaction parameters: the color-coding indicates the atoms in the neutral molecule that were employed to obtain the AIPs for the charged group. (c) The neutral compounds that were footprinted to obtain the AIP locations.

The AIP code generates a description of a molecule in the CML file format.^45^ However, in the case of proteins, it is more manageable to employ the PDB file format, which contains additional information such as names of the amino acid residues.^46^ Moreover, there is a range of software designed specifically to work with the standard PDB labels which allows fast atom selection for visualization or to measure distances and other common applications.^35,43^ Fortunately, the OpenMM software already has a method in place for adding extra virtual sites onto PDB files.^46^ In order to exploit the OpenMM functionality, an AIP description was written in the OpenMM format for force-fields, and this information was employed to add AIPs as virtual sites onto the PDB file. Using this approach, the fragment library containing the AIP descriptions of the amino acid side chains and backbone can be used to obtain the overall AIP representation of a protein in the PDB format. The full OpenMM force-field description of protein AIPs can be found in the Supporting Information.

### Prediction of Association Constants Based on AIPs

The approach used to convert an AIP description of the three-dimensional structure of a protein–ligand complex into an association constant (or free energy change for the binding interaction) is based on the method that we published previously for host–guest complexes. First the ligand was removed from the structure of the complex, the ligand MEPS was calculated using DFT, and footprinting of this surface gave the AIP description of the ligand. This AIP description was then projected onto the three-dimensional structure of the complex, and the AIP description of the protein was added using the OpenMM force-field. The AIP pairing algorithm described previously was then used to identify ligand and protein AIPs that make interactions in the complex, in a stepwise analysis.^43^ First, the solvent accessible surface area (SASA) associated with each AIP was calculated using a probe radius of 0.35 Å, and any AIP with a SASA greater than 9.8 Å^2^ was assumed to interact with solvent and eliminated from further consideration. Then the distances between all H-bond donor and acceptor heavy atoms were calculated, and H-bonding interactions between the ligand and protein were identified based on the distance between the heavy atoms (<3.0 Å) and the distances between the associated AIPs (<2.2 Å). For the remaining AIPs, a list of potential protein–ligand contacts was created based on all pairs of AIPs that were less than 1.7 Å apart. An adapted maximum bipartite pairing algorithm was then used to identify the set of contacts that maximized the number of AIP pairings and minimized the sum of the distances between paired AIPs.

For each contact, the free energy associated with the interaction of AIP *i* with AIP *j* is given by the difference between the energies of the AIPs in the bound state, Δ*G*_B_, and the solvation energies in the free state, Δ*G*_S_, as described previously (eq 2).^41^

2 $$\Delta \Delta G\left(i,j\right)=\Delta G_{B}\left(i\right)+\Delta G_{B}\left(j\right)-\Delta G_{S}\left(i\right)-\Delta G_{S}\left(j\right)$$

The free energy of each AIP in the bound state is given by eq 3.

3 $$\Delta G_{B}\left(i\right)=\Delta G_{B}\left(j\right)=RT\ln\left(\frac{\sqrt{1+8\theta }-1}{4\theta }\right)+RT\ln\frac{\sqrt{1+4\theta \left(K_{ij\;}+K_{vdW}\right)\;}-1}{2\theta \left(K_{ij\;}+K_{vdW}\right)}$$

where θ is the total AIP density of the solvent, and *K*_vdW_ and *K*_ij_ are the association constants for polar and nonpolar contacts defined in eqs 4 and 5.

4 $$K_{vdW}=\frac{1}{2}e^{-E_{vdW}/RT}$$

where *E*_vdW_ is −5.6 kJ mol^–1^.^38,39^

5 $$K_{ij}=\frac{1}{2}e^{-\left(\epsilon_{i}\epsilon_{j}+E_{vdW}\right)/RT}$$

except when ε_i_ε_j_ is positive, in which case *K*_ij_ is set equal to *K*_vdW_, because the SSIMPLE formulation assumes that repulsive interactions can be avoided by reorientation of dipoles.^41^

The solvation free energy of each AIP in the free state can be calculated using SSIMPLE.^39^ In SSIMPLE, all pairwise interactions between solvent and solute AIPs in the liquid phase are described by eq 5, which allows calculation of the Boltzmann distribution of AIP contacts and hence the solvation energy for an individual solute AIP, Δ*G*_S_(*i*). We have shown previously that this approach provides a quantitatively accurate value for the solvation energies of both polar and nonpolar functional groups in water.^36^

The overall free energy change for formation of the protein–ligand complex is simply the sum of the individual contributions from each AIP contact scaled by the fractional parameter, *f*, for interactions involving fractional AIPs (eq 6).^36^ Thus, eqs 2–6 allow conversion of a list of AIP contacts into the association constant (*K*) or free energy change (Δ*G*°_calc_) for formation of the protein–ligand complex.

6 $${-RT\ln K=\Delta G^{{}^{\circ}}}_{calc}=\sum_{i,j}f\Delta \Delta G\left(i,j\right)$$

### Analysis of Protein–Ligand Complexes

The set of protein–ligands complexes used previously for comparative assessment of scoring functions (CASF) was used to test the approach described above.^47^ For these complexes, both the high resolution X-ray crystal structure and the experimentally determined affinities (dissociation constant *K*_d_, or inhibition constant *K*_i_) are available, providing an ideal benchmark for the quantitative predictive power of eq 6. The CASF data set contains 285 protein–ligand complexes, the core set, where no ligand occurs more than once, the binding affinities span a wide range, the qualities of the structures are good (resolution <2.5 Å, *R*-factor <0.25), and there are no key amino acid residues that are missing or conformationally ill-defined in the binding site. However, the AIP approach cannot handle charged ligands or metal ions, so complexes that had these features were removed, resulting in a total of 94 protein–ligand complexes (see Supporting Information for PDB identifiers).

The authors of the CASF study used the SYBYL software to add protons to the structures obtained from the PDB and assumed neutral pH to assign protonation states, i.e. all glutamates and aspartates were carboxylate anions, and all lysines and arginines were protonated cations. However, there were some errors in the placement of polar hydrogens, which were corrected manually: Figure 8a shows an example of an OH hydrogen that should make a H-bond with the neighboring backbone amide oxygen atom but points toward the π-face of an aromatic ring in the ligand; Figure 8b shows an example of two polar hydrogens that point toward each other. Visual inspection of any complexes that produced unusually repulsive AIP contacts proved to be an effective method for identifying obvious errors in protonation states or the placement of polar hydrogen atoms. For these structures, the position of the hydrogen or protonation state of the residue was corrected.

**Figure 8:**
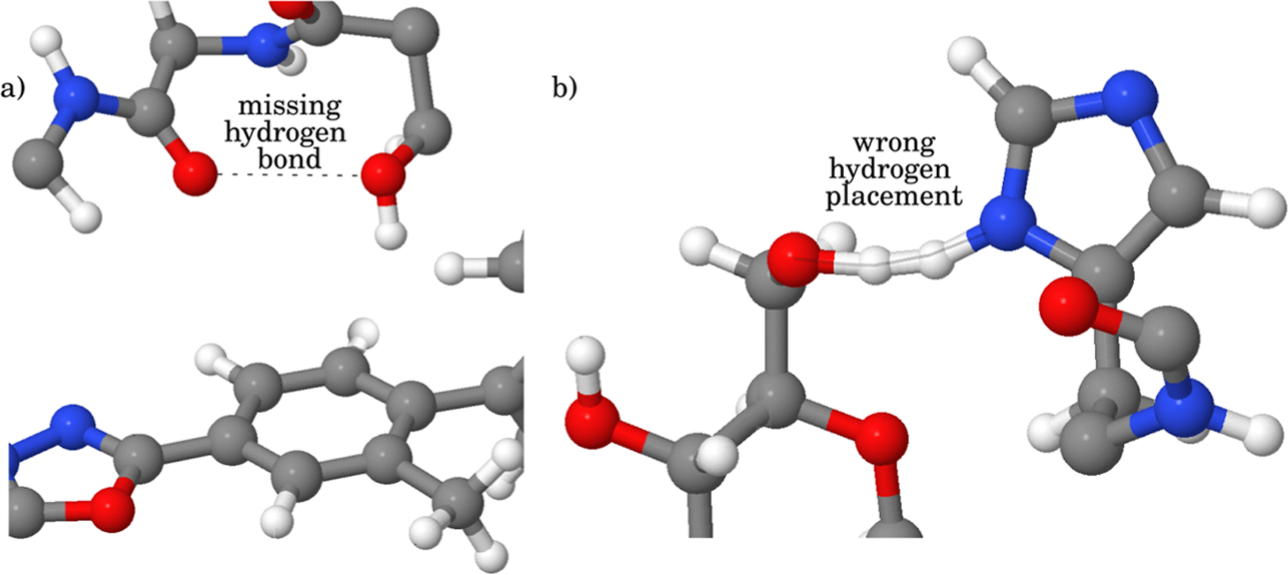
Examples of errors in hydrogen positions in PDB files. (a) A missing H-bond. (b) Clash of two polar hydrogens.

For each of the 94 complexes, AIP descriptions of the ligand and protein were created as described above, and the AIP pairing algorithm was used to calculate the free energy change for formation of the complex. Figure 9 compares the calculated free energies of binding with the experimental values. The AIP method predicts an absolute value of the binding free energy with reasonable accuracy: the root-mean-square error (RMSE) of 11 kJ mol^–1^ corresponds to a prediction of the ligand dissociation constant to within 2 log units. The binding affinity was underestimated by about 20 kJ mol^–1^ for 14 of the complexes, which are the main source of error in Figure 9. Compared with the other complexes, the 14 outliers are characterized by a larger number of contacts between nonpolar AIPs associated with CH groups and aromatic rings, but there were no other common features that could be identified to account for the large errors observed for these systems.

**Figure 9:**
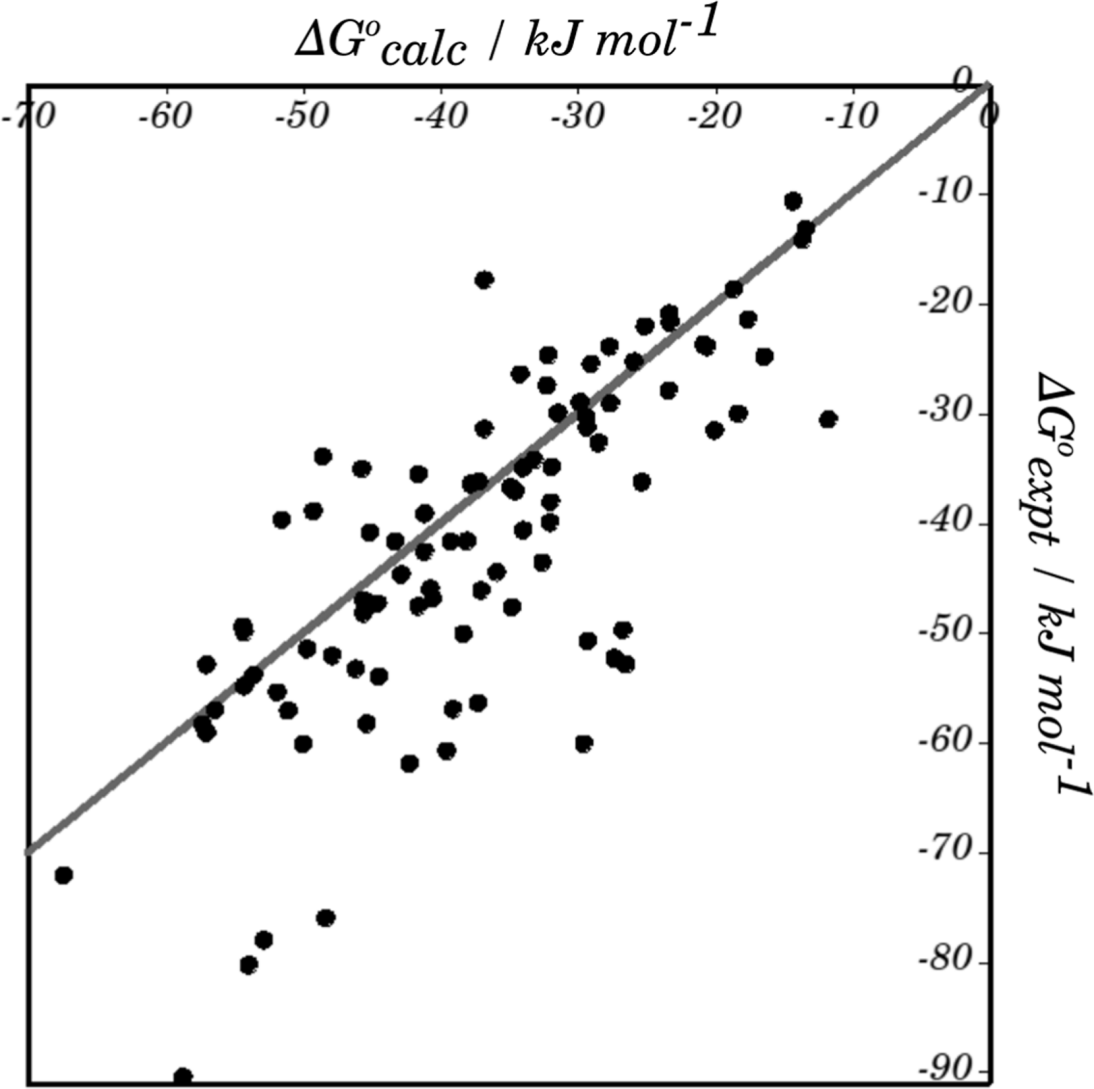
Comparison of calculated and experimental free energies of binding for 94 protein–ligand complexes. The black solid line is *y* = *x* (RMSE = 11 kJ mol^–1^).

Figure 10 shows the AIP Interaction Map for a complex (2W4X) that is well-described by the AIP approach: the experimental free energy of binding was −28 kJ mol^–1^ and the calculated value was −30 kJ mol^–1^. The individual AIP contacts calculated for 2W4X are listed in Table 1 (see Supporting Information for complete data set). Table 1 shows that although there are 6 intermolecular H-bonds, many are worth practically nothing due to the competition with solvent, and these H-bonding interactions contribute −6 kJ mol^–1^ in total to the binding free energy. The majority of the binding free energy comes from contacts between relatively nonpolar functional groups on the ligand and protein, and although each contact is worth a small amount, −1 to −2 kJ mol^–1^, there are a large number of them.

**Figure 10:**
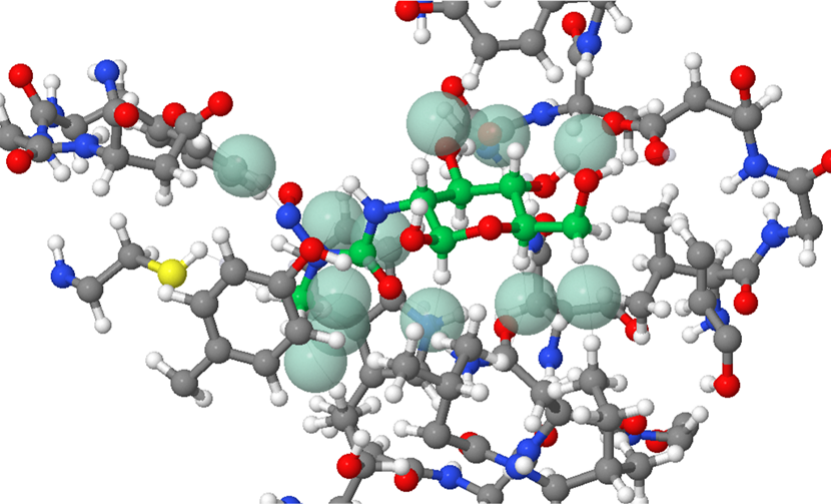
AIP Interaction Map for the 2W4X complex.^48^ Ligand carbons are colored bright green to aid identification. Green spheres highlight the most attractive AIP contacts (ΔΔ*G* < −0.5 kJ mol^–1^), and thin lines indicate the atoms associated with each AIP contact.

**Table 1:** AIP Contacts for the 2W4X Complex

contact type	ligand atom type	protein atom type	ligand AIP value	protein AIP value	*f* ^a^	ΔΔ*G*/kJ mol^–1^
	H.soft	C.ar	1.3	–1.5	0.5	–1.4
	N.2	C.2	–1.1	–0.5	0.5	–1.8
	H.soft	C.ar	1.2	–1.8	0.5	–1.3
H-bond	H.O	O.2.other	4.3	–5.5	1.0	–1.4
	C.2	C.ar	–0.4	–1.9	0.5	–1.3
	N.pl3.am	C.ar	2.1	–1.3	0.5	–0.4
H-bond	O.3.alcohol	H.O	–5.1	3.7	1.0	–0.5
	H.soft	S.3	1.3	0.0	0.5	–1.4
	H.soft	C.2	0.8	–1.7	0.5	–1.6
	H.soft	H.soft	1.0	0.3	1.0	–3.1
H-bond	H.O	O.2.am	4.3	–7.2	1.0	–3.8
H-bond	O.2.am	H.N	–4.4	2.6	1.0	0.0
	N.pl3.am	C.ar	1.4	–2.0	0.5	–1.1
	H.soft	H.soft	1.3	0.4	0.5	–1.2
	C.2	C.ar	0.3	–1.4	0.5	–1.8
	H.soft	C.ar	1.2	–1.7	0.5	–1.4
	H.soft	N.pl3.am	0.7	–0.4	0.5	–2.0
	H.soft	H.soft	0.7	0.3	0.5	–1.8
	H.soft	H.soft	1.0	0.9	1.0	–2.2
H-bond	O.3.alcohol	H.N	–3.4	2.6	1.0	0.0
	H.soft	S.3	1.3	–3.3	0.5	–0.5
H-bond	H.O	O.2.other	3.0	–5.5	1.0	–0.2

a AIPs that represent π-electron density have a fractional parameter *f* = 0.5, because they are associated with a smaller surface area.

The original CASF paper compared a number of different methods for analyzing protein–ligand complexes using four criteria: scoring, ranking, docking, and screening powers.^47^ For the 25 scoring functions tested, both the Pearson and Spearman’s rank correlation coefficients were generally in the range 0.4–0.6, and the highest values obtained were 0.8. Although the AIP approach was only tested on 94 of the 285 structures, the Pearson and Spearman rank correlation coefficients were both 0.76, which is comparable to the best method reported in the CASF paper. Moreover, the AIP method gives an absolute prediction of the value of the binding free energy and ligand dissociation constant.

## Conclusions

This paper describes a new approach to the analysis of protein–ligand interactions and the prediction of the absolute value of the dissociation constant for binding in water. Atomic surface site Interaction Points (AIP) are used to describe the surface of a molecule as a discrete set of points that encode the propensity for formation of noncovalent interactions, and the structure of an intermolecular complex can be analyzed for close contacts between the AIPs on the two molecular surfaces to compute the overall free energy change on binding. The approach was previously used to describe synthetic host–guest complexes, and in this paper, the AIP methodology was extended to macromolecular systems, protein–ligand complexes.

A fragment-based approach was developed to project AIP representations of repeating fragments onto the three-dimensional structure of a macromolecule, and application of this methodology to the amino acid functional groups present in proteins was used to create a picture of all possible noncovalent interaction sites available in a protein binding pocket. X-ray crystal structures of 94 protein–ligand complexes from the CASF benchmark data set were analyzed using this approach. Intermolecular interactions were identified as close contacts between AIPs on the ligand and AIPs on the protein. The free energy contribution due to each AIP contact was calculated using the SSIMPLE algorithm, which includes the contribution due to desolvation of the two interacting sites, and the sum of these free energy contributions was compared with the experimentally determined overall free energy of binding. Good agreement was obtained with a Pearson correlation coefficient of 0.76 and an RMSD of 11 kJ mol^–1^ for the absolute values of the free energy of binding. In addition, the AIP analysis provides explicit energetic decomposition into functional group interactions and desolvation contributions, which allows detailed analysis of the role of different parts of the ligand in binding.

The current implementation of the AIP analysis is based on the sum of two free energy contributions, pairwise protein–ligand contacts and desolvation of those sites, but there are a number of additional free energy contributions that are likely to affect the observed dissociation constant for a protein–ligand complex. Both the protein and ligand are dynamic and conformational flexibility either or both may change on binding, and these entropic contributions to binding affinity are not included the AIP method described here. Moreover, a static X-ray crystal structure is used for the AIP analysis of intermolecular contacts, whereas in solution complexes may populate more than one binding mode, which would lead to variability in the set of intermolecular contacts. The SSIMPLE treatment of solvation assumes that there is no crosstalk between neighboring interaction sites on the molecular surfaces, but very small hydrophobic pockets that restrict the packing of water molecules can lead to partial desolvation, and this phenomenon can only be captured by approaches that simulate the complete solvation shell in atomic detail. Similarly, all intermolecular contacts are treated as independent of one another, and the cooperative effects associated with polarization in H-bonded networks are missing. The agreement between calculation and experiment based only on X-ray structure AIP contacts is surprisingly good given these approximations, but the development of a more reliable and accurate method will require approaches for calculating the free energy contributions due all of these additional features of protein–ligand complexes.

In addition, there are some features of the current AIP implementation that limit the generality of the approach. First, a DFT calculation is required to obtain the ligand AIP description, and although this calculation is fast for individual ligands, the current methods are not practical for the large libraries used in virtual screening. Machine-learning approaches to the calculation of accurate molecular electrostatic potential energy surfaces show some promise in fast footprinting and may provide a solution to this problem in the future.^49^ Second, there is no method for footprinting charged compounds or for estimating the interaction energy for a contact involving two AIPs that represent charged functional groups. The treatment of ionic interactions in a protein binding site is a major challenge, because the interplay of short-range interactions and the overall Coulombic interaction associated with ion-pairing depends on the effective local dielectric constant, and the extent of ion-pairing with counterions in the free state is not known. In the methodology described here for footprinting the proteins, the charged groups are treated as nonionic isosteres, and it might be possible to implement something similar for the ligands. However, it is not clear that simply avoiding charge represents a good solution, and a more detailed study will be required to develop a reliable model of ionic interactions within the AIP framework.

## Supplementary Material





## Data Availability

The protein–ligand crystal structures were taken from ref 47. A pdf with calculated and experimental free energies of binding for all protein–ligand complexes, list of all AIP contacts for each protein–ligand complex is provided as Supporting Information, along with an xml file formatted as text, protein_aip_ff.txt, containing the OpenMM force-field for adding AIPs to protein structures. The software used to calculate AIPs for ligands and to analyze AIP contacts in X-ray crystal structures is available free of charge at https://github.com/k-zator/AIP_map and https://github.com/k-zator/AIP.

## Notes

A pdf containing details of calculation methods, corrected hydrogen positions corrected in pdb files, calculated and experimental free energies of binding for all protein–ligand complexes, list of all AIP contacts for each protein–ligand complex. An xml file formatted as text, protein_aip_ff.txt, containing the OpenMM force-field for adding AIPs to protein structures. The software used to calculate AIPs for ligands and to analyze AIP contacts in X-ray crystal structures is available free of charge at https://github.com/k-zator/AIP_map and https://github.com/k-zator/AIP (PDF)
(TXT)

## †

Astex Pharmaceuticals, 436 Cambridge Science Park, Milton Road, Cambridge, CB4 0QA, U.K
