# Supplementary material for: Prediction of Protein–Ligand Binding Affinities Using Atomic Surface Site Interaction Points
Source: J Chem Inf Model. 2026 Jan 14;66(2):1097–105. doi: 10.1021/acs.jcim.5c02628 (PMC12848979; doi:10.1021/acs.jcim.5c02628)
Supplement: Supplementary file 1 [file ci5c02628_si_001.pdf]

Supplementary Information

Prediction of Protein-Ligand Binding Affinities Using Atomic Surface Site Interaction Points

Katarzyna J. Zator, Maria Chiara Storer, and Christopher A. Hunter*

Yusuf Hamied Department of Chemistry, University of Cambridge, CB2 1EW, UK

SI.revised.pdf: methods used to footprint ligands and proteins, calculated and experimental free energies of binding for all protein-ligand complexes, list of all AIP contacts for each protein-ligand complex.

protein_aip_ff.txt: an xml file formatted as text containing the OpenMM force-field for adding AIPs to protein structures.

1 Footprinting of the ligand

For footprinting,[1] the geometries of the complexes were obtained from the Supplementary Information of the original CASF by Wang et. al.[2] The ligand structures were converted to PDB format using OpenBabel 3.0.1.[3] The MEPS of the ligand was calculated with NWChem 7.0.2,[4] which contained a Python script to calculate the specific isosurfaces, (https://github.com/k-zator/AIP). To maintain the X-ray geometry, no geometry optimisation was carried out on each molecule using a DRIVER module with up to 600 iterations and a convergence cri-terion of 1e-08 (B3LYP functional and 6-31G* basis set, or 6-31G** basis set for iodine without effective core potentials). The MEPS was then calculated using the espiso function in NWChem[4] with the following parameters:

1. 0.0020 isosurface: padding: 4.0, iso¯surf: 0.002, step¯size: 0.066, tol: 3e-5

2. 0.0104 isosurface: padding: 4.0, iso¯surf: 0.0104, step¯size: 0.04, tol: 1e-4

3. 0.0300 isosurface: padding: 4.0, iso¯surf: 0.0300, step¯size: 0.04, tol: 1e-4

For each structure, seven different rotations were carried out (combinations of 45° and 120° in one plane and 10° and 100° in another), and the MEPS calculations were repeated. The seven rotated MEPS were then superimposed to obtain high-resolution MEPS for analysis. The footprinting algorithm finally required a formatted CML ligand file containing atom types, which can be obtained with AIP's cmlgenerator module.[5] The three cube files and the CML files were then read in by the main AIP_footprinting script to produce an output XML file containing all the resultant ligand AIPs with their coordinates and values.

The AIP footprinting algorithm determines an optimum location of AIPs as well as calculates their values depending on the atom type-encoded functional groups according to a linear relationship with MEP value:

$$\epsilon_i = m \times E_{min/max} + c_\beta \tag{1}$$

where the m and c parameters were determined by linear regression for the following atom types. Whether E_{min} or E_{max} is used depends on whether the AIP is a donor or an acceptor. Polar acceptor atom types use the 0.030 e $Bohr^{-3}$ isosurface, and m of -0.0336 kJ mol^{-1}, and c_β values in Table S1.

Non-polar acceptors use the 0.002 e $Bohr^{-3}$ isosurface and have m = -0.0232 kJ mol^{-1} and c = 0. Donors were likewise divided into polar and non-polar; however, they used the 0.0104 e $Bohr^{-3}$ isosurface. Polar donors used a maximum of MEPS with m = 0.0132 kJ mol^{-1} and c = -2.80, whereas non-polar donors used m = 0.0078 kJ mol^{-1}, c = -0.64, respectively. Further details on the procedure can be found in the relevant publication.[1]

2 Footprinting of the protein

The protein AIPs were determined using the compiled AIP descriptions available within the AIP_map code in the dedicated GitHub repository. Therefore, the calculation did not produce a separate complex XML file for the protein to be read in by the AIP_map mod-

Table S1: List functional group c_β values for AIP value calculation.

Functional group	c_β
Nitrile	-0.64
Pyridine, imine	-1.08
Aniline	-2.57
Primary amine	-2.94
Secondary amine	-1.63
Tertiary amine	-0.98
Ketone, aldehyde, ester, carboxylic acid	-0.42
Amide, urea, carbamate, amidate	0.44
N-oxide	1.51
Nitro	-1.33
Phosphine oxide	2.03
Sulfoxide, sulfone	0.44
Alcohol	-1.57
Phenol, ether, epoxide	-0.66
Fluoro	0.31
Phosphine sulfide	4.52
Thionyl	2.94

ule. Instead, it created a representation of the AIPs in an OpenMM force field format as: ”ligand_vs.ff” and ”ligand_custom.xml” files, which were subsequently read in by the AIP_interaction_map.score module. The calculation also created a ”binding_site” PDB file, which was to be used instead of the relevant MOL2 file.

3 Protein-ligand AIP pairing procedure

The resultant AIP file, along with PDB files containing the original geometry, was used to identify AIP pairings with the AIP map script[6] as can be found on GitHub: https://github.com/k-zator/AIP_map. The notable difference was in using the ”–protein” flag to ensure the use of the pre-computed AIP descriptions for amino acids. The identification of the AIP contacts was done by a dedicated module that identified the close AIP-AIP distances between the two structures and determined how these should be paired. The algorithm contains three main steps: the protein-ligand H-bond identification, the protein-protein H-bond identification, and finally, the identification of other interactions. The H-bonds were primarily studied due

to their larger AIP values, and, therefore, the incorrect pairing of such AIPs could lead to large errors. The H-bonds are designated as D...H-A, where D, A ϵ O, N. The secondary H-bond identification ensured that the relevant AIPs that are found near the ligand binding site indeed can interact with the ligand, and are not already interacting with another AIP. Finally, the other contacts were found using a custom maximum bipartite matching algorithm,[6] which deduced the optimal pairing combinations to maximise the number of contacts and the binding energy of the complex. It depends only on the distance cut-off variable, d_{max}, which was set to 1.7 Å.

The AIP values were then used to calculate the free energy of binding contribution from an AIP contact. The interaction energy was scaled depending on the surface area of contacts for different atom types and their polarity. The scaling fractions that accomplish this, f, are compiled in Table S2:

Table S2: Average surface areas associated with non-polar AIP sites and corresponding scaling factors (f).

AIP type	Scaling factor, f
Carbon π-system	0.50
Nitrogen π-system	0.25
Oxygen π-system	0.00
Chlorine p-orbital	0.50
Bromine p-orbital	0.75
Iodine p-orbital	1.00
Sulfur π-system or σ-hole	0.50

4 PDBs that required hydrogen position readjustment

Table S3: List of PDBs and identity of hydrogens for whom the automated hydrogen placement with SYBYL software led to incorrect geometrical placement, leading to steric clashes with ligand atoms.

PDB ID	Hydrogen requiring position adjustment
1NC1	HG SER A 196
1NC3	HG SER A 76
1Z95	HG1 THR B 877
2POG	HD1 HIS B 524
2POG	HE2 HIS B 524
3B65	HG1 THR A 877
3GNW	HG SER B 368
3JVS	HH TYR A 173
4CR9	HG SER A 195
4IH5	HH TYR A 448
4IH7	HG SER A 368
4IH7	HH TYR A 448
4RFM	HG SER A 499

5 CASF protein-ligand complexes

PDB ID	ΔG^o_{expt} / (kJ/mol)	ΔG^o_{calc} / (kJ/mol)
1bcu	-18.72	-18.60
1e66	-56.45	-56.98
1g2k	-45.44	-58.23
1nc1	-34.93	-36.67
1nc3	-28.54	-32.62
1pxn	-40.81	-45.94
1qkt	-51.60	-39.65
1r5y	-36.87	-17.77
1s38	-29.40	-31.19

1z95	-40.64	-46.81
2br1	-29.34	-50.66
2brb	-27.74	-23.86
2c3i	-43.38	-41.64
2cet	-45.78	-34.98
2fxs	-34.59	-37.02
2iwx	-38.13	-41.58
2pog	-54.45	-49.36
2p15	-58.79	-90.44
2qe4	-45.44	-47.43
2v00	-20.89	-23.68
2vvn	-41.67	-35.50
2w4x	-27.68	-29.03
2wbg	-25.40	-36.18
2wca	-31.96	-34.83
2wn9	-48.63	-33.89
2xdl	-17.69	-21.37
2yki	-54.00	-80.23
3ao4	-11.82	-30.49
3aru	-18.38	-29.97
3arv	-32.19	-24.65
3ary	-34.25	-26.35
3b1m	-48.40	-75.94
3b27	-29.45	-30.22
3b5r	-50.06	-60.11
3b65	-52.91	-77.95
3b68	-47.95	-52.04

3e92	-45.66	-48.18
3fur	-45.66	-46.97
3g0w	-54.34	-49.88
3g2n	-23.35	-21.66
3g2z	-13.47	-13.16
3gnw	-51.94	-55.36
3gr2	-14.38	-10.60
3jvr	-32.65	-43.54
3jvs	-37.33	-56.32
3jya	-39.33	-41.65
3k5v	-35.96	-44.42
3kr8	-46.24	-53.23
3l7b	-13.70	-14.12
3o9i	-67.47	-72.07
3p5o	-41.67	-47.51
3prs	-44.64	-47.27
3pyy	-39.16	-56.90
3rlr	-42.92	-44.63
3rr4	-25.97	-25.21
3rsx	-25.17	-21.99
3syr	-29.11	-25.44
3u5j	-32.02	-38.01
3wz8	-33.22	-34.18
3wtj	-37.27	-36.15
4bkt	-20.66	-23.88
4cr9	-23.40	-20.84
4cra	-41.21	-42.57

4de1	-34.02	-34.91
4de3	-31.51	-29.91
4dli	-32.08	-39.91
4eky	-20.09	-31.49
4f9w	-39.61	-60.72
4gfm	-41.21	-39.10
4hge	-45.21	-40.83
4ih5	-23.46	-27.85
4ih7	-29.91	-28.91
4ivb	-49.77	-51.41
4ivc	-57.08	-52.85
4ivd	-54.34	-54.79
4j21	-44.52	-53.93
4j3l	-42.30	-61.87
4k18	-51.14	-57.04
4k77	-37.84	-36.37
4kzq	-34.82	-47.60
4kzu	-37.10	-46.08
4llx	-16.50	-24.76
4lzs	-27.40	-52.25
4m0y	-36.87	-31.34
4m0z	-29.62	-60.05
4mgd	-26.77	-49.68
4qac	-53.66	-53.80
4qd6	-49.32	-38.87
4rfm	-57.37	-58.29
4twp	-57.08	-59.08

4w9c	-26.54	-52.82
4w9h	-38.42	-50.04
4w9i	-34.02	-40.60
5c28	-32.31	-27.40

Table S4: Protein-ligand complexes from the CASF dataset used in this thesis.

6 AIP contacts for the CASF protein-ligand complexes

Table S5: 1BCU protein-ligand complex

Ligand atom number	Ligand atom type	Ligand AIP value	Protein atom number	Protein atom type	Protein AIP value	f	$\Delta\Delta G$ (kJ/mol)
5	C.ar	-1.59	643	H.soft	0.34	0.5	-1.71
20	H.soft	0.99	639	C.2	-0.5	0.5	-1.72
7	C.ar	-1.64	646	H.soft	0.34	0.5	-1.68
8	C.ar	-1.42	642	H.soft	1.11	0.5	-1.51
11	C.ar	-1.42	642	H.soft	1.11	0.5	-1.51
13	C.ar	-2.77	633	N.pl3.am	-0.48	0.5	-0.39
2	C.ar	-1.72	590	C.2	-1.76	0.5	-0.52
3	C.ar	-3.14	591	O.2.am	-1.35	0.5	0.62
9	N.ar	-2.64	617	H.soft	0.64	0.5	-0.98
10	C.ar	-2.99	617	H.soft	0.64	0.5	-0.68
12	C.ar	-1.45	613	C.2	-1.76	0.5	-0.74
0	C.ar	-1.98	897	H.soft	1.11	0.5	-1.3
1	C.ar	-2.01	885	C.2	-1.76	0.5	-0.26
17	H.soft	1.02	883	N.pl3.am	-0.38	0.5	-1.69
17	H.soft	1.02	833	H.soft	0.33	0.5	-1.5
3	C.ar	-3.11	948	H.soft	0.88	0.5	-0.62
12	C.ar	-1.61	943	N.pl3.am	-0.38	0.5	-1.51
24	H.N	2.32	560	O.2.other	-5.5	1.0	0.57

Table S6: 1E66 protein-ligand complex

Ligand atom number	Ligand atom type	Ligand AIP value	Protein atom number	Protein atom type	Protein AIP value	f	$\Delta\Delta G$ (kJ/mol)
2	C.ar	-1.36	1451	C.ar	-1.52	0.5	-1.0
3	C.ar	-2.24	1450	C.ar	-1.44	0.5	-0.3
4	C.ar	-2.25	419	N.ar.no_lp	-1.17	0.5	-0.49
5	C.ar	-2.07	420	C.ar	-1.67	0.5	-0.28
14	C.ar	-0.85	418	C.ar	-1.82	0.5	-1.1
15	C.ar	-0.63	416	C.ar	-1.57	0.5	-1.42
16	C.ar	-0.9	417	C.ar	-1.53	0.5	-1.3
17	C.ar	-1.2	315	H.soft	0.64	0.5	-1.83
35	H.soft	0.67	421	C.ar	-1.98	0.5	-1.45
35	H.soft	0.67	423	C.ar	-1.99	0.5	-1.44
20	Cl	-1.65	1855	H.soft	0.33	0.5	-1.68
20	Cl	-1.69	1723	C.ar	-1.67	0.5	-0.62
9	C.2	-1.41	643	H.soft	0.88	0.5	-1.65
38	H.soft	0.66	639	C.2	-1.76	0.5	-1.58
38	H.soft	0.66	731	C.ar	-1.53	0.5	-1.69
16	C.ar	-1.01	1455	C.ar	-1.56	0.5	-1.21
3	C.ar	-2.3	1958	H.soft	0.94	0.5	-1.2
30	H.soft	0.61	1305	C.ar	-1.56	0.5	-1.69
30	H.soft	0.61	741	H.soft	0.85	0.5	-1.49
0	N.ar	-2.24	1452	C.ar	-1.53	0.5	-0.23
5	C.ar	-2.35	1454	C.ar	-1.56	0.5	-0.1
8	C.2	-1.98	1894	H.soft	0.72	0.5	-1.44
9	C.2	-1.97	1463	H.soft	0.71	0.5	-1.45
24	H.soft	0.63	1886	C.ar	-2.17	0.5	-1.33
24	H.soft	0.63	1882	O.2.am	-1.35	0.5	-1.78
8	C.2	-1.46	637	N.pl3.am	-0.38	0.5	-1.61
25	H.soft	0.55	424	C.ar	-1.96	0.5	-1.48
25	H.soft	0.55	1927	H.soft	0.76	0.5	-1.61
17	C.ar	-1.27	1453	C.ar	-1.55	0.5	-1.05
32	H.soft	0.64	655	N.pl3.am	-0.38	0.5	-1.98
32	H.soft	0.64	659	H.N	2.84	0.5	2.05
33	H.soft	1.6	428	H.soft	0.46	1.0	-1.23
31	H.soft	0.57	1513	H.soft	0.69	0.5	-1.66
31	H.soft	0.57	1504	C.ar	-1.56	0.5	-1.7
20	Cl	0.0	1818	H.soft	0.82	0.5	-1.85
20	Cl	0.0	1721	C.ar	-1.82	0.5	-1.54
18	C.ar	-1.23	1735	H.soft	0.81	0.5	-1.75
20	Cl	-1.35	1599	H.soft	0.94	0.5	-1.64
37	H.soft	1.09	1727	C.ar	-1.96	0.5	-1.32
37	H.soft	1.09	1592	O.3.any	-3.72	0.5	-0.16
1	N.pl3.aniline	0.41	427	H.soft	0.49	0.5	-1.9
20	Cl	-1.79	1457	H.soft	0.64	0.5	-1.57

Table S7: 1G2K protein-ligand complex

Ligand atom number	Ligand atom type	Ligand AIP value	Protein atom number	Protein atom type	Protein AIP value	f	$\Delta\Delta G$ (kJ/mol)
60	H.soft	1.2	1828	H.soft	0.23	1.0	-2.69
62	H.soft	1.06	1817	N.pl3.am	-0.38	0.5	-1.65
16	C.ar	-1.8	1512	H.soft	0.64	0.5	-1.56
17	C.ar	-2.04	1541	H.N	2.84	0.5	0.48
18	C.ar	-2.05	1541	H.N	2.84	0.5	0.48
24	C.ar	-1.14	1803	H.soft	0.76	0.5	-1.8
25	C.ar	-1.26	1801	C.2	-1.76	0.5	-0.88
38	C.ar	-1.41	2092	H.soft	0.48	0.5	-1.79
19	C.ar	-2.09	1575	H.N	2.84	0.5	0.46
20	C.ar	-2.07	1567	O.2.am	-7.73	0.5	7.5
21	C.ar	-2.02	1513	H.soft	0.49	0.5	-1.44
11	C.ar	-1.87	404	H.soft	0.49	0.5	-1.54
27	C.ar	-1.56	1015	H.soft	0.33	0.5	-1.73
26	C.ar	-1.39	1015	H.soft	0.33	0.5	-1.82
58	H.soft	0.94	1573	H.soft	0.49	0.5	-1.48
58	H.soft	0.94	2028	H.soft	0.34	0.5	-1.58
21	C.ar	-1.72	1757	H.soft	0.33	0.5	-1.63
20	C.ar	-1.83	1757	H.soft	0.33	0.5	-1.56
75	H.soft	0.96	732	H.soft	0.76	0.5	-1.24
75	H.soft	0.96	2091	H.soft	0.34	0.5	-1.56
13	C.ar	-1.09	1831	H.soft	0.33	0.5	-1.95
8	C.ar	-0.82	1831	H.soft	0.33	0.5	-2.03
25	C.ar	-1.11	1016	H.soft	0.33	0.5	-1.94
30	O.3.alcohol	-1.45	382	C.2	-1.76	0.5	-0.74
30	O.3.alcohol	-1.45	398	N.pl3.am	-0.38	0.5	-1.62
23	C.ar	-1.5	1087	H.soft	0.34	0.5	-1.76
24	C.ar	-0.76	1087	H.soft	0.34	0.5	-2.05
10	C.ar	-0.94	403	H.soft	0.64	0.5	-1.91
29	O.3.alcohol	-1.0	1507	N.pl3.am	-0.38	0.5	-1.88
29	O.3.alcohol	-1.0	1491	C.2	-1.76	0.5	-1.06
49	H.soft	0.91	733	H.soft	0.88	1.0	-2.36
35	C.ar	-1.37	2122	H.soft	0.33	0.5	-1.83
12	C.ar	-0.91	1090	H.soft	0.33	0.5	-2.01
13	C.ar	-0.65	405	H.soft	0.49	0.5	-2.02
16	C.ar	-1.48	760	H.soft	0.33	0.5	-1.77
19	C.ar	-1.86	1752	H.soft	0.22	0.5	-1.54
53	H.soft	1.1	531	H.soft	0.33	1.0	-2.82
12	C.ar	-1.17	530	H.soft	0.33	0.5	-1.92
46	H.soft	1.32	2190	H.soft	0.34	1.0	-2.24
39	O.2.sulfone	-5.73	1835	H.N	2.84	1.0	-0.04
14	O.2.sulfone	-4.12	764	H.N	2.84	1.0	0.05
42	O.2.am	-7.78	466	H.N	2.84	1.0	0.02
68	H.O	4.32	322	O.2.other	-5.5	1.0	-1.4
67	H.O	4.35	1431	O.2.other	-5.5	1.0	-1.43

Table S8: 1NC1 protein-ligand complex

Ligand atom number	Ligand atom type	Ligand AIP value	Protein atom number	Protein atom type	Protein AIP value	f	$\Delta\Delta G$ (kJ/mol)
13	C.ar	-0.83	742	H.soft	0.71	0.5	-1.89
14	C.ar	-0.59	736	C.ar	-1.56	0.5	-1.45
16	N.ar	0.07	776	N.pl3.am	-0.38	0.5	-2.17
17	C.ar	-1.94	734	C.ar	-1.53	0.5	-0.52
17	C.ar	-2.1	1002	C.2	-2.23	0.5	0.25
18	N.ar	-1.19	1009	H.soft	1.11	0.5	-1.56
18	N.ar	-1.06	741	H.soft	0.68	0.5	-1.86
18	N.ar	-5.33	1051	H.soft	0.65	1.0	3.62
19	C.ar	-1.41	1009	H.soft	1.11	0.5	-1.51
23	H.soft	0.94	1045	S.3	0.0	0.5	-1.75
35	H.soft	0.84	738	H.soft	0.64	1.0	-2.94
10	N.ar.no_lp	-1.57	458	H.soft	0.64	0.5	-1.68
11	C.ar	-1.43	455	C.2	-1.76	0.5	-0.76
12	C.ar	-1.49	476	N.pl3.am	-0.38	0.5	-1.59
13	C.ar	-1.22	970	O.2.am	-1.35	0.5	-1.23
14	C.ar	-2.28	976	H.soft	0.33	0.5	-1.23
16	N.ar	-1.35	788	H.soft	0.34	0.5	-1.84
31	H.soft	1.44	429	C.2	-1.76	0.5	-1.15
31	H.soft	1.44	453	N.pl3.am	-0.38	0.5	-1.21
30	H.soft	1.03	430	O.2.am	-1.35	0.5	-1.58
26	H.soft	0.71	1039	N.pl3.am	-0.38	0.5	-1.93
22	H.soft	1.16	1772	C.ar	-1.56	0.5	-1.43
22	H.soft	1.16	1538	C.ar	-1.56	0.5	-1.43
28	H.soft	1.14	258	H.soft	0.33	0.5	-1.36
28	H.soft	1.14	1045	S.3	-3.5	0.5	-0.34
11	C.ar	-1.85	1546	H.soft	0.7	0.5	-1.52
24	H.soft	0.8	1539	C.ar	-1.56	0.5	-1.63
34	H.N	2.19	1316	O.2.other	-5.5	1.0	0.75
16	N.ar	-6.62	794	H.N	2.84	1.0	-0.02
6	O.3.alcohol	-4.58	1055	H.N	2.84	1.0	-0.01
6	O.3.alcohol	-4.18	1189	H.N	2.7	1.0	-0.0
29	H.O	3.99	1085	O.2.other	-5.5	1.0	-1.12
27	H.O	4.01	1084	O.2.other	-5.5	1.0	-1.14

Table S9: 1NC3 protein-ligand complex

Ligand atom number	Ligand atom type	Ligand AIP value	Protein atom number	Protein atom type	Protein AIP value	f	$\Delta\Delta G$ (kJ/mol)
0	N.ar	1.42	646	C.ar	-1.53	0.5	-1.22
1	C.ar	-1.34	650	H.soft	0.64	0.5	-1.78
4	C.ar	0.68	654	H.soft	0.71	0.5	-1.56
5	C.ar	0.42	648	C.ar	-1.56	0.5	-1.72
7	N.ar.no_lp	-0.23	654	H.soft	0.71	0.5	-1.93
8	N.ar	0.63	1449	C.ar	-1.55	0.5	-1.69
21	H.N	3.03	691	O.2.am	-7.73	0.5	-0.31
21	H.N	3.03	688	N.pl3.am	-0.38	0.5	1.71
1	C.ar	-1.84	914	C.2	-2.23	0.5	0.01
2	N.ar	-1.45	921	H.soft	1.11	0.5	-1.5
2	N.ar	-5.49	963	H.soft	0.65	0.5	2.0
2	N.ar	-0.25	957	S.3	0.0	0.5	-2.2
3	C.ar	-1.58	921	H.soft	1.11	0.5	-1.46
9	C.ar	-1.49	370	H.soft	0.64	0.5	-1.72
24	H.soft	0.33	951	N.pl3.am	-0.38	0.5	-2.11
24	H.soft	0.33	963	H.soft	0.65	0.5	-1.84
30	H.soft	0.55	653	H.soft	0.68	1.0	-3.36
5	C.ar	-1.54	882	O.2.am	-1.35	0.5	-1.0
4	C.ar	0.13	388	N.pl3.am	-0.38	0.5	-2.16
7	N.ar.no_lp	-0.73	367	C.2	-1.76	0.5	-1.22
8	N.ar	0.08	365	N.pl3.am	-0.38	0.5	-2.17
26	H.soft	0.86	957	S.3	-3.5	0.5	-0.28
26	H.soft	0.86	213	H.soft	0.33	0.5	-1.66
29	H.soft	0.63	1451	C.ar	-1.56	0.5	-1.69
23	H.soft	0.47	342	O.2.am	-1.35	0.5	-1.82
23	H.soft	0.47	341	C.2	-1.76	0.5	-1.61
20	H.N	3.73	1228	O.2.other	-5.5	1.0	-0.89
12	O.3.alcohol	-6.02	967	H.N	2.84	1.0	-0.03
25	H.O	2.91	997	O.2.other	-5.5	1.0	-0.11
0	N.ar	-5.29	706	H.N	2.84	1.0	-0.04
12	O.3.alcohol	-5.72	1101	H.N	2.7	1.0	0.14
22	H.N	4.29	1229	O.2.other	-5.5	1.0	-1.38
8	N.ar	-5.53	1205	H.O	2.88	1.0	-0.08
27	H.O	3.57	996	O.2.other	-5.5	1.0	-0.75

Table S10: 1PXN protein-ligand complex

Ligand atom number	Ligand atom type	Ligand AIP value	Protein atom number	Protein atom type	Protein AIP value	f	$\Delta\Delta G$ (kJ/mol)
19	C.ar	-2.01	1160	H.soft	1.11	0.5	-1.29
30	H.soft	1.03	985	O.2.am	-7.21	1.0	6.37
12	N.ar	-0.52	1027	C.ar	-1.52	0.5	-1.51
13	C.ar	-1.81	1032	H.soft	0.64	0.5	-1.55
11	C.ar	-1.35	1600	H.soft	0.33	0.5	-1.84
14	N.pl3.aniline	-1.11	1600	H.soft	0.33	0.5	-1.94
17	C.ar	-2.12	195	H.soft	0.33	0.5	-1.36
16	C.ar	-3.38	195	H.soft	0.33	0.5	-0.18
12	N.ar	-0.74	1079	H.soft	0.34	0.5	-2.05
13	C.ar	-1.76	1601	H.soft	0.33	0.5	-1.61
1	C.ar	-1.53	437	H.soft	0.33	0.5	-1.75
2	N.ar	-0.28	440	H.soft	0.33	0.5	-2.12
4	N.pl3.aniline	-0.7	440	H.soft	0.33	0.5	-2.06
6	C.ar	-1.77	437	H.soft	0.33	0.5	-1.6
14	N.pl3.aniline	-1.74	1029	C.ar	-1.55	0.5	-0.68
6	C.ar	-1.07	1602	H.soft	0.33	0.5	-1.96
8	C.ar	-0.89	1602	H.soft	0.33	0.5	-2.01
16	C.ar	-1.99	1192	N.pl3.am	-0.48	0.5	-1.16
17	C.ar	-1.99	1202	H.soft	0.49	0.5	-1.46
10	N.ar	-1.32	196	H.soft	0.33	0.5	-1.85
20	C.ar	-1.83	194	H.soft	0.33	0.5	-1.56
19	C.ar	-2.14	194	H.soft	0.33	0.5	-1.34
24	H.soft	0.87	942	C.ar	-1.56	0.5	-1.6
23	H.soft	0.78	1706	H.soft	0.49	1.0	-3.27
18	C.ar	-2.0	1151	N.pl3.am	-0.38	0.5	-1.2
34	H.soft	1.04	1110	C.2	-1.76	0.5	-1.43
34	H.soft	1.04	1031	C.ar	-1.56	0.5	-1.51
28	H.soft	1.29	223	H.soft	0.88	1.0	-1.41
27	H.soft	1.44	1482	H.soft	0.26	1.0	-2.0
29	H.soft	1.1	758	H.soft	0.33	1.0	-2.82
8	C.ar	-1.8	650	H.soft	0.49	0.5	-1.58
32	H.soft	0.64	1599	H.soft	0.34	1.0	-3.67
12	N.ar	-6.45	1088	H.N	2.84	1.0	-0.02
21	O.3.any	-4.08	1326	H.N	2.7	1.0	-0.0
31	H.N	2.03	1073	O.2.am	-7.73	1.0	2.91

Table S11: 1QKT protein-ligand complex

Ligand atom number	Ligand atom type	Ligand AIP value	Protein atom number	Protein atom type	Protein AIP value	f	$\Delta\Delta G$ (kJ/mol)
0	C.ar	-1.66	404	H.soft	0.49	0.5	-1.66
4	C.ar	-1.85	943	H.soft	0.33	0.5	-1.55
10	C.ar	-1.71	1144	H.soft	0.71	0.5	-1.59
20	H.soft	0.95	257	O.2.am	-1.35	0.5	-1.63
31	H.soft	0.71	256	C.2	-1.76	0.5	-1.57
31	H.soft	0.71	264	H.soft	0.33	0.5	-1.79
1	C.ar	-1.61	812	H.soft	0.33	0.5	-1.7
2	C.ar	-1.45	807	H.soft	0.33	0.5	-1.79
4	C.ar	-1.63	807	H.soft	0.33	0.5	-1.69
35	H.soft	0.61	1383	H.soft	0.33	0.5	-1.86
35	H.soft	0.61	1343	H.soft	0.82	0.5	-1.51
1	C.ar	-1.9	1143	H.soft	0.68	0.5	-1.5
36	H.soft	0.63	695	H.soft	0.34	1.0	-3.69
21	H.soft	1.1	370	H.soft	0.19	0.5	-1.49
21	H.soft	1.1	397	N.pl3.am	-0.38	0.5	-1.61
38	H.soft	0.6	1706	H.soft	0.49	1.0	-3.56
28	H.soft	0.58	698	H.soft	0.33	1.0	-3.77
24	H.soft	0.67	941	H.soft	0.34	1.0	-3.63
22	H.O	3.79	515	O.2.other	-5.5	1.0	-0.94
3	O.3.any	-3.85	1021	H.N	2.7	1.0	0.01
18	O.3.alcohol	-5.35	1709	H.N	3.44	1.0	-0.54

Table S12: 1R5Y protein-ligand complex

Ligand atom number	Ligand atom type	Ligand AIP value	Protein atom number	Protein atom type	Protein AIP value	f	$\Delta\Delta G$ (kJ/mol)
3	C.ar	-2.16	1254	H.soft	0.53	0.5	-1.34
5	N.ar	-0.42	1259	H.soft	0.81	0.5	-1.87
0	C.ar	-2.84	267	C.ar	-1.44	0.5	0.33
2	C.ar	-2.35	269	C.ar	-1.32	0.5	-0.28
12	C.ar	-2.3	276	H.soft	0.94	0.5	-1.2
3	C.ar	-2.46	264	C.ar	-1.42	0.5	-0.1
4	C.ar	-2.47	265	C.ar	-1.45	0.5	-0.06
5	N.ar	-1.72	272	H.soft	0.51	0.5	-1.62
6	C.ar	-1.92	272	H.soft	0.51	0.5	-1.5
9	C.ar	-2.45	277	H.soft	0.7	0.5	-1.13
11	C.ar	-2.22	277	H.soft	0.7	0.5	-1.29
0	C.ar	-1.89	1253	H.soft	0.51	0.5	-1.52
6	C.ar	-1.35	706	H.soft	0.34	0.5	-1.84
7	N.pl3.aniline	-0.21	706	H.soft	0.34	0.5	-2.12
8	N.ar.no_lp	0.28	710	H.soft	0.33	0.5	-2.04
9	C.ar	-2.51	1250	S.3	-3.5	0.5	2.05
11	C.ar	-2.1	1250	S.3	-3.5	0.5	1.66
12	C.ar	-2.44	1253	H.soft	0.51	0.5	-1.13
2	C.ar	-2.08	1246	C.2	-1.76	0.5	-0.2
15	H.soft	0.95	1287	H.soft	0.88	1.0	-2.27
5	N.ar	-6.73	174	H.O	3.68	1.0	-1.87
10	O.2.am	-7.36	787	H.N	2.7	1.0	0.42
17	H.N	2.91	168	O.2.carbonyl	-4.08	1.0	0.08
10	O.2.am	-8.38	991	H.N	2.84	1.0	0.03
14	H.N	2.07	1006	O.2.am	-7.73	1.0	2.76
19	H.N	3.63	543	O.2.other	-5.5	1.0	-0.8
13	H.N	2.06	1247	O.2.am	-7.21	1.0	2.37
1	N.3.aniline	-3.29	278	H.O	3.66	1.0	1.37
18	H.N	3.52	542	O.2.other	-5.5	1.0	-0.7

Table S13: 1S38 protein-ligand complex

Ligand atom number	Ligand atom type	Ligand AIP value	Protein atom number	Protein atom type	Protein AIP value	f	$\Delta\Delta G$ (kJ/mol)
1	C.ar	-1.82	337	C.ar	-1.52	0.5	-0.63
3	C.ar	-1.7	335	C.ar	-1.42	0.5	-0.81
0	C.ar	-1.98	339	C.ar	-1.53	0.5	-0.48
5	C.ar	-1.13	343	H.soft	0.51	0.5	-1.9
8	C.ar	-2.03	713	H.soft	0.94	0.5	-1.35
1	C.ar	-1.84	1294	H.soft	0.53	0.5	-1.55
16	H.soft	0.52	1299	H.soft	0.81	0.5	-1.59
16	H.soft	0.52	240	O.2.other	-0.1	0.5	-2.04
3	C.ar	-1.53	1290	S.3	-3.5	0.5	1.19
5	C.ar	-0.85	803	H.soft	0.34	0.5	-2.02
7	N.ar.no_lp	0.59	807	H.soft	0.33	0.5	-1.88
8	C.ar	-2.3	1290	S.3	-3.5	0.5	1.85
10	C.ar	-1.84	1293	H.soft	0.51	0.5	-1.55
11	C.ar	-2.53	1293	H.soft	0.51	0.5	-1.06
12	C.ar	-2.05	340	C.ar	-1.32	0.5	-0.58
11	C.ar	-2.03	715	H.N	1.26	0.5	-1.2
21	H.soft	0.78	1103	O.2.am	-7.21	0.5	3.74
21	H.soft	0.78	1146	H.soft	0.49	0.5	-1.64
15	H.soft	0.53	345	H.soft	0.69	1.0	-3.37
0	C.ar	-2.01	1286	C.2	-1.76	0.5	-0.26
13	H.soft	0.84	1327	H.soft	0.88	1.0	-2.5
20	H.soft	0.63	1082	N.pl3.am	-0.38	0.5	-1.98
6	N.pl3.aniline	-0.42	1298	H.soft	0.82	0.5	-1.86
19	H.N	3.84	640	O.2.other	-5.5	1.0	-0.99
9	O.2.am	-6.92	884	H.N	2.7	1.0	0.35
17	H.N	3.23	241	O.2.other	-5.5	1.0	-0.43
9	O.2.am	-7.93	1088	H.N	2.84	1.0	0.02
18	H.N	3.78	639	O.2.other	-5.5	1.0	-0.94

Table S14: 1Z95 protein-ligand complex

Ligand atom number	Ligand atom type	Ligand AIP value	Protein atom number	Protein atom type	Protein AIP value	f	$\Delta\Delta G$ (kJ/mol)
26	F	0.0	959	N.pl3.am	-0.38	0.5	-2.19
26	F	0.0	923	C.2	-1.76	0.5	-1.59
20	C.ar	0.33	927	S.3	-3.5	0.5	-0.05
21	C.ar	0.54	931	H.soft	0.53	0.5	-1.79
22	C.1	-1.68	924	O.2.am	-1.35	0.5	-0.88
22	C.1	-1.13	561	N.pl3.am	-0.26	0.5	-1.85
23	N.1	-2.47	1073	H.soft	0.51	0.5	-1.1
23	N.1	-2.08	931	H.soft	0.53	0.5	-1.4
41	H.soft	1.75	443	N.pl3.am	-0.38	0.5	-0.75
41	H.soft	1.75	415	H.soft	0.33	0.5	-0.45
2	C.ar	0.46	801	H.soft	0.34	0.5	-1.95
21	C.ar	0.71	1363	H.soft	0.68	0.5	-1.56
5	C.ar	-0.23	2173	H.soft	0.84	0.5	-1.83
17	C.ar	0.95	306	H.soft	0.33	0.5	-1.57
1	C.ar	0.15	842	H.soft	0.81	0.5	-1.8
2	C.ar	0.68	842	H.soft	0.81	0.5	-1.47
20	C.ar	0.7	417	H.soft	0.34	0.5	-1.79
8	O.2.sulfone	-2.92	448	H.soft	0.88	0.5	-0.77
8	O.2.sulfone	-2.92	2175	H.soft	0.81	0.5	-0.77
16	N.pl3.am	-0.43	302	H.soft	0.33	0.5	-2.11
0	C.ar	-0.73	841	H.soft	0.82	0.5	-1.84
6	C.ar	0.89	1833	H.soft	0.39	0.5	-1.6
33	H.soft	1.7	1830	H.O	2.85	0.5	3.56
42	H.soft	1.61	559	C.2	-1.35	0.5	-1.04
42	H.soft	1.61	416	H.soft	0.19	0.5	-0.78
0	C.ar	-0.7	935	H.soft	0.82	0.5	-1.85
34	H.soft	1.71	2059	H.soft	0.71	1.0	-0.42
30	H.soft	1.47	827	N.pl3.am	-0.38	0.5	-1.17
30	H.soft	1.47	835	H.soft	1.11	0.5	-0.15
22	C.1	-1.74	1076	H.soft	0.65	0.5	-1.59
31	H.soft	1.54	2277	H.soft	0.33	1.0	-1.61
1	C.ar	-0.01	936	H.soft	0.81	0.5	-1.83
32	H.soft	1.44	2311	H.soft	0.34	1.0	-1.9
37	H.soft	1.28	1826	O.3.alcohol	-5.34	1.0	2.17
5	C.ar	0.5	2308	H.soft	0.23	0.5	-1.98
38	H.soft	1.25	214	H.soft	0.34	1.0	-2.43
23	N.1	-4.96	1201	H.N	2.7	1.0	0.04
35	H.O	3.19	295	O.2.am	-7.21	1.0	-0.96
39	H.N	3.12	295	O.2.am	-7.73	1.0	-0.9

Table S15: 2BR1 protein-ligand complex

Ligand atom number	Ligand atom type	Ligand AIP value	Protein atom number	Protein atom type	Protein AIP value	f	$\Delta\Delta G$ (kJ/mol)
5	C.ar	-0.32	232	C.2	-1.76	0.5	-1.43
6	C.ar	-0.23	230	N.pl3.am	-0.38	0.5	-2.13
28	C.ar	-1.36	202	H.soft	0.33	0.5	-1.83
49	H.soft	0.96	202	H.soft	0.33	1.0	-3.13
10	C.ar	-1.03	447	H.soft	0.33	0.5	-1.97
11	N.pl3.aniline	-0.36	450	H.soft	0.33	0.5	-2.11
35	H.soft	1.14	447	H.soft	0.33	0.5	-1.36
20	C.ar	-0.19	203	H.soft	0.19	0.5	-2.16
21	C.ar	-1.13	203	H.soft	0.19	0.5	-1.95
9	C.ar	-0.22	1377	H.soft	0.33	0.5	-2.12
17	N.ar	-1.75	1086	N.pl3.am	-0.38	0.5	-1.41
18	C.ar	-2.37	1093	H.soft	0.94	0.5	-1.16
19	O.2.one_lp	-2.13	1093	H.soft	0.94	0.5	-1.3
17	N.ar	-1.73	1046	H.soft	1.11	0.5	-1.41
2	C.ar	-0.91	235	H.soft	0.88	0.5	-1.78
43	H.soft	0.92	1089	O.2.am	-1.35	0.5	-1.65
43	H.soft	0.92	1042	C.ar	-1.44	0.5	-1.62
22	C.ar	-1.32	1051	H.soft	0.94	0.5	-1.65
19	O.2.one_lp	-2.04	1051	H.soft	0.94	0.5	-1.35
22	C.ar	-1.0	1162	H.soft	0.76	0.5	-1.83
21	C.ar	-0.83	1162	H.soft	0.76	0.5	-1.87
34	H.soft	1.41	233	O.2.am	-1.35	0.5	-1.26
42	H.soft	0.93	998	O.2.am	-1.71	0.5	-1.51
11	N.pl3.aniline	-0.26	1378	H.soft	0.33	0.5	-2.12
10	C.ar	-0.19	1378	H.soft	0.33	0.5	-2.12
23	C.ar	-0.99	1158	N.pl3.am	-0.38	0.5	-1.88
16	C.ar	-2.52	590	H.soft	0.49	0.5	-1.06
38	H.soft	1.1	648	H.soft	0.33	1.0	-2.82
17	N.ar	-6.39	1096	H.N	2.84	1.0	-0.02

Table S16: 2BRB protein-ligand complex

Ligand atom number	Ligand atom type	Ligand AIP value	Protein atom number	Protein atom type	Protein AIP value	f	$\Delta\Delta G$ (kJ/mol)
30	H.soft	1.0	1050	O.2.am	-1.35	0.5	-1.6
30	H.soft	1.0	1003	C.ar	-1.44	0.5	-1.57
2	C.ar	-0.91	187	H.soft	0.19	0.5	-2.03
3	C.ar	-1.16	187	H.soft	0.19	0.5	-1.93
7	C.ar	-1.01	1012	H.soft	0.94	0.5	-1.72
8	C.ar	-0.05	186	H.soft	0.33	0.5	-2.12
13	C.ar	-1.55	1054	H.soft	0.94	0.5	-1.57
14	O.2.one_lp	-2.13	1050	O.2.am	-7.73	0.5	7.55
15	C.ar	0.28	1300	H.soft	0.33	0.5	-2.04
8	C.ar	0.71	411	H.soft	0.33	0.5	-1.79
16	N.pl3.secondary	0.08	408	H.soft	0.33	0.5	-2.12
39	H.soft	0.86	411	H.soft	0.33	0.5	-1.66
10	C.ar	-0.11	219	H.soft	0.88	0.5	-1.79
2	C.ar	-1.05	1123	H.soft	0.76	0.5	-1.82
7	C.ar	-1.18	1050	O.2.am	-7.21	0.5	6.15
17	C.ar	-0.19	1301	H.soft	0.33	0.5	-2.12
16	N.pl3.secondary	-0.38	1301	H.soft	0.33	0.5	-2.11
6	C.ar	-1.23	1119	N.pl3.am	-0.38	0.5	-1.76
0	C.ar	-1.71	551	H.soft	0.49	0.5	-1.63
25	H.soft	1.06	959	O.2.am	-1.71	0.5	-1.44
25	H.soft	1.06	959	O.2.am	-7.73	0.5	3.61
12	N.ar	-3.17	1007	H.soft	1.11	0.5	-0.59
20	C.ar	0.2	188	H.soft	0.34	0.5	-2.07
15	C.ar	0.45	188	H.soft	0.34	0.5	-1.96
12	N.ar	-3.38	1047	N.pl3.am	-0.38	0.5	0.26
9	C.ar	-0.11	214	N.pl3.am	-0.38	0.5	-2.14
12	N.ar	-6.36	1057	H.N	2.84	1.0	-0.03
24	O.3.alcohol	-7.67	1479	H.N	2.84	1.0	0.01

Table S17: 2C3I protein-ligand complex

Ligand atom number	Ligand atom type	Ligand AIP value	Protein atom number	Protein atom type	Protein AIP value	f	$\Delta\Delta G$ (kJ/mol)
32	H.soft	1.51	1539	C.2	-0.5	0.5	-1.13
5	C.ar	-0.13	1510	H.soft	0.33	0.5	-2.12
7	C.ar	-1.26	1543	H.soft	0.49	0.5	-1.85
9	N.ar.no_lp	-0.11	1510	H.soft	0.33	0.5	-2.12
9	N.ar.no_lp	-0.48	360	H.soft	0.33	0.5	-2.1
8	N.ar	0.09	360	H.soft	0.33	0.5	-2.12
15	C.ar	-1.29	357	H.soft	0.33	0.5	-1.87
19	C.ar	-1.2	1355	H.soft	0.33	0.5	-1.9
18	C.ar	-1.34	1355	H.soft	0.33	0.5	-1.84
11	N.ar	-1.52	1534	N.pl3.am	-0.48	0.5	-1.53
12	C.ar	-2.25	1505	H.soft	0.22	0.5	-1.24
18	C.ar	-1.96	545	H.soft	0.49	0.5	-1.48
31	H.soft	1.62	261	C.ar	-1.53	0.5	-1.01
31	H.soft	1.62	263	C.ar	-1.56	0.5	-1.01
6	C.ar	-0.21	259	C.ar	-1.44	0.5	-1.69
7	C.ar	-0.89	260	C.ar	-1.52	0.5	-1.32
33	H.soft	1.19	901	H.soft	0.33	1.0	-2.6
23	H.soft	0.92	262	C.ar	-1.55	0.5	-1.58
24	H.soft	0.99	138	C.2	-1.76	0.5	-1.46
24	H.soft	0.99	145	H.soft	0.34	0.5	-1.53
14	C.ar	-1.14	1512	H.soft	0.33	0.5	-1.93
36	H.soft	0.97	927	O.2.am	-7.21	0.5	3.32
36	H.soft	0.97	927	O.2.am	-7.73	0.5	3.83
4	N.3.aniline	0.0	264	C.ar	-1.56	0.5	-1.72
17	C.ar	-1.49	546	H.soft	0.49	0.5	-1.75
17	C.ar	-1.34	730	H.soft	0.23	0.5	-1.85
37	H.soft	0.75	978	H.soft	0.34	0.5	-1.75
37	H.soft	0.75	974	H.soft	0.65	0.5	-1.55
12	C.ar	-2.48	614	H.soft	0.33	0.5	-1.07
10	C.ar	-1.97	613	H.soft	0.33	0.5	-1.47
15	C.ar	-1.33	1508	H.soft	0.34	0.5	-1.85
11	N.ar	-7.48	617	H.N	2.7	1.0	0.44

Table S18: 2CET protein-ligand complex

Ligand atom number	Ligand atom type	Ligand AIP value	Protein atom number	Protein atom type	Protein AIP value	f	$\Delta\Delta G$ (kJ/mol)
9	N.ar.no_lp	-0.16	1145	C.ar	-1.32	0.5	-1.78
10	C.ar	-1.13	1143	C.ar	-1.44	0.5	-1.23
11	C.ar	-2.05	1152	H.soft	0.94	0.5	-1.34
25	H.soft	1.22	1146	O.3.any	-3.71	1.0	-0.39
28	H.soft	1.08	1562	C.ar	-1.82	0.5	-1.39
28	H.soft	1.08	1564	C.ar	-1.67	0.5	-1.45
30	H.soft	0.86	429	C.ar	-1.96	0.5	-1.42
30	H.soft	0.86	436	H.soft	0.54	0.5	-1.53
37	H.soft	0.47	625	H.soft	0.33	0.5	-1.95
37	H.soft	0.47	436	H.soft	0.54	0.5	-1.83
23	H.soft	1.7	1144	C.ar	-1.53	0.5	-0.92
23	H.soft	1.7	1977	C.ar	-1.56	0.5	-0.92
38	H.soft	0.74	810	H.soft	1.27	0.5	-0.88
38	H.soft	0.74	804	C.ar	-2.05	0.5	-1.4
20	C.ar	-2.44	1344	H.soft	0.81	0.5	-1.13
24	H.soft	1.37	1974	C.ar	-1.52	0.5	-1.27
10	C.ar	-2.97	1345	H.soft	0.54	0.5	-0.67
19	C.ar	-2.44	1336	C.ar	-1.87	0.5	0.25
33	H.soft	0.67	1338	C.ar	-1.96	0.5	-1.46
36	H.soft	0.46	519	O.2.one_lp	1.93	0.5	-0.03
36	H.soft	0.46	518	C.2	-0.66	0.5	-2.03
12	N.ar	-1.44	520	O.2.carbonyl	-0.84	0.5	-1.4
26	H.soft	1.3	1821	H.soft	1.17	1.0	-0.67
32	H.soft	1.32	1346	H.soft	0.48	1.0	-2.05
12	N.ar	-8.14	527	H.O	3.61	1.0	-2.78
29	H.O	3.06	255	O.2.am	-7.46	1.0	-0.66
4	O.3.alcohol	-1.74	262	H.N	2.7	1.0	0.8
6	O.3.alcohol	-4.28	1820	H.N	2.96	1.0	0.05
31	H.O	2.93	1423	O.2.other	-5.5	1.0	-0.14
8	O.3.alcohol	-5.57	487	H.N	2.62	1.0	0.22
27	H.O	4.43	1771	O.2.other	-5.5	1.0	-1.5
22	H.O	4.07	1771	O.2.other	-5.5	1.0	-1.19
4	O.3.alcohol	-1.89	1572	H.N	2.96	1.0	1.42

Table S19: 2FXS protein-ligand complex

Ligand atom number	Ligand atom type	Ligand AIP value	Protein atom number	Protein atom type	Protein AIP value	f	$\Delta\Delta G$ (kJ/mol)
10	C.ar	-0.45	266	H.soft	0.89	0.5	-1.81
8	C.ar	-0.8	266	H.soft	0.89	0.5	-1.79
4	C.ar	-0.42	757	H.soft	0.81	0.5	-1.87
2	C.2	-0.25	757	H.soft	0.81	0.5	-1.86
34	H.soft	0.8	748	S.3	-3.5	1.0	-0.52
1	O.2.one_lp	0.16	748	S.3	-3.5	0.5	0.04
11	Cl	-1.38	263	N.pl3.am	-0.43	0.5	-1.64
11	Cl	-1.69	1188	C.ar	-1.52	0.5	-0.75
11	Cl	0.0	1194	H.soft	0.57	1.0	-4.06
35	H.soft	0.57	973	H.soft	0.34	1.0	-3.77
2	C.2	0.88	382	H.soft	0.49	0.5	-1.54
4	C.ar	-0.08	382	H.soft	0.49	0.5	-2.05
17	N.pl3.am	-0.24	931	N.pl3.am	-0.43	0.5	-2.11
18	C.ar	-1.8	929	C.2	-1.66	0.5	-0.54
19	C.ar	-3.24	934	H.soft	0.89	0.5	-0.51
20	C.ar	-1.82	932	H.soft	0.65	0.5	-1.55
3	O.2.carbonyl	-2.36	726	C.2	-1.76	0.5	0.08
3	O.2.carbonyl	-2.06	381	H.soft	0.49	0.5	-1.41
8	C.ar	-0.83	1590	H.soft	0.33	0.5	-2.03
10	C.ar	-0.59	1590	H.soft	0.33	0.5	-2.08
11	Cl	-3.09	1200	H.soft	0.68	0.5	-0.6
40	H.soft	1.12	895	N.pl3.am	-0.43	0.5	-1.59
7	C.ar	-0.69	1515	H.soft	0.34	0.5	-2.06
9	O.3.any	-2.57	268	H.N	2.49	1.0	-0.1

Table S20: 2IWX protein-ligand complex

Ligand atom number	Ligand atom type	Ligand AIP value	Protein atom number	Protein atom type	Protein AIP value	f	$\Delta\Delta G$ (kJ/mol)
21	Cl	-0.2	247	N.pl3.am	-0.43	0.5	-2.12
27	H.soft	0.86	247	N.pl3.am	-0.43	0.5	-1.83
1	C.ar	-0.25	775	H.soft	0.81	0.5	-1.86
0	C.2	-0.14	775	H.soft	0.81	0.5	-1.85
4	C.ar	-0.31	250	H.soft	0.89	0.5	-1.8
5	C.ar	0.0	250	H.soft	0.89	0.5	-1.79
37	H.soft	0.97	359	N.pl3.am	-0.38	0.5	-1.74
25	H.soft	0.9	766	S.3	-3.5	1.0	-0.58
36	H.soft	0.74	335	H.soft	0.49	1.0	-3.34
26	H.soft	1.02	991	H.soft	0.34	1.0	-2.99
4	C.ar	-0.57	1485	H.soft	0.33	0.5	-2.09
21	Cl	0.0	1109	C.ar	-1.52	0.5	-1.75
21	Cl	0.0	1115	H.soft	0.57	0.5	-2.03
21	Cl	-1.25	1485	H.soft	0.33	0.5	-1.88
0	C.2	0.6	366	H.soft	0.49	0.5	-1.78
2	C.ar	-0.62	366	H.soft	0.49	0.5	-2.03
3	C.ar	-0.58	1410	H.soft	0.34	0.5	-2.08
41	H.soft	1.04	949	N.pl3.am	-0.43	0.5	-1.67
32	H.soft	0.56	245	C.2	-1.66	0.5	-1.65
20	O.3.any	-2.47	252	H.N	2.49	1.0	-0.08
23	O.2.one_lp	-0.54	954	H.N	2.49	1.0	1.04
44	H.O	4.52	141	O.2.am	-7.73	1.0	-5.3

Table S21: 2POG protein-ligand complex

Ligand atom number	Ligand atom type	Ligand AIP value	Protein atom number	Protein atom type	Protein AIP value	f	$\Delta\Delta G$ (kJ/mol)
20	C.ar	-1.53	386	H.soft	0.49	0.5	-1.73
15	C.ar	-2.12	925	H.soft	0.33	0.5	-1.36
16	C.ar	-1.95	920	H.soft	0.34	0.5	-1.48
28	H.soft	0.42	1922	H.soft	0.33	1.0	-3.96
16	C.ar	-1.78	789	H.soft	0.33	0.5	-1.6
17	C.ar	-1.43	789	H.soft	0.33	0.5	-1.8
19	C.ar	-1.48	794	H.soft	0.33	0.5	-1.77
0	C.ar	-1.56	251	H.soft	0.33	0.5	-1.73
6	C.ar	-1.72	251	H.soft	0.33	0.5	-1.63
25	H.soft	0.67	246	H.soft	0.33	0.5	-1.82
25	H.soft	0.67	1145	C.ar	-1.55	0.5	-1.68
26	H.soft	0.7	238	C.2	-1.76	0.5	-1.57
26	H.soft	0.7	246	H.soft	0.33	0.5	-1.8
38	H.soft	1.06	239	O.2.am	-1.35	0.5	-1.56
1	C.ar	-1.77	1370	H.soft	0.81	0.5	-1.53
3	C.ar	-2.06	1367	H.soft	0.65	0.5	-1.4
23	H.soft	0.92	1474	H.soft	0.22	0.5	-1.66
23	H.soft	0.92	1517	H.soft	0.68	0.5	-1.36
31	H.soft	0.54	679	H.soft	0.33	0.5	-1.91
31	H.soft	0.54	617	C.ar	-1.96	0.5	-1.48
3	C.ar	-1.87	1479	H.soft	0.33	0.5	-1.54
21	H.O	3.96	1870	N.ar.no_lp	-0.75	0.5	3.36
22	H.soft	1.1	1872	C.ar	-2.05	0.5	-1.28
4	C.ar	-2.0	1481	H.soft	0.33	0.5	-1.45
37	H.soft	1.24	352	H.soft	0.19	0.5	-1.32
37	H.soft	1.24	379	N.pl3.am	-0.38	0.5	-1.46
19	C.ar	-1.75	353	H.soft	0.34	0.5	-1.62
32	H.soft	0.41	680	H.soft	0.33	1.0	-3.97
20	C.ar	-1.75	1155	H.soft	0.68	0.5	-1.58
14	C.ar	-1.82	1155	H.soft	0.68	0.5	-1.54
4	C.ar	-2.09	1518	H.soft	0.71	0.5	-1.38
18	O.3.any	-3.59	1003	H.N	2.7	1.0	0.03
36	H.O	3.96	496	O.2.other	-5.5	1.0	-1.09
2	O.3.any	-3.65	1879	H.N	3.44	1.0	0.72

Table S22: 2P15 protein-ligand complex

Ligand atom number	Ligand atom type	Ligand AIP value	Protein atom number	Protein atom type	Protein AIP value	f	$\Delta\Delta G$ (kJ/mol)
7	C.ar	-1.95	899	H.soft	0.33	0.5	-1.48
8	C.ar	-1.75	894	H.soft	0.33	0.5	-1.62
10	C.ar	-1.9	894	H.soft	0.33	0.5	-1.52
6	C.ar	-1.99	491	H.soft	0.49	0.5	-1.46
37	H.soft	0.61	351	H.soft	0.33	0.5	-1.86
37	H.soft	0.61	343	C.2	-1.76	0.5	-1.59
40	H.soft	0.88	344	O.2.am	-1.35	0.5	-1.67
26	C.ar	-0.71	1766	H.soft	0.68	0.5	-1.93
23	C.ar	-0.71	358	H.soft	0.34	0.5	-2.06
55	H.soft	1.09	271	S.3	0.0	0.5	-1.59
29	F	0.0	1754	C.ar	-1.52	0.5	-1.75
7	C.ar	-2.17	458	H.soft	0.34	0.5	-1.32
8	C.ar	-2.06	1234	H.soft	0.68	0.5	-1.4
34	H.soft	0.58	2055	O.2.am	-1.35	0.5	-1.79
23	C.ar	-0.71	1611	H.soft	0.65	0.5	-1.94
24	C.ar	-0.71	1611	H.soft	0.65	0.5	-1.94
57	H.soft	1.28	1520	H.soft	0.33	1.0	-2.37
33	H.soft	0.61	2205	H.soft	0.33	1.0	-3.73
25	C.ar	-0.73	357	H.soft	0.33	0.5	-2.06
25	C.ar	-0.72	1615	H.soft	0.81	0.5	-1.85
5	C.ar	-2.03	1233	H.soft	0.7	0.5	-1.41
10	C.ar	-2.12	1030	H.soft	0.33	0.5	-1.36
11	C.ar	-2.07	1030	H.soft	0.33	0.5	-1.4
43	H.soft	0.72	1025	H.soft	0.34	1.0	-3.55
30	F	0.0	1748	N.pl3.am	-0.38	0.5	-2.19
30	F	0.0	1719	H.soft	0.22	0.5	-2.19
51	H.soft	0.54	782	H.soft	0.34	0.5	-1.9
51	H.soft	0.54	935	H.soft	0.84	0.5	-1.55
41	H.soft	1.05	457	H.soft	0.19	0.5	-1.54
41	H.soft	1.05	484	N.pl3.am	-0.38	0.5	-1.66
20	C.2	-0.57	2155	C.ar	-2.05	0.5	-1.06
52	H.soft	0.65	2153	N.ar.no_lp	-0.75	0.5	-1.94
39	H.soft	0.41	356	H.soft	0.33	0.5	-1.98
39	H.soft	0.41	1224	C.ar	-1.55	0.5	-1.73
58	H.soft	1.23	1523	H.soft	0.33	1.0	-2.5
45	H.soft	0.57	922	N.pl3.am	-0.38	0.5	-2.01
45	H.soft	0.57	934	H.soft	0.65	0.5	-1.69
48	H.soft	0.47	785	H.soft	0.33	1.0	-3.9
44	H.soft	0.56	930	H.soft	1.11	1.0	-2.48
53	H.soft	0.64	2159	H.soft	0.49	1.0	-3.5
27	C.ar	-0.56	1609	H.soft	0.51	0.5	-2.03
22	C.ar	-0.58	1609	H.soft	0.51	0.5	-2.03
24	C.ar	-0.72	239	H.soft	0.65	0.5	-1.94
50	H.soft	0.58	1726	H.soft	0.33	1.0	-3.77
59	H.soft	1.22	1341	H.soft	0.33	1.0	-2.52
[illegible]	[illegible]	[illegible]	[illegible]	[illegible]	[illegible]	[illegible]	[illegible]

Table S23: 2QE4 protein-ligand complex

Ligand atom number	Ligand atom type	Ligand AIP value	Protein atom number	Protein atom type	Protein AIP value	f	$\Delta\Delta G$ (kJ/mol)
19	C.ar	-1.45	416	H.soft	0.49	0.5	-1.77
26	H.soft	0.74	269	O.2.am	-1.35	0.5	-1.74
1	C.ar	-1.34	1438	H.soft	0.81	0.5	-1.71
2	C.ar	-1.32	1438	H.soft	0.81	0.5	-1.72
12	C.ar	-1.59	1990	H.soft	0.33	0.5	-1.71
18	C.ar	-1.78	1185	H.soft	0.68	0.5	-1.56
19	C.ar	-1.76	280	H.soft	0.33	0.5	-1.61
15	C.ar	-1.84	819	H.soft	0.33	0.5	-1.56
16	C.ar	-1.44	819	H.soft	0.33	0.5	-1.79
18	C.ar	-1.41	824	H.soft	0.33	0.5	-1.81
40	H.soft	0.73	860	H.soft	0.84	0.5	-1.4
40	H.soft	0.73	710	H.soft	0.33	0.5	-1.77
14	C.ar	-2.06	1184	H.soft	0.7	0.5	-1.4
15	C.ar	-2.06	955	H.soft	0.33	0.5	-1.4
41	H.soft	0.75	1549	H.soft	0.33	1.0	-3.52
33	H.soft	0.7	315	H.soft	0.36	0.5	-1.78
33	H.soft	0.7	304	N.pl3.am	-0.38	0.5	-1.94
25	H.soft	0.71	276	H.soft	0.33	0.5	-1.79
25	H.soft	0.71	281	H.soft	0.33	0.5	-1.79
24	H.soft	1.22	1937	C.ar	-1.35	0.5	-1.44
45	H.O	3.92	1938	N.ar.no_lp	-0.75	0.5	3.28
32	H.soft	0.55	311	H.soft	1.11	1.0	-2.5
29	H.soft	0.44	1989	H.soft	0.33	1.0	-3.94
43	H.soft	0.63	1542	H.soft	0.22	1.0	-3.81
0	C.ar	-1.82	1984	H.soft	0.34	0.5	-1.57
36	H.soft	0.81	855	H.soft	1.11	1.0	-2.04
37	H.O	3.91	526	O.2.other	-5.5	1.0	-1.05
17	O.3.any	-3.7	1033	H.N	2.7	1.0	0.02
23	O.3.any	-3.96	1947	H.N	3.44	1.0	0.45

Table S24: 2V00 protein-ligand complex

Ligand atom number	Ligand atom type	Ligand AIP value	Protein atom number	Protein atom type	Protein AIP value	f	$\Delta\Delta G$ (kJ/mol)
4	C.ar	-1.38	567	C.ar	-1.45	0.5	-1.04
27	H.soft	0.73	566	C.ar	-1.42	0.5	-1.72
27	H.soft	0.73	568	C.ar	-1.52	0.5	-1.67
25	H.soft	0.88	569	C.ar	-1.44	0.5	-1.64
6	C.ar	-1.8	830	H.soft	0.7	0.5	-1.55
13	C.ar	-1.64	821	C.ar	-1.55	0.5	-0.76
3	C.ar	-2.4	575	H.soft	0.5	0.5	-1.16
2	C.ar	-2.69	575	H.soft	0.5	0.5	-0.92
10	C.ar	-1.82	692	H.O	2.88	0.5	0.63
5	N.ar	0.26	309	O.2.other	-0.1	0.5	-2.14
17	H.soft	0.74	636	C.2	-0.5	0.5	-1.91
18	H.soft	0.97	996	H.soft	0.34	1.0	-3.09
1	C.ar	-0.0	1297	C.2	-1.76	0.5	-1.59
28	H.soft	0.74	570	C.ar	-1.53	0.5	-1.67
28	H.soft	0.74	829	H.soft	0.69	0.5	-1.52
23	H.N	3.33	308	O.2.other	-5.5	1.0	-0.53
16	H.N	3.49	1318	O.3.alcohol	-5.34	1.0	-0.57
24	H.N	2.7	1237	O.2.other	-5.5	1.0	0.11

Table S25: 2VVN protein-ligand complex

Ligand atom number	Ligand atom type	Ligand AIP value	Protein atom number	Protein atom type	Protein AIP value	f	$\Delta\Delta G$ (kJ/mol)
14	S.3	-1.18	824	C.ar	-1.44	0.5	-1.19
14	S.3	-1.18	1211	H.soft	0.33	0.5	-1.91
31	H.soft	0.93	822	C.ar	-1.45	0.5	-1.61
31	H.soft	0.93	821	C.ar	-1.42	0.5	-1.62
30	H.soft	0.95	1368	C.ar	-1.57	0.5	-1.56
30	H.soft	0.95	1370	C.ar	-1.82	0.5	-1.45
6	C.2	-1.87	1372	C.ar	-1.67	0.5	-0.47
8	N.2	-3.04	1374	C.ar	-1.87	0.5	0.91
12	N.3.secondary	-0.22	1374	C.ar	-1.87	0.5	-1.38
14	S.3	-1.15	1371	N.ar.no_lp	-1.17	0.5	-1.4
14	S.3	0.0	1494	H.N	2.62	0.5	1.02
14	S.3	-1.15	1494	H.N	2.62	0.5	0.5
14	S.3	0.0	1490	N.pl3.am	-0.43	0.5	-2.18
14	S.3	-0.3	1490	N.pl3.am	-0.43	0.5	-2.1
24	H.soft	1.03	1375	C.ar	-1.99	0.5	-1.34
24	H.soft	1.03	1373	C.ar	-1.98	0.5	-1.34
25	H.soft	1.1	593	H.soft	0.49	0.5	-1.3
25	H.soft	1.1	823	C.ar	-1.52	0.5	-1.48
20	H.soft	0.78	1489	O.2.am	-1.23	0.5	-1.76
18	H.soft	0.8	1835	N.pl3.am	-0.43	0.5	-1.87
18	H.soft	0.8	1382	H.soft	0.81	0.5	-1.36
6	C.2	-0.96	826	C.ar	-1.32	0.5	-1.42
17	H.soft	0.91	221	H.soft	0.85	1.0	-2.42
22	H.soft	0.57	1488	C.2	-1.66	0.5	-1.65
19	H.soft	0.99	220	H.soft	0.69	1.0	-2.55
27	H.O	3.49	1663	O.2.other	-5.5	1.0	-0.67
26	H.O	3.04	141	O.2.am	-7.21	1.0	-0.55
10	O.3.alcohol	-3.18	1839	H.N	2.62	1.0	0.0
28	H.N	2.05	590	O.2.other	-5.5	1.0	0.96
8	N.2	-6.7	341	H.N	2.7	1.0	0.31
9	O.3.alcohol	-5.03	340	H.N	2.7	1.0	0.05

Table S26: 2W4X protein-ligand complex

Ligand atom number	Ligand atom type	Ligand AIP value	Protein atom number	Protein atom type	Protein AIP value	f	$\Delta\Delta G$ (kJ/mol)
12	C.2	0.34	612	C.ar	-1.44	0.5	-1.79
14	N.pl3.am	2.12	614	C.ar	-1.32	0.5	-0.36
31	H.soft	1.25	610	C.ar	-1.45	0.5	-1.39
31	H.soft	1.25	862	H.soft	0.39	0.5	-1.18
16	N.2	-1.06	453	C.2	-0.5	0.5	-1.8
32	H.soft	1.2	1012	C.ar	-1.82	0.5	-1.32
32	H.soft	1.2	1014	C.ar	-1.67	0.5	-1.37
12	C.2	-0.37	1016	C.ar	-1.87	0.5	-1.32
14	N.pl3.am	1.44	1018	C.ar	-1.96	0.5	-1.09
30	H.soft	1.29	521	S.3	0.0	0.5	-1.35
30	H.soft	1.29	521	S.3	-3.32	0.5	-0.48
22	H.soft	0.76	1130	C.2	-1.66	0.5	-1.6
24	H.soft	0.97	980	H.soft	0.34	1.0	-3.09
26	H.soft	0.65	1132	N.pl3.am	-0.43	0.5	-1.97
26	H.soft	0.65	943	H.soft	0.33	0.5	-1.84
28	H.soft	1.01	228	H.soft	0.85	1.0	-2.2
21	H.O	4.28	1276	O.2.other	-5.5	1.0	-1.37
9	O.3.alcohol	-5.11	623	H.O	3.66	1.0	-0.5
18	H.O	4.26	148	O.2.am	-7.21	1.0	-3.77
13	O.2.am	-4.4	1136	H.N	2.62	1.0	-0.0
3	O.3.alcohol	-3.4	1452	H.N	2.62	1.0	-0.03
25	H.O	3.03	1275	O.2.other	-5.5	1.0	-0.24

Table S27: 2WBG protein-ligand complex

Ligand atom number	Ligand atom type	Ligand AIP value	Protein atom number	Protein atom type	Protein AIP value	f	$\Delta\Delta G$ (kJ/mol)
29	H.soft	0.86	432	C.ar	-1.96	0.5	-1.42
33	H.soft	0.29	1445	C.ar	-1.99	0.5	-1.45
33	H.soft	0.29	1163	C.ar	-1.42	0.5	-1.81
4	O.2.one_lp	-1.91	1453	H.soft	0.54	0.5	-1.51
4	O.2.one_lp	-2.55	1167	C.ar	-1.53	0.5	0.09
12	C.2	-2.06	1168	C.ar	-1.32	0.5	-0.57
13	N.2	-1.64	1166	C.ar	-1.44	0.5	-0.85
34	H.soft	0.18	1164	C.ar	-1.45	0.5	-1.79
37	H.soft	0.36	1440	C.ar	-1.82	0.5	-1.57
37	H.soft	0.36	1443	C.ar	-1.98	0.5	-1.46
41	H.soft	0.39	1438	C.ar	-1.57	0.5	-1.72
41	H.soft	0.39	1439	C.ar	-1.53	0.5	-1.74
31	H.soft	0.93	1570	O.2.other	-0.1	0.5	-1.74
24	H.soft	1.12	1169	O.3.any	-3.71	0.5	-0.17
24	H.soft	1.12	1759	C.ar	-1.57	0.5	-1.45
27	H.soft	1.24	1570	O.2.other	-0.1	0.5	-1.41
27	H.soft	1.24	1761	C.ar	-1.82	0.5	-1.29
36	H.soft	0.25	1446	C.ar	-1.96	0.5	-1.47
40	H.soft	0.38	1444	C.ar	-1.87	0.5	-1.54
44	H.soft	0.42	1268	H.soft	1.27	1.0	-2.27
45	H.soft	0.42	1262	C.ar	-2.05	0.5	-1.42
26	H.soft	1.46	1760	C.ar	-1.53	0.5	-1.18
26	H.soft	1.46	2114	C.ar	-1.56	0.5	-1.18
23	H.soft	1.07	2020	H.soft	1.17	1.0	-1.3
22	H.O	4.06	1970	O.2.other	-5.5	1.0	-1.18
28	H.O	3.07	258	O.2.am	-7.46	1.0	-0.69
6	O.3.alcohol	-4.11	2019	H.N	2.96	1.0	0.1
0	O.3.alcohol	-2.0	265	H.N	2.7	1.0	0.62
32	H.O	3.07	522	O.2.other	-5.5	1.0	-0.28
30	H.O	3.47	1571	O.2.other	-5.5	1.0	-0.66
8	O.3.alcohol	-4.59	490	H.N	2.62	1.0	0.02

Table S28: 2WCA protein-ligand complex

Ligand atom number	Ligand atom type	Ligand AIP value	Protein atom number	Protein atom type	Protein AIP value	f	$\Delta\Delta G$ (kJ/mol)
3	C.2	-0.5	1187	C.ar	-1.87	0.5	-1.25
29	H.soft	0.77	672	C.ar	-1.42	0.5	-1.7
29	H.soft	0.77	674	C.ar	-1.52	0.5	-1.66
18	C.2	-3.49	775	H.soft	0.54	0.5	-0.16
20	N.pl3.am	-1.25	775	H.soft	0.54	0.5	-1.84
21	C.ar	-2.16	768	C.ar	-1.96	0.5	0.05
30	H.soft	0.58	1185	C.ar	-1.67	0.5	-1.64
30	H.soft	0.58	1184	N.ar.no_lp	-1.17	0.5	-1.86
31	H.soft	0.58	925	H.soft	0.39	0.5	-1.85
31	H.soft	0.58	675	C.ar	-1.44	0.5	-1.75
32	H.soft	1.16	677	C.ar	-1.32	0.5	-1.49
32	H.soft	1.16	676	C.ar	-1.53	0.5	-1.44
40	H.soft	0.92	1302	O.2.am	-1.23	0.5	-1.68
33	H.soft	1.17	1189	C.ar	-1.96	0.5	-1.28
17	O.2.one_lp	-4.1	1025	H.soft	0.33	0.5	0.64
28	H.soft	0.72	1188	C.ar	-1.99	0.5	-1.44
28	H.soft	0.72	1186	C.ar	-1.98	0.5	-1.44
35	H.soft	1.47	270	H.soft	0.85	1.0	-0.93
27	H.soft	0.74	924	H.soft	0.36	1.0	-3.5
36	H.soft	1.06	1648	N.pl3.am	-0.43	0.5	-1.65
42	H.soft	1.4	1061	H.soft	0.34	1.0	-2.02
39	H.O	4.41	1476	O.2.other	-5.5	1.0	-1.48
4	O.2.am	-6.95	1307	H.N	2.62	1.0	0.56
37	H.O	3.53	190	O.2.am	-7.21	1.0	-1.87
10	O.3.alcohol	-1.75	1652	H.N	2.62	1.0	0.56
43	H.O	3.78	1475	O.2.other	-5.5	1.0	-0.94

Table S29: 2WN9 protein-ligand complex

Ligand atom number	Ligand atom type	Ligand AIP value	Protein atom number	Protein atom type	Protein AIP value	f	$\Delta\Delta G$ (kJ/mol)
35	H.soft	0.6	406	C.ar	-1.98	0.5	-1.46
35	H.soft	0.6	190	C.ar	-1.44	0.5	-1.75
2	C.ar	-0.65	1075	H.soft	0.7	0.5	-1.93
18	N.ar	-4.19	467	H.soft	0.33	0.5	0.75
14	N.2	-3.61	403	C.ar	-1.82	0.5	1.56
15	C.2	-1.85	405	C.ar	-1.67	0.5	-0.49
17	C.ar	-2.6	404	N.ar.no_lp	-1.17	0.5	-0.13
18	N.ar	-3.61	1528	H.soft	0.22	0.5	0.13
36	H.soft	0.57	402	C.ar	-1.53	0.5	-1.71
36	H.soft	0.57	401	C.ar	-1.57	0.5	-1.7
6	C.ar	-1.17	1074	H.soft	0.94	0.5	-1.69
0	C.2	-1.32	1063	C.ar	-1.45	0.5	-1.09
10	C.2	-1.28	1065	C.ar	-1.44	0.5	-1.12
32	H.soft	0.64	189	C.ar	-1.52	0.5	-1.7
32	H.soft	0.64	187	C.ar	-1.42	0.5	-1.75
23	H.soft	1.32	1158	H.soft	0.3	0.5	-1.15
23	H.soft	1.32	1531	H.soft	0.34	0.5	-1.12
8	C.ar	-0.73	907	H.soft	0.39	0.5	-2.04
5	C.ar	-0.59	907	H.soft	0.39	0.5	-2.07
33	H.soft	0.59	409	C.ar	-1.96	0.5	-1.47
33	H.soft	0.59	408	C.ar	-1.99	0.5	-1.45
7	C.ar	-1.27	624	H.soft	0.85	0.5	-1.71
24	H.soft	1.26	1070	H.soft	0.51	1.0	-2.17
19	C.ar	-2.34	1533	H.soft	0.33	0.5	-1.19
25	H.soft	1.17	1156	N.pl3.am	-0.26	0.5	-1.52
29	H.O	4.36	1616	O.2.other	-5.5	1.0	-1.44

Table S30: 2XDL protein-ligand complex

Ligand atom number	Ligand atom type	Ligand AIP value	Protein atom number	Protein atom type	Protein AIP value	f	$\Delta\Delta G$ (kJ/mol)
25	H.soft	0.93	756	O.2.am	-1.35	0.5	-1.64
25	H.soft	0.93	783	H.soft	0.65	0.5	-1.37
11	C.2	-2.07	392	H.soft	0.49	0.5	-1.4
14	N.pl3.am	-1.05	392	H.soft	0.49	0.5	-1.93
21	H.soft	0.85	385	N.pl3.am	-0.38	0.5	-1.84
5	C.ar	-1.93	1470	H.soft	0.34	0.5	-1.5
5	C.ar	-1.41	299	N.pl3.am	-0.38	0.5	-1.65
3	C.ar	-2.96	786	H.soft	0.81	0.5	-0.73
1	C.ar	-0.61	273	H.soft	0.89	0.5	-1.8
2	C.ar	-0.65	273	H.soft	0.89	0.5	-1.8
30	H.soft	1.27	1109	C.ar	-1.44	0.5	-1.37
16	H.soft	1.31	777	S.3	-3.5	1.0	-0.72
32	H.soft	1.47	958	H.soft	0.33	0.5	-0.91
28	H.soft	0.76	991	H.soft	0.88	1.0	-2.66
4	C.ar	-3.61	1468	H.soft	0.38	0.5	0.05
13	O.3.any	-0.87	275	H.N	2.49	1.0	0.76
12	O.2.am	-8.6	1469	H.O	2.85	1.0	-0.0

Table S31: 2YKI protein-ligand complex

Ligand atom number	Ligand atom type	Ligand AIP value	Protein atom number	Protein atom type	Protein AIP value	f	$\Delta\Delta G$ (kJ/mol)
14	C.ar	-0.86	2046	C.ar	-1.82	0.5	-1.09
15	C.ar	-0.86	2048	C.ar	-1.67	0.5	-1.22
2	C.ar	-2.08	2174	C.ar	-1.56	0.5	-0.37
35	H.soft	1.23	1194	H.soft	1.11	1.0	-1.03
32	C.ar	-1.58	579	N.pl3.am	-0.38	0.5	-1.53
22	C.ar	-0.68	1689	C.ar	-1.56	0.5	-1.4
25	C.ar	-2.35	1687	C.ar	-1.53	0.5	-0.12
45	H.soft	1.0	1685	C.ar	-1.44	0.5	-1.57
45	H.soft	1.0	1686	C.ar	-1.52	0.5	-1.55
38	H.N	2.73	985	S.3	-3.5	1.0	0.08
19	C.ar	-0.54	985	S.3	-3.5	0.5	0.51
6	C.ar	-0.05	1306	H.soft	0.33	0.5	-2.12
22	C.ar	0.14	1311	H.soft	0.33	0.5	-2.1
23	C.ar	0.02	1311	H.soft	0.33	0.5	-2.14
24	C.ar	-1.03	1310	H.soft	0.33	0.5	-1.97
26	C.ar	-1.43	1306	H.soft	0.33	0.5	-1.8
3	N.ar.no_lp	0.57	2047	N.ar.no_lp	-1.17	0.5	-1.86
42	H.soft	1.61	2045	C.ar	-1.53	0.5	-1.03
26	C.ar	-3.62	1745	H.soft	0.85	0.5	-0.16
5	N.ar	-1.93	1428	H.soft	0.49	0.5	-1.5
46	H.soft	0.68	1299	O.2.am	-1.35	0.5	-1.76
13	C.ar	-0.81	1690	C.ar	-1.56	0.5	-1.33
30	C.ar	-1.56	553	H.soft	0.89	0.5	-1.59
33	C.ar	-2.56	553	H.soft	0.89	0.5	-1.04
13	C.ar	-0.15	985	S.3	0.0	0.5	-2.21
47	H.soft	0.91	1421	N.pl3.am	-0.38	0.5	-1.79
28	C.ar	-1.84	1636	H.soft	0.65	0.5	-1.54
2	C.ar	-2.03	1334	N.pl3.am	-0.38	0.5	-1.18
10	C.ar	-0.37	1298	C.2	-1.76	0.5	-1.4
3	N.ar.no_lp	0.8	1305	H.soft	0.34	0.5	-1.71
4	C.ar	-1.37	230	H.soft	0.7	0.5	-1.75
8	C.ar	-1.68	230	H.soft	0.7	0.5	-1.61
11	N.ar.no_lp	-0.43	2303	H.soft	0.38	0.5	-2.09
9	N.ar	-2.77	672	H.soft	0.49	0.5	-0.85
11	N.ar.no_lp	-0.58	585	H.soft	0.64	0.5	-1.97
14	C.ar	-0.21	985	S.3	-3.5	0.5	0.29
15	C.ar	-0.16	1156	H.soft	1.11	0.5	-1.57
37	H.soft	0.96	313	H.soft	0.23	1.0	-3.24
0	C.ar	-2.24	1338	H.soft	0.76	0.5	-1.27
4	C.ar	-1.3	1429	H.soft	0.49	0.5	-1.84
9	N.ar	-2.95	994	H.soft	0.81	0.5	-0.74
20	C.ar	-1.66	991	H.soft	0.65	0.5	-1.63
31	C.ar	-0.95	994	H.soft	0.81	0.5	-1.82
27	C.ar	-1.95	1640	H.soft	0.33	0.5	-1.48
41	H.soft	1.26	2044	C.ar	-1.57	0.5	-1.35
[illegible]	[illegible]	[illegible]	[illegible]	[illegible]	[illegible]	[illegible]	[illegible]

Table S32: 3AO4 protein-ligand complex

Ligand atom number	Ligand atom type	Ligand AIP value	Protein atom number	Protein atom type	Protein AIP value	f	$\Delta\Delta G$ (kJ/mol)
9	C.ar	-3.12	1220	H.soft	0.34	0.5	-0.46
1	C.ar	-2.38	1220	H.soft	0.34	0.5	-1.15
11	C.ar	-2.51	723	H.soft	0.34	0.5	-1.04
12	N.ar.no_lp	-1.9	723	H.soft	0.34	0.5	-1.52
10	C.ar	-2.59	720	H.soft	1.11	0.5	-0.98
4	C.ar	-1.79	1152	C.2	-1.76	0.5	-0.46
5	C.ar	-1.93	1210	N.pl3.am	-0.59	0.5	-1.15
4	C.ar	-1.82	698	H.soft	0.88	0.5	-1.48
5	C.ar	-1.92	698	H.soft	0.88	0.5	-1.43
2	C.ar	-2.35	1218	H.soft	0.34	0.5	-1.18
3	C.ar	-2.25	1164	H.soft	1.11	0.5	-1.17
1	C.ar	-2.31	711	N.pl3.am	-0.48	0.5	-0.87
2	C.ar	-2.3	695	C.2	-1.76	0.5	0.02
17	H.soft	0.88	696	O.2.am	-1.35	0.5	-1.67
17	H.soft	0.88	854	H.soft	0.33	0.5	-1.64
0	C.ar	-2.69	1223	H.soft	0.47	0.5	-0.91
19	H.soft	1.12	1059	O.2.am	-1.35	0.5	-1.52
19	H.soft	1.12	1153	O.2.am	-1.35	0.5	-1.52
8	O.3.any	-2.64	1099	C.2	-2.23	0.5	0.79
8	O.3.any	-2.64	1100	O.2.am	-1.71	0.5	0.33
8	O.3.any	-2.64	81	H.soft	0.85	1.0	-1.97
20	H.soft	1.24	419	C.2	-1.76	0.5	-1.31
20	H.soft	1.24	459	N.ar.no_lp	-0.75	0.5	-1.48
11	C.ar	-1.71	1401	H.soft	0.33	0.5	-1.64
9	C.ar	-2.97	724	H.soft	0.34	0.5	-0.61
3	C.ar	-2.24	697	H.soft	0.76	0.5	-1.27
10	C.ar	-2.3	1402	H.soft	0.33	0.5	-1.22
22	H.N	2.74	1389	O.2.am	-7.21	1.0	0.28

Table S33: 3ARU protein-ligand complex

Ligand atom number	Ligand atom type	Ligand AIP value	Protein atom number	Protein atom type	Protein AIP value	f	$\Delta\Delta G$ (kJ/mol)
7	N.ar.no_lp	0.46	880	C.ar	-1.87	0.5	-1.54
8	C.ar	-0.94	878	C.ar	-1.67	0.5	-1.17
10	C.ar	0.19	876	C.ar	-1.82	0.5	-1.56
11	N.ar.no_lp	0.38	874	C.ar	-1.57	0.5	-1.72
15	C.ar	-0.88	879	C.ar	-1.98	0.5	-0.94
16	N.ar.no_lp	0.26	881	C.ar	-1.99	0.5	-1.45
18	C.ar	-0.95	882	C.ar	-1.96	0.5	-0.92
32	H.soft	1.22	875	C.ar	-1.53	0.5	-1.39
1	C.2	-1.0	1038	C.ar	-1.53	0.5	-1.24
37	H.soft	1.03	1215	N.pl3.primary	-0.1	0.5	-1.65
22	H.soft	0.99	1034	C.ar	-1.42	0.5	-1.59
22	H.soft	0.99	1036	C.ar	-1.52	0.5	-1.55
26	H.soft	0.5	187	C.ar	-1.56	0.5	-1.71
26	H.soft	0.5	512	H.soft	0.35	0.5	-1.92
29	H.soft	0.9	701	O.2.am	-7.73	0.5	4.01
29	H.soft	0.9	708	H.soft	0.49	0.5	-1.52
28	H.soft	0.6	193	H.soft	0.69	1.0	-3.27
13	C.ar	-1.29	885	H.soft	0.46	0.5	-1.85
21	H.soft	0.94	392	N.pl3.am	-0.38	0.5	-1.76
24	H.soft	1.0	701	O.2.am	-1.71	0.5	-1.48
20	H.soft	1.15	750	H.soft	0.68	1.0	-2.18
2	O.2.carbonyl	-5.8	398	H.N	2.84	1.0	-0.04
2	O.2.carbonyl	-5.9	514	H.N	2.7	1.0	0.17

Table S34: 3ARV protein-ligand complex

Ligand atom number	Ligand atom type	Ligand AIP value	Protein atom number	Protein atom type	Protein AIP value	f	$\Delta\Delta G$ (kJ/mol)
0	N.pl3.aniline	-1.69	319	C.ar	-1.87	0.5	-0.45
10	C.ar	-1.79	321	C.ar	-1.96	0.5	-0.28
16	C.ar	-1.57	317	C.ar	-1.67	0.5	-0.72
17	C.ar	-1.88	315	C.ar	-1.82	0.5	-0.33
18	C.ar	-2.14	320	C.ar	-1.99	0.5	0.06
32	H.soft	0.64	316	N.ar.no_lp	-1.17	0.5	-1.84
3	C.ar	-1.33	894	C.ar	-1.53	0.5	-1.02
5	C.ar	-1.05	893	C.ar	-1.57	0.5	-1.18
7	C.ar	-1.59	897	C.ar	-1.67	0.5	-0.71
9	C.ar	-1.88	895	C.ar	-1.82	0.5	-0.33
10	C.ar	-1.41	898	C.ar	-1.98	0.5	-0.58
37	H.soft	0.87	904	H.soft	0.46	1.0	-3.15
12	C.ar	-2.35	727	H.soft	0.49	0.5	-1.2
13	C.ar	-2.36	722	C.2	-0.5	0.5	-0.81
18	C.ar	-2.11	900	C.ar	-1.99	0.5	0.03
29	H.soft	0.73	901	C.ar	-1.96	0.5	-1.45
21	C.ar	-1.41	313	C.ar	-1.57	0.5	-0.93
22	C.ar	-1.23	314	C.ar	-1.53	0.5	-1.09
19	C.ar	-1.84	1098	N.pl3.primary	-0.1	0.5	-1.46
20	C.ar	-1.9	323	H.soft	0.49	0.5	-1.52

Table S35: 3ARY protein-ligand complex

Ligand atom number	Ligand atom type	Ligand AIP value	Protein atom number	Protein atom type	Protein AIP value	f	$\Delta\Delta G$ (kJ/mol)
3	C.ar	-0.08	1126	N.pl3.primary	-0.1	0.5	-2.18
0	C.ar	-0.1	341	C.ar	-1.57	0.5	-1.65
1	C.ar	-0.08	342	C.ar	-1.53	0.5	-1.68
2	C.ar	0.06	345	C.ar	-1.67	0.5	-1.65
3	C.ar	-0.05	343	C.ar	-1.82	0.5	-1.49
4	C.ar	-0.38	346	C.ar	-1.98	0.5	-1.22
5	C.ar	-0.38	351	H.soft	0.49	0.5	-2.05
6	C.ar	0.43	344	N.ar.no_lp	-1.17	0.5	-1.9
7	C.ar	-0.34	347	C.ar	-1.87	0.5	-1.33
9	C.2	-1.37	349	C.ar	-1.96	0.5	-0.62
7	C.ar	-0.76	928	C.ar	-1.99	0.5	-1.01
9	C.2	-1.95	926	C.ar	-1.98	0.5	-0.12
21	H.soft	0.93	925	C.ar	-1.67	0.5	-1.53
21	H.soft	0.93	924	N.ar.no_lp	-1.17	0.5	-1.69
23	H.soft	0.98	921	C.ar	-1.57	0.5	-1.54
23	H.soft	0.98	922	C.ar	-1.53	0.5	-1.56
14	N.2	0.88	352	H.soft	0.46	0.5	-1.56

Table S36: 3B1M protein-ligand complex

Ligand atom number	Ligand atom type	Ligand AIP value	Protein atom number	Protein atom type	Protein AIP value	f	$\Delta\Delta G$ (kJ/mol)
51	H.soft	1.16	1525	S.3	-3.5	0.5	-0.35
51	H.soft	1.16	1703	H.soft	0.34	0.5	-1.33
28	C.ar	-1.41	648	H.soft	0.97	0.5	-1.6
0	C.2	-1.77	626	H.soft	0.76	0.5	-1.55
7	C.ar	-0.78	625	O.2.am	-1.35	0.5	-1.5
8	C.ar	-0.58	624	C.2	-1.76	0.5	-1.3
12	C.ar	-1.58	627	H.soft	0.88	0.5	-1.59
16	C.2	-2.52	646	H.soft	0.64	0.5	-1.08
26	C.ar	-1.23	645	S.3	-3.32	0.5	0.75
31	C.ar	-0.69	645	S.3	-3.32	0.5	0.39
55	H.soft	1.01	2004	H.soft	0.35	1.0	-3.0
29	C.ar	-1.49	2000	H.soft	0.25	0.5	-1.77
31	C.ar	-1.16	1149	H.soft	0.34	0.5	-1.92
32	C.ar	-1.46	2000	H.soft	0.25	0.5	-1.79
10	C.ar	-0.74	1355	H.soft	0.33	0.5	-2.05
39	H.soft	1.2	509	H.soft	0.33	0.5	-1.29
39	H.soft	1.2	1525	S.3	0.0	0.5	-1.47
16	C.2	-2.66	1356	H.soft	0.33	0.5	-0.91
61	H.soft	0.34	643	O.2.am	-1.35	0.5	-1.84
35	C.ar	-1.47	1281	H.soft	0.33	0.5	-1.78
52	H.soft	0.66	1152	H.soft	0.33	1.0	-3.66
32	C.ar	-1.11	1888	S.3	-3.5	0.5	0.89
33	C.ar	-1.1	1892	H.soft	0.53	0.5	-1.9
34	C.ar	-0.82	1705	H.soft	0.33	0.5	-2.03
56	H.soft	1.03	1890	H.soft	1.11	0.5	-0.78
56	H.soft	1.03	2003	H.soft	0.33	0.5	-1.49
7	C.ar	-1.75	1348	H.soft	0.22	0.5	-1.61
43	H.soft	1.16	1382	O.3.alcohol	-5.37	0.5	1.24
44	H.soft	1.12	346	H.soft	0.33	1.0	-2.77
57	H.soft	1.0	1885	O.2.am	-1.35	0.5	-1.6
33	C.ar	-1.48	1226	H.soft	0.82	0.5	-1.66
64	H.soft	0.59	1151	H.soft	0.33	1.0	-3.75
1	C.2	-0.19	721	H.soft	0.57	0.5	-2.01
2	C.2	-0.33	721	H.soft	0.57	0.5	-2.02
62	H.soft	0.58	773	H.soft	0.34	0.5	-1.88
62	H.soft	0.58	817	H.soft	0.64	0.5	-1.69
60	H.soft	0.61	771	H.soft	0.34	1.0	-3.72
8	C.ar	-0.27	1353	H.soft	0.33	0.5	-2.12
12	C.ar	-1.25	1353	H.soft	0.33	0.5	-1.88
58	H.soft	1.04	2041	C.ar	-1.56	0.5	-1.51
59	H.soft	0.98	1278	H.soft	0.33	1.0	-3.09
53	H.soft	0.69	772	H.soft	0.34	1.0	-3.6
50	H.soft	1.18	645	S.3	-3.32	1.0	-0.96

Table S37: 3B27 protein-ligand complex

Ligand atom number	Ligand atom type	Ligand AIP value	Protein atom number	Protein atom type	Protein AIP value	f	$\Delta\Delta G$ (kJ/mol)
15	Cl	-1.34	914	H.soft	0.34	0.5	-1.84
7	N.ar	1.14	222	H.soft	0.89	0.5	-0.9
8	C.ar	-0.57	222	H.soft	0.89	0.5	-1.8
10	C.ar	-1.07	217	C.2	-1.66	0.5	-1.09
1	S.3	-2.38	708	C.2	-1.76	0.5	0.1
1	S.3	-2.38	736	H.soft	0.65	0.5	-1.18
14	C.ar	-1.05	917	H.soft	0.33	0.5	-1.96
13	C.ar	-1.09	917	H.soft	0.33	0.5	-1.95
2	C.ar	-1.27	739	H.soft	0.81	0.5	-1.73
3	N.ar	-1.04	739	H.soft	0.81	0.5	-1.8
2	C.ar	-1.25	341	H.soft	0.49	0.5	-1.86
4	N.ar	0.18	341	H.soft	0.49	0.5	-2.01
1	S.3	0.0	706	N.pl3.am	-0.38	0.5	-2.19
12	C.ar	-1.08	1081	H.soft	0.57	0.5	-1.89
13	C.ar	-1.09	1075	C.ar	-1.52	0.5	-1.19
15	Cl	-1.34	1077	C.ar	-1.55	0.5	-1.0
24	H.soft	1.14	1074	C.ar	-1.44	0.5	-1.48
9	C.ar	-2.24	219	N.pl3.am	-0.43	0.5	-0.96
1	S.3	-2.52	340	H.soft	0.49	1.0	-2.12
19	H.N	2.54	592	O.2.other	-5.5	1.0	0.3
20	H.N	2.66	253	O.3.alcohol	-5.37	1.0	0.13

Table S38: 3B5R protein-ligand complex

Ligand atom number	Ligand atom type	Ligand AIP value	Protein atom number	Protein atom type	Protein AIP value	f	$\Delta\Delta G$ (kJ/mol)
29	H.soft	1.44	794	C.ar	-1.82	0.5	-1.13
29	H.soft	1.44	792	C.ar	-1.57	0.5	-1.19
19	C.ar	0.62	947	S.3	-3.5	0.5	-0.19
20	C.ar	0.57	947	S.3	-3.5	0.5	-0.17
21	C.1	-1.82	944	O.2.am	-1.35	0.5	-0.76
21	C.1	-1.38	559	N.pl3.am	-0.26	0.5	-1.71
22	N.1	-2.31	1093	H.soft	0.51	0.5	-1.23
22	N.1	-1.98	951	H.soft	0.53	0.5	-1.46
27	F	0.0	979	N.pl3.am	-0.38	0.5	-2.19
27	F	0.0	943	C.2	-1.76	0.5	-1.59
6	C.ar	0.03	2244	H.soft	0.81	0.5	-1.85
20	C.ar	0.5	1383	H.soft	0.68	0.5	-1.71
0	C.ar	-0.37	862	H.soft	0.81	0.5	-1.87
1	C.ar	0.0	1736	H.soft	0.72	0.5	-1.93
2	F	0.0	2479	H.soft	0.33	0.5	-2.15
2	F	0.0	1736	H.soft	0.72	0.5	-1.93
3	C.ar	-0.25	856	H.soft	0.51	0.5	-2.05
5	C.ar	-0.5	856	H.soft	0.51	0.5	-2.03
40	H.soft	1.62	414	H.soft	0.19	0.5	-0.77
40	H.soft	1.62	557	C.2	-1.35	0.5	-1.03
30	H.soft	1.45	955	H.soft	0.82	1.0	-1.06
11	O.3.alcohol	-1.41	292	C.2	-1.76	0.5	-0.77
11	O.3.alcohol	-1.41	328	N.pl3.am	-0.38	0.5	-1.65
13	C.2	-0.37	300	H.soft	0.33	0.5	-2.11
15	N.pl3.am	0.47	300	H.soft	0.33	0.5	-1.95
31	H.soft	1.4	1868	H.soft	0.39	0.5	-0.97
31	H.soft	1.4	2235	S.3	-3.5	0.5	-0.36
16	C.ar	0.47	304	H.soft	0.33	0.5	-1.95
39	H.soft	1.82	441	N.pl3.am	-0.38	0.5	-0.64
19	C.ar	0.44	415	H.soft	0.34	0.5	-1.96
5	C.ar	0.01	799	C.ar	-1.99	0.5	-1.42
35	H.soft	1.41	1861	O.3.alcohol	-5.34	1.0	1.9
7	C.ar	-0.78	861	H.soft	0.82	0.5	-1.84
21	C.1	-1.89	1096	H.soft	0.65	0.5	-1.51
32	H.soft	1.53	2243	H.soft	0.82	1.0	-0.8
6	C.ar	-0.53	853	S.3	-3.5	0.5	0.51
0	C.ar	0.48	2341	H.soft	0.34	0.5	-1.94
23	C.ar	0.41	954	H.soft	0.84	0.5	-1.63
23	C.ar	0.32	1384	H.soft	0.71	0.5	-1.8
22	N.1	-5.04	1221	H.N	2.7	1.0	0.05
33	H.O	4.52	334	O.2.am	-8.02	1.0	-5.78
37	H.N	3.23	293	O.2.am	-7.21	1.0	-1.07
8	O.3.any	-1.94	1865	H.O	2.85	1.0	1.07

Table S39: 3B65 protein-ligand complex

Ligand atom number	Ligand atom type	Ligand AIP value	Protein atom number	Protein atom type	Protein AIP value	f	$\Delta\Delta G$ (kJ/mol)
37	H.soft	1.45	852	N.pl3.am	-0.38	0.5	-1.19
37	H.soft	1.45	797	C.ar	-1.57	0.5	-1.18
2	I	-1.95	949	O.2.am	-1.35	0.5	-0.65
2	I	-1.95	1098	H.soft	0.51	0.5	-1.48
3	C.ar	0.4	952	S.3	-3.5	0.5	-0.09
4	C.1	-2.01	956	H.soft	0.53	0.5	-1.44
4	C.1	-1.68	1101	H.soft	0.65	0.5	-1.62
5	N.1	-2.11	956	H.soft	0.53	0.5	-1.38
5	N.1	-2.23	1098	H.soft	0.51	0.5	-1.29
6	C.ar	0.3	952	S.3	-3.5	0.5	-0.04
7	C.ar	0.32	450	H.soft	0.76	0.5	-1.76
26	H.soft	1.58	419	H.soft	0.19	0.5	-0.83
26	H.soft	1.58	562	C.2	-1.35	0.5	-1.08
23	C.ar	0.01	799	C.ar	-1.82	0.5	-1.54
24	C.ar	0.05	801	C.ar	-1.67	0.5	-1.65
9	N.pl3.am	0.65	305	H.soft	0.33	0.5	-1.84
10	C.2	0.2	305	H.soft	0.33	0.5	-2.08
13	O.3.alcohol	-0.58	297	C.2	-1.76	0.5	-1.3
13	O.3.alcohol	-0.58	333	N.pl3.am	-0.38	0.5	-2.04
3	C.ar	0.4	1328	H.soft	0.68	0.5	-1.77
2	I	-0.77	1323	C.ar	-1.56	0.5	-1.35
2	I	-0.77	1104	H.soft	0.81	0.5	-1.85
27	H.soft	1.7	446	N.pl3.am	-0.38	0.5	-0.83
19	C.ar	-0.32	804	C.ar	-1.99	0.5	-1.24
20	C.ar	-0.09	802	C.ar	-1.98	0.5	-1.35
33	H.soft	1.69	2134	S.3	-3.5	0.5	-0.34
33	H.soft	1.69	2027	H.soft	0.71	0.5	-0.24
17	C.ar	0.18	2143	H.soft	0.81	0.5	-1.78
35	H.soft	1.31	2240	H.soft	0.34	1.0	-2.27
2	I	-0.74	984	N.pl3.am	-0.38	0.5	-1.99
2	I	-0.74	955	H.soft	0.51	0.5	-2.0
20	C.ar	-0.0	867	H.soft	0.81	0.5	-1.86
23	C.ar	-0.08	861	H.soft	0.51	0.5	-2.04
21	C.1	-1.49	808	H.soft	0.46	0.5	-1.75
18	C.ar	-0.33	2140	H.soft	0.65	0.5	-1.97
2	I	1.87	991	H.soft	0.65	1.0	0.05
8	C.ar	0.53	309	H.soft	0.33	0.5	-1.92
1	C.ar	0.17	959	H.soft	0.84	0.5	-1.76
0	C.ar	-0.95	959	H.soft	0.84	0.5	-1.8
6	C.ar	0.33	420	H.soft	0.34	0.5	-2.02
17	C.ar	-0.14	866	H.soft	0.82	0.5	-1.84
38	H.soft	1.63	960	H.soft	0.82	1.0	-0.47
8	C.ar	0.48	960	H.soft	0.82	0.5	-1.61
4	C.1	-1.13	564	N.pl3.am	-0.26	0.5	-1.85
18	C.ar	-0.15	1839	H.soft	0.39	0.5	-2.1
[illegible]	[illegible]	[illegible]	[illegible]	[illegible]	[illegible]	[illegible]	[illegible]

Table S40: 3B68 protein-ligand complex

Ligand atom number	Ligand atom type	Ligand AIP value	Protein atom number	Protein atom type	Protein AIP value	f	$\Delta\Delta G$ (kJ/mol)
7	C.ar	-1.05	833	C.ar	-1.82	0.5	-0.97
37	H.soft	1.37	831	C.ar	-1.57	0.5	-1.26
37	H.soft	1.37	886	N.pl3.am	-0.38	0.5	-1.3
21	C.ar	0.31	986	S.3	-3.5	0.5	-0.04
22	C.ar	-0.65	986	S.3	-3.5	0.5	0.58
48	H.soft	1.62	573	C.2	-1.35	0.5	-1.03
48	H.soft	1.62	430	H.soft	0.19	0.5	-0.77
28	F	0.0	1018	N.pl3.am	-0.38	0.5	-2.19
28	F	0.0	982	C.2	-1.76	0.5	-1.59
1	C.2	-1.23	848	H.soft	0.55	0.5	-1.85
3	N.pl3.am	-0.41	848	H.soft	0.55	0.5	-2.02
4	C.ar	-0.97	836	C.ar	-1.98	0.5	-0.89
5	C.ar	-1.21	2269	H.soft	0.65	0.5	-1.82
6	C.ar	-1.21	2269	H.soft	0.65	0.5	-1.82
9	C.ar	-1.07	2272	H.soft	0.81	0.5	-1.79
47	H.soft	1.75	457	N.pl3.am	-0.38	0.5	-0.75
21	C.ar	-0.52	431	H.soft	0.34	0.5	-2.09
22	C.ar	-0.75	1422	H.soft	0.68	0.5	-1.92
18	C.ar	0.4	320	H.soft	0.33	0.5	-1.99
33	H.soft	1.21	2533	N.pl3.am	-0.38	0.5	-1.49
4	C.ar	-1.08	901	H.soft	0.81	0.5	-1.79
7	C.ar	-1.15	895	H.soft	0.51	0.5	-1.89
8	C.ar	-1.15	901	H.soft	0.81	0.5	-1.77
13	O.3.alcohol	-1.48	308	C.2	-1.76	0.5	-0.72
15	C.2	-0.41	316	H.soft	0.33	0.5	-2.11
17	N.pl3.am	0.37	316	H.soft	0.33	0.5	-2.0
36	H.soft	1.3	2408	H.soft	0.34	0.5	-1.15
36	H.soft	1.3	2263	S.3	-3.5	0.5	-0.36
40	H.soft	1.09	2263	S.3	-3.5	0.5	-0.33
40	H.soft	1.09	2156	H.soft	0.71	0.5	-1.14
38	H.soft	1.08	892	S.3	-3.5	1.0	-0.66
2	O.2.am	-7.17	2403	H.soft	1.11	0.5	2.97
2	O.2.am	-7.17	2545	H.soft	0.33	0.5	4.62
6	C.ar	-1.17	1930	H.soft	0.39	0.5	-1.91
2	O.2.am	-3.75	2374	H.soft	0.33	0.5	0.23
9	C.ar	-0.77	1927	H.O	2.85	0.5	1.11
39	H.soft	1.48	1927	H.O	2.85	0.5	3.18
39	H.soft	1.48	900	H.soft	0.82	0.5	-0.48
32	H.soft	1.08	710	O.2.am	-1.35	0.5	-1.55
5	C.ar	-2.45	2405	H.soft	0.23	0.5	-1.07
26	C.ar	0.24	993	H.soft	0.84	0.5	-1.73
19	C.ar	-0.34	993	H.soft	0.84	0.5	-1.84
20	C.ar	0.31	461	H.soft	0.76	0.5	-1.76
1	C.2	-1.45	2547	H.soft	0.33	0.5	-1.79
17	N.pl3.am	-1.35	994	H.soft	0.82	0.5	-1.7
[illegible]	[illegible]	[illegible]	[illegible]	[illegible]	[illegible]	[illegible]	[illegible]

Table S41: 3E92 protein-ligand complex

Ligand atom number	Ligand atom type	Ligand AIP value	Protein atom number	Protein atom type	Protein AIP value	f	$\Delta\Delta G$ (kJ/mol)
11	C.ar	-0.99	1322	H.O	2.85	0.5	0.99
17	C.ar	-2.02	1068	H.soft	0.23	0.5	-1.42
24	C.ar	-0.9	1325	H.soft	0.39	0.5	-2.0
35	H.soft	0.97	1322	H.O	2.85	0.5	2.45
35	H.soft	0.97	1068	H.soft	0.23	0.5	-1.61
44	H.soft	0.98	460	H.soft	0.49	1.0	-2.88
31	H.soft	0.77	2000	N.pl3.am	-0.38	0.5	-1.89
31	H.soft	0.77	2003	O.2.am	-7.73	0.5	4.33
5	O.2.one_lp	-0.53	196	C.ar	-1.32	0.5	-1.64
5	O.2.one_lp	-0.51	195	C.ar	-1.53	0.5	-1.51
19	C.2	-2.06	919	H.soft	0.34	0.5	-1.41
22	C.ar	-1.06	922	H.soft	0.33	0.5	-1.96
42	H.soft	1.87	922	H.soft	0.33	1.0	-0.48
11	C.ar	-1.33	1942	H.soft	0.33	0.5	-1.85
13	C.ar	-0.99	1943	H.soft	0.33	0.5	-1.98
15	C.ar	-0.99	1943	H.soft	0.33	0.5	-1.98
40	H.soft	0.72	1938	H.soft	0.33	0.5	-1.78
29	H.soft	0.99	917	H.soft	0.19	0.5	-1.61
29	H.soft	0.99	751	H.soft	0.34	0.5	-1.53
36	H.soft	1.11	193	C.ar	-1.52	0.5	-1.48
37	H.soft	0.92	191	C.ar	-1.42	0.5	-1.63
26	H.soft	1.25	1465	C.2	-1.76	0.5	-1.3
23	C.ar	-0.78	525	H.soft	0.35	0.5	-2.04
24	C.ar	-0.62	525	H.soft	0.35	0.5	-2.07
32	H.soft	0.84	2010	C.ar	-1.56	0.5	-1.61
9	C.ar	-1.13	1944	H.soft	0.33	0.5	-1.93
8	C.ar	-1.52	1944	H.soft	0.33	0.5	-1.75
6	N.ar	-4.12	1425	N.pl3.am	-0.38	0.5	1.21
7	N.ar	-3.93	1395	H.soft	1.11	0.5	0.01
8	C.ar	-1.22	1404	H.soft	0.34	0.5	-1.89
33	H.soft	1.04	1974	H.soft	1.11	1.0	-1.53
46	H.soft	0.75	518	H.soft	0.33	0.5	-1.76
46	H.soft	0.75	508	N.pl3.am	-0.38	0.5	-1.91
45	H.soft	0.95	455	O.2.am	-1.35	0.5	-1.63
45	H.soft	0.95	1245	O.2.am	-1.35	0.5	-1.63
10	C.ar	-1.14	1347	O.2.am	-7.21	0.5	6.12
7	N.ar	-4.08	1761	H.soft	0.49	0.5	0.51
43	H.soft	1.16	1251	H.soft	0.34	0.5	-1.33
43	H.soft	1.16	519	H.soft	0.33	0.5	-1.34
10	C.ar	-1.34	1941	H.soft	0.34	0.5	-1.84
28	H.soft	1.21	1483	C.2	-1.76	0.5	-1.33
34	H.soft	0.85	1347	O.2.am	-1.35	0.5	-1.69
27	H.soft	1.2	202	H.soft	0.85	1.0	-1.72
6	N.ar	-6.83	1469	H.N	2.84	1.0	-0.01
41	H.N	2.51	746	O.2.other	-5.5	1.0	0.33
[illegible]	[illegible]	[illegible]	[illegible]	[illegible]	[illegible]	[illegible]	[illegible]

Table S42: 3FUR protein-ligand complex

Ligand atom number	Ligand atom type	Ligand AIP value	Protein atom number	Protein atom type	Protein AIP value	f	$\Delta\Delta G$ (kJ/mol)
1	C.ar	1.41	1683	C.ar	-1.44	0.5	-1.25
2	C.ar	1.52	1685	C.ar	-1.53	0.5	-1.12
10	O.2.sulfone	-1.1	1740	H.soft	0.84	1.0	-3.53
19	C.ar	-0.9	968	H.soft	0.34	0.5	-2.01
17	C.ar	1.14	502	S.3	-3.32	0.5	-0.48
19	C.ar	0.99	502	S.3	-3.32	0.5	-0.46
24	C.ar	-1.14	481	C.2	-1.76	0.5	-0.97
30	C.ar	-1.16	484	H.soft	0.88	0.5	-1.73
38	H.soft	1.21	497	N.pl3.am	-0.38	0.5	-1.49
12	C.ar	-0.8	920	H.soft	0.94	0.5	-1.75
13	C.ar	0.7	505	H.soft	0.97	0.5	-1.29
3	C.ar	1.84	368	H.soft	0.33	0.5	-0.29
4	Cl	0.47	353	C.2	-1.76	0.5	-1.61
4	Cl	0.59	389	N.pl3.am	-0.38	0.5	-2.0
4	Cl	0.33	1590	H.soft	0.57	0.5	-1.89
4	Cl	0.59	1590	H.soft	0.57	0.5	-1.73
26	N.ar	-1.56	1167	H.soft	0.22	0.5	-1.73
27	C.ar	-1.55	1167	H.soft	0.22	0.5	-1.74
28	C.ar	-1.75	1172	H.soft	0.33	0.5	-1.62
21	C.ar	-0.89	1175	H.soft	0.33	0.5	-2.01
25	C.ar	-1.94	1166	H.soft	1.11	0.5	-1.32
6	C.ar	1.38	1736	H.soft	0.51	0.5	-0.92
36	H.soft	1.62	1733	S.3	-3.5	1.0	-0.7
22	C.ar	-0.47	503	H.soft	0.64	0.5	-1.97
14	C.ar	0.7	503	H.soft	0.64	0.5	-1.6
25	C.ar	-2.04	629	H.soft	0.34	0.5	-1.42
1	C.ar	2.09	506	H.N	1.26	0.5	1.19
5	C.ar	1.66	502	S.3	0.0	0.5	-0.82
7	C.ar	2.02	506	H.N	1.26	0.5	1.05
2	C.ar	1.89	400	H.soft	0.64	0.5	0.05
31	H.soft	1.95	395	C.ar	-1.52	0.5	-0.62
31	H.soft	1.95	407	H.soft	0.68	0.5	0.2
7	C.ar	1.56	1691	H.soft	0.57	0.5	-0.59
4	Cl	0.73	401	H.soft	0.57	0.5	-1.62
16	C.ar	-0.38	971	H.soft	0.33	0.5	-2.11
10	O.2.sulfone	-2.31	1847	H.soft	0.33	1.0	-2.42
13	C.ar	-0.29	969	H.soft	0.33	0.5	-2.12
3	C.ar	1.59	1692	H.soft	0.68	0.5	-0.44
26	N.ar	-6.92	1206	H.N	2.84	1.0	-0.01
9	O.2.sulfone	-3.53	1851	H.N	2.7	1.0	0.04
34	H.N	2.6	914	O.3.any	-3.71	1.0	-0.06

Table S43: 3G0W protein-ligand complex

Ligand atom number	Ligand atom type	Ligand AIP value	Protein atom number	Protein atom type	Protein AIP value	f	$\Delta\Delta G$ (kJ/mol)
6	O.2.one_lp	0.14	314	O.2.am	-1.35	0.5	-1.85
32	H.soft	1.24	314	O.2.am	-1.35	0.5	-1.42
18	C.1	-1.96	585	H.soft	0.78	0.5	-1.44
5	C.2	-1.03	725	H.soft	0.82	0.5	-1.79
16	Cl	-1.39	843	N.pl3.am	-0.38	0.5	-1.66
16	Cl	-2.82	957	H.soft	0.51	0.5	-0.8
18	C.1	-2.71	1013	N.pl3.primary	-0.1	0.5	-0.67
5	C.2	0.57	321	H.soft	0.33	0.5	-1.89
16	Cl	0.0	850	H.soft	0.65	0.5	-1.98
16	Cl	0.0	958	H.soft	0.53	0.5	-2.05
31	H.soft	1.25	435	H.soft	0.19	0.5	-1.31
31	H.soft	1.25	462	N.pl3.am	-0.38	0.5	-1.45
21	F	0.0	1963	H.soft	0.71	1.0	-3.88
33	H.soft	0.67	1568	H.soft	0.33	1.0	-3.64
23	F	0.0	467	H.soft	0.88	1.0	-3.61
26	H.soft	1.5	1737	H.soft	0.39	1.0	-1.65
34	H.soft	0.74	1422	S.3	0.0	0.5	-1.92
34	H.soft	0.74	1188	H.soft	0.71	0.5	-1.51
29	H.soft	1.29	1694	H.soft	0.68	0.5	-0.9
29	H.soft	1.29	1730	O.3.alcohol	-5.34	0.5	1.07
22	F	0.0	664	C.ar	-1.96	0.5	-1.44
6	O.2.one_lp	-0.93	819	H.soft	0.82	0.5	-1.81
30	H.soft	2.18	349	N.pl3.am	-0.38	0.5	-0.01
28	H.soft	1.38	237	H.soft	0.33	0.5	-1.04
28	H.soft	1.38	1693	H.soft	0.7	0.5	-0.76
16	Cl	-0.58	857	H.soft	0.33	0.5	-2.09
16	Cl	-1.48	960	H.soft	0.65	0.5	-1.72
18	C.1	-2.07	437	H.soft	0.34	0.5	-1.4
36	H.O	4.29	355	O.2.am	-7.62	1.0	-4.43
19	N.1	-6.18	1023	H.N	2.7	1.0	0.22

Table S44: 3G2N protein-ligand complex

Ligand atom number	Ligand atom type	Ligand AIP value	Protein atom number	Protein atom type	Protein AIP value	f	$\Delta\Delta G$ (kJ/mol)
27	H.soft	0.76	230	C.2	-1.76	0.5	-1.55
27	H.soft	0.76	233	H.soft	0.88	0.5	-1.33
29	H.soft	0.97	254	H.soft	1.11	1.0	-1.69
2	C.ar	-0.91	1175	H.soft	0.49	0.5	-1.97
4	C.ar	-1.87	542	N.pl3.am	-0.43	0.5	-1.29
5	C.ar	-0.82	540	C.2	-1.66	0.5	-1.25
1	C.ar	-0.93	763	C.2	-0.5	0.5	-1.87
8	N.pl3.am	-1.91	255	H.soft	0.34	0.5	-1.51
6	C.2	-1.96	255	H.soft	0.34	0.5	-1.48
3	C.ar	-0.99	543	H.soft	0.65	0.5	-1.89
0	C.ar	-0.73	1036	H.soft	0.38	0.5	-2.04
1	C.ar	-0.83	1038	H.soft	0.34	0.5	-2.03
20	H.soft	2.15	987	C.2	-1.76	0.5	-0.37
24	H.soft	0.85	548	H.N	2.84	1.0	4.56
31	H.soft	0.67	1336	H.soft	0.33	1.0	-3.64
0	C.ar	-0.9	263	H.soft	0.34	0.5	-2.01
30	H.O	3.31	991	N.ar.no_lp	-0.75	0.5	2.04
28	H.soft	1.09	370	H.soft	0.33	1.0	-2.84
34	H.O	2.85	1774	O.2.other	-5.5	1.0	-0.05
17	O.3.alcohol	-4.63	1839	H.N	2.84	1.0	-0.01
32	H.O	2.92	1471	O.2.am	-8.02	1.0	-0.27
15	O.3.alcohol	-5.56	1861	H.N	2.84	1.0	-0.04
19	O.3.alcohol	-4.72	546	H.N	2.62	1.0	0.04
25	H.N	2.84	988	O.2.am	-7.73	1.0	0.02
10	O.3.any	-2.46	1000	H.N	3.44	1.0	2.02
36	H.O	3.53	1626	O.3.any	-3.71	1.0	0.75

Table S45: 3G2Z protein-ligand complex

Ligand atom number	Ligand atom type	Ligand AIP value	Protein atom number	Protein atom type	Protein AIP value	f	$\Delta\Delta G$ (kJ/mol)
15	H.soft	1.5	263	C.ar	-1.42	0.5	-1.16
15	H.soft	1.5	264	C.ar	-1.45	0.5	-1.15
11	N.ar	0.41	879	O.2.am	-1.35	0.5	-1.83
17	H.soft	1.21	229	N.pl3.am	-0.43	0.5	-1.5
17	H.soft	1.21	271	H.soft	0.51	0.5	-1.15
9	N.ar.no_lp	0.59	925	N.pl3.am	-0.38	0.5	-2.0
21	H.N	4.38	930	O.3.alcohol	-5.38	1.0	-1.26
11	N.ar	-5.98	885	H.O	2.85	1.0	-0.05
12	N.ar	-4.72	497	H.O	2.88	1.0	-0.03
20	H.N	3.75	491	O.2.am	-7.73	1.0	-2.91

Table S46: 3GNW protein-ligand complex

Ligand atom number	Ligand atom type	Ligand AIP value	Protein atom number	Protein atom type	Protein AIP value	f	$\Delta\Delta G$ (kJ/mol)
59	H.soft	1.12	1701	O.2.am	-1.35	0.5	-1.52
33	C.ar	-1.9	2185	H.soft	0.94	0.5	-1.42
43	C.ar	-1.84	2185	H.soft	0.94	0.5	-1.45
38	C.ar	-1.51	581	H.soft	0.61	0.5	-1.72
39	C.ar	-1.22	1241	H.soft	0.33	0.5	-1.9
26	C.ar	-0.68	1066	H.soft	0.97	0.5	-1.74
28	C.ar	-1.13	1061	O.2.am	-1.35	0.5	-1.29
33	C.ar	-1.21	1066	H.soft	0.97	0.5	-1.66
65	H.soft	1.53	1060	C.2	-1.76	0.5	-1.07
65	H.soft	1.53	1086	N.pl3.am	-0.38	0.5	-1.08
56	H.soft	1.02	1700	C.2	-1.76	0.5	-1.44
56	H.soft	1.02	1703	H.soft	0.88	0.5	-1.06
29	C.ar	-3.75	2186	H.soft	0.7	0.5	0.03
30	C.ar	-1.99	2174	C.ar	-1.45	0.5	-0.54
72	H.soft	1.08	1831	O.2.am	-1.35	0.5	-1.55
72	H.soft	1.08	1872	C.ar	-1.45	0.5	-1.52
42	C.ar	-1.38	458	H.soft	0.64	0.5	-1.76
46	H.soft	1.12	262	N.pl3.primary	-0.1	0.5	-1.55
40	C.ar	-1.48	1838	H.soft	0.53	0.5	-1.75
41	C.ar	-1.66	1841	H.soft	0.84	0.5	-1.57
70	H.soft	1.17	572	C.2	-2.4	0.5	-1.07
70	H.soft	1.17	573	N.pl3.primary	-0.1	0.5	-1.49
41	C.ar	-1.37	464	H.soft	0.47	0.5	-1.81
73	H.soft	1.0	1831	O.2.am	-7.73	0.5	3.76
73	H.soft	1.0	464	H.soft	0.47	0.5	-1.43
75	H.soft	0.84	571	N.pl3.primary	-0.1	0.5	-1.82
66	H.soft	0.93	1067	H.N	1.26	1.0	-1.38
32	C.ar	-1.48	1067	H.N	1.26	0.5	-1.37
31	F	-2.41	341	H.soft	0.7	1.0	-2.32
40	C.ar	-1.34	1235	H.soft	0.34	0.5	-1.84
60	H.soft	1.12	1882	H.soft	0.85	1.0	-1.93
52	H.soft	0.56	2180	H.soft	1.11	0.5	-1.24
52	H.soft	0.56	2220	N.pl3.am	-0.38	0.5	-2.02
69	H.soft	1.21	1061	O.2.am	-1.35	0.5	-1.44
71	H.soft	1.17	1120	H.soft	0.52	1.0	-2.39
43	C.ar	-1.32	580	H.soft	0.61	0.5	-1.8
35	C.ar	-3.75	1843	H.soft	0.81	0.5	-0.02
54	H.soft	1.37	2099	H.soft	0.3	1.0	-2.15
55	H.soft	1.1	1721	C.2	-1.66	0.5	-1.44
17	O.2.sulfone	-7.05	2226	H.N	2.84	1.0	-0.0
16	O.2.sulfone	-6.59	2188	H.N	2.84	1.0	-0.02
64	H.O	4.6	1091	O.3.alcohol	-5.38	1.0	-1.42
8	N.ar	-8.52	2573	H.O	2.88	1.0	-0.13

Table S47: 3GR2 protein-ligand complex

Ligand atom number	Ligand atom type	Ligand AIP value	Protein atom number	Protein atom type	Protein AIP value	f	$\Delta\Delta G$ (kJ/mol)
11	N.ar.no_lp	0.04	579	H.soft	0.65	0.5	-1.96
12	N.ar	-2.84	579	H.soft	0.65	0.5	-0.81
18	H.soft	0.6	764	C.ar	-1.45	0.5	-1.74
18	H.soft	0.6	763	C.ar	-1.42	0.5	-1.76
9	C.ar	-3.12	584	H.soft	0.33	0.5	-0.46
7	N.ar.no_lp	-1.6	900	N.pl3.am	-0.38	0.5	-1.52
10	N.ar	-1.47	877	H.soft	0.38	0.5	-1.77
5	C.ar	-0.02	876	H.soft	1.11	0.5	-1.54
12	N.ar	-3.23	615	H.N	2.84	1.0	0.28
10	N.ar	-2.24	906	H.N	2.84	1.0	0.82

Table S48: 3JVR protein-ligand complex

Ligand atom number	Ligand atom type	Ligand AIP value	Protein atom number	Protein atom type	Protein AIP value	f	$\Delta\Delta G$ (kJ/mol)
1	N.ar.no_lp	0.2	316	O.2.am	-1.35	0.5	-1.84
9	N.pl3.am	0.84	316	O.2.am	-7.73	0.5	4.16
11	C.ar	-1.18	360	H.soft	1.11	0.5	-1.56
12	C.ar	-2.27	360	H.soft	1.11	0.5	-1.16
13	C.ar	-3.26	398	H.soft	0.34	0.5	-0.32
15	C.2	-0.86	316	O.2.am	-7.21	0.5	5.97
16	C.ar	0.15	322	H.soft	0.22	0.5	-2.14
21	C.ar	0.09	322	H.soft	0.22	0.5	-2.16
9	N.pl3.am	-0.03	185	C.ar	-1.55	0.5	-1.69
17	C.ar	-0.3	316	O.2.am	-1.35	0.5	-1.72
18	C.ar	-0.31	330	H.soft	0.33	0.5	-2.12
30	H.soft	1.1	390	N.pl3.am	-0.59	0.5	-1.62
30	H.soft	1.1	400	H.soft	0.34	0.5	-1.4
4	C.ar	-1.78	361	H.soft	0.34	0.5	-1.6
3	Cl	-0.33	184	C.ar	-1.53	0.5	-1.59
32	H.soft	1.61	182	C.ar	-1.44	0.5	-1.03
8	C.ar	-1.9	364	H.soft	0.34	0.5	-1.52
10	C.ar	-1.9	399	H.soft	0.34	0.5	-1.52
19	C.ar	-0.2	329	H.soft	0.33	0.5	-2.12
20	C.ar	0.0	329	H.soft	0.33	0.5	-2.15
7	Cl	0.0	954	C.2	-2.23	0.5	-1.21
7	Cl	0.0	991	N.pl3.am	-0.38	0.5	-2.19
7	Cl	-0.75	936	C.2	-1.76	0.5	-1.21
0	C.ar	-1.6	351	N.pl3.am	-0.48	0.5	-1.47
2	O.2.one_lp	0.0	234	H.soft	1.11	1.0	-3.14
2	O.2.one_lp	-0.03	228	C.2	-2.23	0.5	-1.17
21	C.ar	-0.49	186	C.ar	-1.56	0.5	-1.5
3	Cl	-1.03	601	H.soft	0.34	0.5	-1.97
3	Cl	0.0	601	H.soft	0.34	0.5	-2.14
3	Cl	0.0	677	H.O	3.66	0.5	3.33
7	Cl	-1.26	818	H.soft	0.49	0.5	-1.85
31	H.soft	1.34	397	H.soft	0.64	1.0	-1.74
7	Cl	-1.31	1002	H.soft	0.19	0.5	-1.87
27	H.N	3.32	180	O.2.am	-7.21	1.0	-1.31
24	H.N	4.07	229	O.2.am	-7.21	1.0	-3.28

Table S49: 3JVS protein-ligand complex

Ligand atom number	Ligand atom type	Ligand AIP value	Protein atom number	Protein atom type	Protein AIP value	f	$\Delta\Delta G$ (kJ/mol)
4	N.pl3.am	0.16	267	H.soft	1.11	0.5	-1.49
7	N.pl3.am	0.97	218	C.ar	-1.55	0.5	-1.55
34	H.soft	1.06	295	C.2	-2.23	0.5	-1.2
34	H.soft	1.06	346	N.pl3.am	-0.38	0.5	-1.65
11	O.2	-1.87	221	H.soft	0.64	0.5	-1.52
11	O.2	-2.3	664	H.soft	0.48	1.0	-2.47
21	C.ar	0.41	355	H.soft	0.22	0.5	-2.03
20	C.ar	1.0	355	H.soft	0.22	0.5	-1.58
14	C.ar	-1.69	390	C.2	-0.5	0.5	-1.4
0	C.ar	-1.35	393	H.soft	1.11	0.5	-1.52
3	C.ar	-0.81	393	H.soft	1.11	0.5	-1.61
9	C.ar	-1.83	431	H.soft	0.34	0.5	-1.56
19	C.2	0.54	431	H.soft	0.34	0.5	-1.9
12	C.ar	-1.11	348	C.2	-1.76	0.5	-0.99
13	C.ar	-1.36	394	H.soft	0.34	0.5	-1.83
16	C.ar	-1.32	384	N.pl3.am	-0.48	0.5	-1.66
43	H.soft	0.86	1156	N.pl3.am	-0.38	0.5	-1.83
43	H.soft	0.86	1119	C.2	-2.23	0.5	-1.26
51	H.soft	1.03	1167	H.soft	0.19	1.0	-3.13
11	O.2	-1.96	663	H.soft	0.34	0.5	-1.48
15	C.ar	-1.83	432	H.soft	0.34	0.5	-1.56
44	H.soft	0.68	1101	C.2	-1.76	0.5	-1.57
40	H.soft	2.1	215	C.ar	-1.44	0.5	-0.4
40	H.soft	2.1	216	C.ar	-1.52	0.5	-0.41
45	H.soft	0.95	1126	H.soft	1.11	1.0	-1.74
41	H.soft	1.29	423	N.pl3.am	-0.59	0.5	-1.42
41	H.soft	1.29	433	H.soft	0.34	0.5	-1.16
22	C.ar	0.66	363	H.soft	0.33	0.5	-1.83
18	C.ar	-1.72	397	H.soft	0.34	0.5	-1.63
6	C.ar	-1.32	397	H.soft	0.34	0.5	-1.85
1	N.pl3.am	-0.58	261	C.2	-2.23	0.5	-0.89
47	H.soft	0.89	983	H.soft	0.49	1.0	-3.07
25	C.ar	0.83	362	H.soft	0.33	0.5	-1.69
32	H.N	3.66	213	O.2.am	-7.21	1.0	-2.21
30	H.N	2.64	349	O.2.am	-7.21	1.0	0.57

Table S50: 3JYA protein-ligand complex

Ligand atom number	Ligand atom type	Ligand AIP value	Protein atom number	Protein atom type	Protein AIP value	f	$\Delta\Delta G$ (kJ/mol)
8	C.ar	-0.53	312	H.soft	0.33	0.5	-2.09
10	C.ar	0.11	309	H.soft	0.33	0.5	-2.11
15	C.ar	0.17	309	H.soft	0.33	0.5	-2.09
9	C.ar	0.08	1431	H.soft	0.33	0.5	-2.12
10	C.ar	0.17	1274	H.soft	0.33	0.5	-2.09
11	C.ar	-0.01	1274	H.soft	0.33	0.5	-2.12
15	C.ar	0.18	1427	H.soft	0.34	0.5	-2.08
4	Cl	0.61	884	O.2.am	-7.21	0.5	4.11
4	Cl	0.61	931	H.soft	0.65	0.5	-1.66
4	Cl	-0.44	982	H.soft	0.34	0.5	-2.1
0	C.ar	-1.16	1424	H.soft	0.22	0.5	-1.93
5	C.ar	-0.74	1429	H.soft	0.33	0.5	-2.05
1	N.ar.no_lp	1.62	1429	H.soft	0.33	0.5	-0.67
1	N.ar.no_lp	0.86	568	H.soft	0.35	0.5	-1.65
5	C.ar	-0.71	212	C.ar	-1.52	0.5	-1.42
4	Cl	-0.75	932	H.soft	0.34	0.5	-2.05
11	C.ar	-0.08	497	H.soft	0.49	0.5	-2.05
6	N.ar	0.04	214	C.ar	-1.55	0.5	-1.73
13	C.ar	-0.21	121	H.soft	0.33	0.5	-2.12
12	C.ar	-0.2	121	H.soft	0.33	0.5	-2.12
12	C.ar	-0.16	1273	H.soft	0.33	0.5	-2.12
18	H.soft	1.49	1051	H.soft	0.33	0.5	-0.88
18	H.soft	1.49	935	H.soft	0.34	0.5	-0.87
14	C.ar	-0.12	1271	H.soft	0.34	0.5	-2.12
17	H.soft	2.0	211	C.ar	-1.44	0.5	-0.54
17	H.soft	2.0	213	C.ar	-1.53	0.5	-0.55
4	Cl	-0.92	725	H.soft	0.23	0.5	-2.02
2	O.2.am	-6.77	571	H.N	2.7	1.0	0.32
2	O.2.am	-5.7	1464	H.N	2.84	1.0	-0.04

Table S51: 3K5V protein-ligand complex

Ligand atom number	Ligand atom type	Ligand AIP value	Protein atom number	Protein atom type	Protein AIP value	f	$\Delta\Delta G$ (kJ/mol)
36	H.soft	1.32	982	C.2	-0.5	0.5	-1.38
8	C.ar	-0.71	1069	H.soft	0.34	0.5	-2.06
11	N.pl3.aniline	-0.17	1018	O.2.am	-1.35	0.5	-1.76
9	C.ar	-0.86	1039	H.soft	0.64	0.5	-1.92
22	C.ar	-0.46	1696	H.soft	0.34	0.5	-2.1
23	C.ar	-0.44	1696	H.soft	0.34	0.5	-2.1
33	H.soft	1.72	1020	H.soft	0.88	1.0	-0.02
10	C.ar	-0.87	1070	H.soft	0.34	0.5	-2.02
13	N.ar	-1.9	224	H.soft	0.49	0.5	-1.52
14	C.ar	-1.56	224	H.soft	0.49	0.5	-1.71
16	C.ar	-0.54	1582	H.soft	0.33	0.5	-2.09
32	H.soft	1.07	217	N.pl3.am	-0.38	0.5	-1.64
18	C.ar	-0.53	1699	H.soft	0.33	0.5	-2.09
19	C.ar	-1.03	1699	H.soft	0.33	0.5	-1.97
16	C.ar	-1.14	858	H.soft	0.85	0.5	-1.75
15	N.ar	-3.04	858	H.soft	0.85	0.5	-0.67
5	C.ar	-0.91	1185	H.soft	0.33	0.5	-2.01
6	C.ar	-1.09	1482	H.soft	0.34	0.5	-1.95
30	H.soft	1.22	1038	S.3	-3.32	0.5	-0.48
30	H.soft	1.22	1182	H.soft	0.33	0.5	-1.26
9	C.ar	-0.98	793	H.soft	0.64	0.5	-1.9
8	C.ar	-0.87	793	H.soft	0.64	0.5	-1.92
13	N.ar	-4.41	323	H.soft	0.34	1.0	2.03
7	C.ar	-2.14	323	H.soft	0.34	0.5	-1.35
17	C.ar	-0.05	1581	H.soft	0.33	0.5	-2.12
3	F	0.0	308	C.2	-1.76	0.5	-1.59
3	F	0.0	309	O.2.am	-1.35	0.5	-1.84
29	H.soft	1.48	794	H.soft	0.49	1.0	-1.56
0	F	0.0	1341	C.ar	-1.55	0.5	-1.73
27	H.soft	1.13	220	O.2.am	-7.73	1.0	6.87
10	C.ar	-0.97	795	H.soft	0.49	0.5	-1.95
6	C.ar	-1.25	315	H.soft	0.34	0.5	-1.88

Table S52: 3KR8 protein-ligand complex

Ligand atom number	Ligand atom type	Ligand AIP value	Protein atom number	Protein atom type	Protein AIP value	f	$\Delta\Delta G$ (kJ/mol)
0	N.ar.no_lp	1.3	1375	H.soft	0.51	0.5	-1.03
1	C.ar	0.09	1367	C.ar	-1.42	0.5	-1.81
10	C.ar	0.4	286	H.soft	0.64	0.5	-1.8
11	C.ar	0.24	1380	H.soft	0.7	0.5	-1.84
12	C.ar	0.36	286	H.soft	0.64	0.5	-1.83
18	C.ar	0.53	1368	C.ar	-1.45	0.5	-1.76
4	C.ar	-1.08	967	C.2	-1.76	0.5	-1.01
17	S.3	-2.69	979	H.soft	0.5	0.5	-0.92
17	S.3	-2.69	1017	N.pl3.am	-0.38	0.5	-0.54
17	S.3	-2.74	1073	H.soft	0.49	0.5	-0.87
17	S.3	-2.74	1758	H.soft	0.34	0.5	-0.84
17	S.3	0.0	1020	O.2.am	-1.35	0.5	-1.84
30	H.soft	0.96	1019	C.2	-1.76	0.5	-1.47
30	H.soft	0.96	1066	N.pl3.am	-0.38	0.5	-1.74
2	N.ar	-4.42	702	C.ar	-1.45	0.5	2.34
2	N.ar	0.21	1370	C.ar	-1.44	0.5	-1.8
3	C.ar	0.11	1371	C.ar	-1.53	0.5	-1.74
4	C.ar	0.41	1369	C.ar	-1.52	0.5	-1.74
11	C.ar	-1.17	709	H.soft	0.51	0.5	-1.88
23	H.soft	1.27	701	C.ar	-1.42	0.5	-1.38
26	H.soft	1.18	1378	H.soft	0.85	0.5	-0.89
26	H.soft	1.18	1229	H.soft	0.24	0.5	-1.37
28	H.soft	1.0	1372	C.ar	-1.32	0.5	-1.61
0	N.ar.no_lp	1.1	227	C.ar	-2.05	0.5	-1.28
22	H.soft	2.41	228	N.ar	-1.88	0.5	-0.04
25	H.soft	1.41	703	C.ar	-1.52	0.5	-1.23
25	H.soft	1.41	1530	H.soft	0.34	0.5	-0.99
29	H.soft	1.04	971	C.ar	-1.45	0.5	-1.55
29	H.soft	1.04	970	C.ar	-1.42	0.5	-1.55
2	N.ar	-0.26	978	H.soft	0.51	0.5	-2.05
1	C.ar	-0.29	978	H.soft	0.51	0.5	-2.05
8	F	0.0	632	C.2	-1.76	0.5	-1.59
17	S.3	0.0	1757	H.soft	0.34	0.5	-2.14
13	C.ar	0.08	708	H.soft	1.11	0.5	-1.53
5	C.ar	-1.03	225	N.ar.no_lp	-0.75	0.5	-1.71
31	H.soft	0.93	1259	N.pl3.am	-0.38	0.5	-1.77
6	O.2.am	-5.97	268	H.N	2.84	1.0	-0.04
21	H.N	3.62	265	O.2.am	-7.73	1.0	-2.5

Table S53: 3L7B protein-ligand complex

Ligand atom number	Ligand atom type	Ligand AIP value	Protein atom number	Protein atom type	Protein AIP value	f	$\Delta\Delta G$ (kJ/mol)
27	H.soft	1.1	242	H.soft	1.11	1.0	-1.38
19	H.soft	0.9	221	H.soft	0.88	1.0	-2.38
25	H.soft	0.84	218	C.2	-1.76	0.5	-1.53
29	H.soft	1.99	924	O.2.am	-1.35	0.5	-0.55
29	H.soft	1.99	924	O.2.am	-7.73	0.5	1.53
6	F	-1.45	1671	H.N	2.84	0.5	0.74
6	F	-1.45	1638	N.pl3.am	-0.38	0.5	-1.62
30	H.soft	1.53	974	H.soft	0.34	1.0	-1.62
15	C.ar	-0.46	528	C.2	-1.66	0.5	-1.44
16	C.ar	-1.86	523	N.pl3.am	-0.38	0.5	-1.32
17	C.ar	-4.11	530	N.pl3.am	-0.43	0.5	1.23
17	C.ar	-3.64	243	H.soft	0.34	0.5	0.1
16	C.ar	-0.96	249	H.soft	0.33	0.5	-1.99
14	C.ar	-0.62	933	H.soft	0.49	0.5	-2.03
3	N.ar	-0.88	244	H.soft	0.33	0.5	-2.02
23	H.soft	0.92	1194	H.soft	0.33	1.0	-3.21
28	H.O	3.68	927	N.ar.no_lp	-0.75	0.5	2.79
21	H.O	3.46	1606	O.2.other	-5.5	1.0	-0.65
9	O.3.alcohol	-4.37	1693	H.N	2.84	1.0	0.01
24	H.O	3.11	1329	O.2.am	-8.02	1.0	-0.94
4	O.3.alcohol	-3.86	534	H.N	2.62	1.0	-0.04
18	O.2.am	-10.01	252	H.N	2.84	1.0	0.09
7	N.3.aniline	-0.4	536	H.N	2.84	1.0	2.59
13	O.3.alcohol	-3.18	936	H.N	3.44	1.0	1.18
18	O.2.am	-7.9	222	H.N	2.84	1.0	0.02

Table S54: 3O9I protein-ligand complex

Ligand atom number	Ligand atom type	Ligand AIP value	Protein atom number	Protein atom type	Protein AIP value	f	$\Delta\Delta G$ (kJ/mol)
39	C.ar	-2.34	591	C.2	-1.76	0.5	0.06
71	H.soft	0.58	593	H.soft	0.76	1.0	-3.18
1	N.ar	-2.14	1376	H.soft	0.49	0.5	-1.36
11	O.2.sulfone	-2.3	1993	H.soft	0.34	1.0	-2.44
11	O.2.sulfone	-3.53	621	H.soft	0.33	1.0	-0.04
40	C.ar	-2.27	607	N.pl3.am	-0.38	0.5	-0.96
44	H.soft	1.46	1997	H.soft	0.33	0.5	-0.93
80	H.soft	0.75	616	H.soft	0.22	1.0	-3.63
39	C.ar	-1.3	1894	H.soft	0.34	0.5	-1.86
38	C.ar	-2.39	1894	H.soft	0.34	0.5	-1.15
70	H.soft	0.51	1108	N.pl3.primary	-0.1	0.5	-2.04
8	C.ar	0.44	1312	C.2	-1.76	0.5	-1.61
45	H.soft	1.43	1370	O.2.am	-1.71	0.5	-1.17
45	H.soft	1.43	1316	H.soft	0.49	0.5	-0.86
36	C.ar	-3.19	1922	H.soft	0.33	0.5	-0.38
37	C.ar	-3.01	1922	H.soft	0.33	0.5	-0.57
49	H.soft	0.26	1606	H.soft	0.76	0.5	-1.79
58	H.soft	0.86	828	H.soft	0.34	1.0	-3.31
54	H.soft	1.37	1294	C.2	-1.76	0.5	-1.21
69	H.soft	0.71	383	H.soft	0.64	1.0	-3.18
1	N.ar	-1.49	1333	N.pl3.am	-0.48	0.5	-1.55
6	C.ar	1.14	1315	H.soft	0.64	0.5	-1.14
62	H.soft	0.69	927	H.soft	0.34	0.5	-1.8
62	H.soft	0.69	931	H.soft	0.33	0.5	-1.81
61	H.soft	0.51	1631	H.soft	0.23	1.0	-3.95
0	C.ar	-0.36	1342	H.soft	0.49	0.5	-2.05
79	H.soft	0.8	615	H.soft	1.11	1.0	-2.06
48	H.soft	1.01	302	O.2.other	-0.1	0.5	-1.67
55	H.soft	0.97	302	O.2.other	-5.5	1.0	3.19
4	C.ar	0.42	1555	H.soft	0.22	0.5	-2.03
7	C.ar	-0.53	1445	H.soft	0.33	0.5	-2.09
8	C.ar	-0.12	1445	H.soft	0.33	0.5	-2.12
75	H.soft	0.5	1234	O.2.other	-0.1	0.5	-2.05
66	H.soft	0.78	384	H.soft	0.49	1.0	-3.27
67	H.soft	0.92	444	H.soft	0.49	1.0	-3.01
53	H.soft	1.19	1234	O.2.other	-0.1	0.5	-1.47
73	H.soft	0.84	542	H.soft	0.22	1.0	-3.48
63	H.soft	0.67	857	H.soft	0.33	1.0	-3.64
41	C.ar	-2.6	1994	H.soft	0.34	0.5	-0.96
65	H.soft	0.82	1634	H.soft	0.33	1.0	-3.4
59	H.O	3.82	301	O.2.other	-5.5	1.0	-0.97
29	O.3.any	-5.96	446	H.N	2.84	1.0	-0.04
31	O.3.any	-6.57	412	H.N	2.84	1.0	-0.02
1	N.ar	-3.82	1378	H.N	2.84	1.0	0.11
64	H.N	1.71	363	O.2.am	-7.21	1.0	3.59

Table S55: 3P5O protein-ligand complex

Ligand atom number	Ligand atom type	Ligand AIP value	Protein atom number	Protein atom type	Protein AIP value	f	$\Delta\Delta G$ (kJ/mol)
34	H.soft	1.43	464	H.soft	0.33	1.0	-1.94
21	C.ar	-1.71	464	H.soft	0.33	0.5	-1.64
30	H.soft	1.08	212	C.ar	-1.87	0.5	-1.37
12	C.ar	-0.25	599	H.soft	0.33	0.5	-2.12
13	C.ar	-0.61	599	H.soft	0.33	0.5	-2.08
3	C.ar	-1.11	1395	H.soft	0.34	0.5	-1.94
5	C.ar	-0.73	1392	H.soft	0.22	0.5	-2.08
7	C.2	-1.42	1395	H.soft	0.34	0.5	-1.8
18	C.ar	-2.43	1393	H.soft	0.23	0.5	-1.09
8	N.2	-2.76	1393	H.soft	0.23	0.5	-0.78
9	C.ar	0.56	274	H.soft	0.47	0.5	-1.82
12	C.ar	0.34	268	H.soft	0.64	0.5	-1.84
13	C.ar	0.05	268	H.soft	0.64	0.5	-1.97
16	C.ar	0.37	274	H.soft	0.47	0.5	-1.93
31	H.soft	0.8	213	C.ar	-1.99	0.5	-1.42
37	H.soft	1.26	388	N.pl3.am	-0.26	0.5	-1.42
2	C.ar	-1.09	1498	S.3	0.0	0.5	-1.97
42	H.soft	1.03	273	H.soft	0.48	0.5	-1.39
42	H.soft	1.03	1396	H.soft	0.34	0.5	-1.48
9	C.ar	-0.35	598	H.soft	0.33	0.5	-2.11
10	C.ar	0.26	598	H.soft	0.33	0.5	-2.05
43	H.soft	0.81	673	H.soft	0.33	0.5	-1.71
40	H.soft	0.95	298	C.ar	-1.55	0.5	-1.56
27	N.pl3.am	-0.64	1200	H.soft	0.85	0.5	-1.83
45	H.soft	0.72	1399	H.soft	0.33	1.0	-3.56
44	H.soft	0.76	1193	C.ar	-1.53	0.5	-1.66
11	C.ar	-0.23	459	H.soft	0.33	0.5	-2.12
25	C.2	-1.94	670	H.soft	0.34	0.5	-1.49
2	C.ar	-1.2	214	C.ar	-1.96	0.5	-0.75
19	N.ar	-8.45	1247	H.N	2.62	1.0	0.94

Table S56: 3PRS protein-ligand complex

Ligand atom number	Ligand atom type	Ligand AIP value	Protein atom number	Protein atom type	Protein AIP value	f	$\Delta\Delta G$ (kJ/mol)
7	C.2	-1.8	893	H.soft	0.69	0.5	-1.55
9	N.pl3.am	-0.36	893	H.soft	0.69	0.5	-1.95
66	H.soft	0.8	1744	C.ar	-1.56	0.5	-1.63
53	H.soft	0.91	890	H.soft	1.11	1.0	-1.83
56	H.soft	1.26	1933	O.3.alcohol	-5.34	0.5	1.1
56	H.soft	1.26	1851	O.2.other	-5.5	0.5	1.24
60	H.soft	0.77	2289	H.soft	0.34	0.5	-1.74
60	H.soft	0.77	1937	H.O	2.85	0.5	2.21
80	H.soft	0.43	1940	H.soft	0.39	0.5	-1.94
80	H.soft	0.43	1937	H.O	2.85	0.5	1.88
19	C.ar	-2.24	2219	H.soft	0.33	0.5	-1.27
69	H.soft	0.9	885	C.ar	-1.52	0.5	-1.6
69	H.soft	0.9	883	C.ar	-1.42	0.5	-1.64
30	N.pl3.am	-0.77	958	H.soft	0.49	0.5	-2.0
78	H.soft	0.31	948	N.pl3.am	-0.48	0.5	-2.11
77	H.soft	1.19	1933	O.3.alcohol	-5.34	1.0	2.36
85	H.soft	0.82	1964	O.3.alcohol	-5.34	1.0	3.23
27	C.ar	-1.29	1095	H.soft	0.7	0.5	-1.78
28	C.ar	-1.24	1326	H.soft	0.34	0.5	-1.89
16	C.ar	-2.2	2290	H.soft	0.34	0.5	-1.3
17	C.ar	-2.26	2290	H.soft	0.34	0.5	-1.25
61	H.soft	0.64	1851	O.2.other	-0.1	0.5	-1.97
61	H.soft	0.64	1850	C.2	-0.5	0.5	-1.97
93	H.soft	0.94	1173	O.2.other	-0.1	0.5	-1.73
59	H.soft	0.65	892	H.soft	0.5	1.0	-3.47
16	C.ar	-5.0	935	H.soft	0.88	0.5	1.19
44	C.ar	-0.48	377	H.soft	0.49	0.5	-2.04
91	H.soft	0.88	372	C.2	-0.5	0.5	-1.81
63	H.soft	0.7	2162	H.soft	0.33	1.0	-3.6
32	C.2	-0.39	953	C.2	-0.5	0.5	-2.06
65	H.soft	0.77	1745	C.ar	-1.56	0.5	-1.64
65	H.soft	0.77	1743	C.ar	-1.55	0.5	-1.65
90	H.soft	0.87	236	H.soft	0.34	1.0	-3.29
42	C.ar	-0.49	370	O.2.am	-7.73	0.5	6.5
74	H.soft	1.12	1329	H.soft	0.33	1.0	-2.77
1	C.ar	-0.97	827	H.soft	0.34	0.5	-1.99
21	O.3.alcohol	-2.19	629	H.soft	0.88	1.0	-2.56
83	H.soft	0.59	2037	C.ar	-1.53	0.5	-1.71
73	H.soft	1.08	481	C.2	-0.5	0.5	-1.64
15	C.ar	-2.06	1797	H.soft	0.34	0.5	-1.41
0	C.ar	-1.75	825	H.soft	0.23	0.5	-1.61
3	C.ar	-2.08	1540	H.soft	0.33	0.5	-1.39
57	H.soft	1.02	887	C.ar	-1.53	0.5	-1.53
67	H.O	3.92	1852	O.2.other	-5.5	1.0	-1.06
76	H.N	2.6	955	O.2.other	-5.5	1.0	0.22
[illegible]	[illegible]	[illegible]	[illegible]	[illegible]	[illegible]	[illegible]	[illegible]

Table S57: 3PYY protein-ligand complex

Ligand atom number	Ligand atom type	Ligand AIP value	Protein atom number	Protein atom type	Protein AIP value	f	$\Delta\Delta G$ (kJ/mol)
20	N.pl3.am	1.2	1114	C.2	-1.76	0.5	-1.34
22	O.2.am	-4.63	1137	H.soft	0.94	0.5	0.76
22	O.2.am	-4.63	1130	N.pl3.am	-0.38	0.5	1.91
22	O.2.am	1.28	891	H.soft	0.49	0.5	-1.07
3	C.ar	-0.54	890	H.soft	0.64	0.5	-1.97
7	C.ar	-0.61	885	N.pl3.am	-0.38	0.5	-2.03
32	H.soft	1.13	892	H.soft	0.49	0.5	-1.27
32	H.soft	1.13	713	O.2.am	-1.35	0.5	-1.51
35	H.soft	1.49	890	H.soft	0.64	1.0	-1.28
9	C.ar	-0.46	1166	H.soft	0.34	0.5	-2.1
19	N.ar.no_lp	-0.83	1166	H.soft	0.34	0.5	-2.03
11	C.ar	0.35	331	H.soft	0.34	0.5	-2.01
4	C.ar	-0.78	323	H.soft	0.34	0.5	-2.04
6	C.ar	-0.96	231	H.soft	0.49	0.5	-1.95
8	C.ar	-0.79	316	C.2	-1.76	0.5	-1.19
13	C.ar	-0.45	324	H.soft	0.33	0.5	-2.1
14	C.ar	-0.5	323	H.soft	0.34	0.5	-2.1
18	N.ar	-0.06	363	H.soft	0.19	0.5	-2.17
19	N.ar.no_lp	-0.09	231	H.soft	0.49	0.5	-2.05
29	H.soft	1.02	363	H.soft	0.19	0.5	-1.58
29	H.soft	1.02	352	N.pl3.am	-0.38	0.5	-1.69
33	H.soft	1.17	317	O.2.am	-1.35	0.5	-1.48
33	H.soft	1.17	360	H.soft	1.11	0.5	-0.6
11	C.ar	-0.88	1136	H.soft	0.64	0.5	-1.92
22	O.2.am	0.59	1136	H.soft	0.64	0.5	-1.68
12	C.ar	-0.98	1168	H.soft	0.34	0.5	-1.99
5	C.ar	-1.09	1168	H.soft	0.34	0.5	-1.95
3	C.ar	-1.27	1236	H.soft	0.33	0.5	-1.87
4	C.ar	-1.75	1239	H.soft	0.33	0.5	-1.62
7	C.ar	-1.27	1236	H.soft	0.33	0.5	-1.87
10	C.ar	-1.3	1239	H.soft	0.33	0.5	-1.86
18	N.ar	-0.83	1169	H.soft	0.34	0.5	-2.03
8	C.ar	-1.24	1366	H.soft	0.7	0.5	-1.79
14	C.ar	-1.54	1167	H.soft	0.34	0.5	-1.74

Table S58: 3RLR protein-ligand complex

Ligand atom number	Ligand atom type	Ligand AIP value	Protein atom number	Protein atom type	Protein AIP value	f	$\Delta\Delta G$ (kJ/mol)
7	C.ar	-0.97	1173	H.soft	0.33	0.5	-1.99
11	C.ar	-1.08	1173	H.soft	0.33	0.5	-1.95
21	Cl	-1.22	958	H.soft	0.82	0.5	-1.74
15	C.ar	-1.01	959	H.soft	0.81	0.5	-1.8
2	C.ar	0.43	375	H.soft	0.89	0.5	-1.57
3	C.ar	0.36	375	H.soft	0.89	0.5	-1.62
7	C.ar	-2.01	1397	C.ar	-1.44	0.5	-0.53
8	C.ar	-1.26	370	C.2	-1.66	0.5	-0.97
10	C.ar	-1.3	372	N.pl3.am	-0.43	0.5	-1.69
11	C.ar	-2.44	1398	C.ar	-1.52	0.5	-0.04
12	C.ar	-1.52	1404	H.soft	0.57	0.5	-1.72
18	N.1	-2.39	1404	H.soft	0.57	0.5	-1.17
27	H.soft	1.18	1168	H.soft	0.19	0.5	-1.39
27	H.soft	1.18	1399	C.ar	-1.53	0.5	-1.42
33	H.soft	1.13	370	C.2	-1.66	0.5	-1.42
4	C.ar	-0.04	1882	H.soft	0.34	0.5	-2.11
15	C.ar	-0.25	494	H.soft	0.49	0.5	-2.05
1	N.ar.no_lp	0.45	1880	H.soft	0.38	0.5	-1.94
31	H.soft	1.38	266	O.2.am	-1.35	0.5	-1.29
31	H.soft	1.38	401	N.pl3.am	-0.38	0.5	-1.29
21	Cl	0.15	1170	H.soft	0.34	0.5	-2.09
9	C.1	-2.36	1410	H.soft	0.68	0.5	-1.2
29	H.soft	1.49	1959	H.soft	0.33	1.0	-1.76
1	N.ar.no_lp	1.49	407	H.soft	0.64	0.5	-0.64
18	N.1	-1.54	277	H.soft	0.33	0.5	-1.74
18	N.1	-2.17	374	H.soft	0.84	0.5	-1.3
9	C.1	-1.73	374	H.soft	0.84	0.5	-1.54
24	H.soft	0.78	926	N.pl3.am	-0.38	0.5	-1.89
23	H.N	4.06	812	O.2.other	-5.5	1.0	-1.18
18	N.1	-5.61	377	H.N	2.49	1.0	0.4
0	N.ar	-5.15	1881	H.O	2.85	1.0	-0.05

Table S59: 3RR4 protein-ligand complex

Ligand atom number	Ligand atom type	Ligand AIP value	Protein atom number	Protein atom type	Protein AIP value	f	$\Delta\Delta G$ (kJ/mol)
0	N.ar.no_lp	-0.5	370	C.ar	-1.53	0.5	-1.51
2	C.ar	-2.47	371	C.ar	-1.32	0.5	-0.16
4	C.ar	-3.25	369	C.ar	-1.44	0.5	0.82
8	C.ar	-2.69	1448	H.soft	0.65	0.5	-0.94
6	C.ar	-2.72	1448	H.soft	0.65	0.5	-0.92
12	C.ar	-2.94	770	H.soft	0.94	0.5	-0.76
14	N.ar.no_lp	-0.11	770	H.soft	0.94	0.5	-1.73
7	C.ar	-1.88	1451	H.soft	0.81	0.5	-1.47
6	C.ar	-2.68	379	H.soft	0.7	0.5	-0.95
8	C.ar	-2.68	367	C.ar	-1.45	0.5	0.16
9	C.ar	-2.57	368	C.ar	-1.52	0.5	0.1
17	N.ar	-1.97	366	C.ar	-1.42	0.5	-0.58
7	C.ar	-1.7	374	H.soft	0.51	0.5	-1.64
2	C.ar	-2.15	1479	H.soft	0.88	0.5	-1.3
0	N.ar.no_lp	-0.47	1479	H.soft	0.88	0.5	-1.81
5	C.ar	-3.39	772	H.N	1.26	0.5	-0.43
14	N.ar.no_lp	-0.4	860	H.soft	0.34	0.5	-2.11
12	C.ar	-3.25	1442	S.3	-3.5	0.5	2.84
1	N.ar	-1.14	1229	H.soft	0.33	0.5	-1.93
27	H.soft	0.8	325	N.pl3.am	-0.38	0.5	-1.87
27	H.soft	0.8	301	C.2	-1.76	0.5	-1.54
28	H.soft	0.83	309	H.N	2.84	0.5	2.26
28	H.soft	0.83	274	H.soft	0.49	0.5	-1.59
1	N.ar	-1.14	1203	H.soft	0.49	0.5	-1.9
29	H.soft	1.19	375	H.soft	0.5	1.0	-2.37
25	H.N	3.31	697	O.2.other	-5.5	1.0	-0.51
13	O.2.am	-9.31	1145	H.N	2.84	1.0	0.06
13	O.2.am	-8.15	941	H.N	2.7	1.0	0.56
24	H.N	2.44	1160	O.2.am	-7.21	1.0	1.16
26	H.N	3.18	696	O.2.other	-5.5	1.0	-0.38

Table S60: 3RSX protein-ligand complex

Ligand atom number	Ligand atom type	Ligand AIP value	Protein atom number	Protein atom type	Protein AIP value	f	$\Delta\Delta G$ (kJ/mol)
4	C.ar	-1.76	629	H.soft	1.17	0.5	-1.36
14	C.ar	-1.75	611	N.pl3.am	-0.38	0.5	-1.41
25	H.soft	1.31	570	C.2	-1.76	0.5	-1.26
25	H.soft	1.31	577	H.soft	0.64	0.5	-0.91
6	N.ar	-0.73	148	O.2.other	-0.1	0.5	-2.06
8	C.ar	-1.25	436	C.ar	-1.32	0.5	-1.23
2	C.ar	-1.72	432	C.ar	-1.45	0.5	-0.77
3	C.ar	-1.71	439	H.soft	0.51	0.5	-1.63
4	C.ar	-1.77	439	H.soft	0.51	0.5	-1.6
13	C.ar	-1.71	431	C.ar	-1.42	0.5	-0.81
14	C.ar	-1.77	367	O.2.am	-1.35	0.5	-0.81
16	H.soft	1.04	433	C.ar	-1.52	0.5	-1.52
16	H.soft	1.04	435	C.ar	-1.53	0.5	-1.52
12	C.ar	-1.81	869	H.soft	0.7	0.5	-1.54
3	C.ar	-1.78	870	H.soft	0.68	0.5	-1.56
5	C.ar	-1.78	863	H.soft	0.64	0.5	-1.57
22	H.N	2.16	147	O.2.other	-5.5	1.0	0.79
21	H.N	2.41	1287	O.2.other	-5.5	1.0	0.46

Table S61: 3SYR protein-ligand complex

Ligand atom number	Ligand atom type	Ligand AIP value	Protein atom number	Protein atom type	Protein AIP value	f	$\Delta\Delta G$ (kJ/mol)
31	H.soft	1.11	245	H.soft	1.11	1.0	-1.35
22	H.soft	1.18	224	H.soft	0.88	1.0	-1.71
29	H.soft	0.95	221	C.2	-1.76	0.5	-1.48
9	N.ar.no_lp	-0.43	246	H.soft	0.34	0.5	-2.1
6	C.ar	-0.93	246	H.soft	0.34	0.5	-2.0
20	H.soft	2.2	979	O.2.am	-1.35	0.5	-0.24
23	H.soft	1.54	979	O.2.am	-7.73	1.0	4.95
2	C.ar	0.28	531	C.2	-1.66	0.5	-1.67
3	C.ar	-1.14	526	N.pl3.am	-0.38	0.5	-1.81
5	N.ar.no_lp	1.05	497	C.2	-0.5	0.5	-1.67
6	C.ar	-0.73	533	N.pl3.am	-0.43	0.5	-1.98
3	C.ar	-0.98	252	H.soft	0.33	0.5	-1.99
27	H.soft	0.95	1249	H.soft	0.33	1.0	-3.15
5	N.ar.no_lp	0.72	247	H.soft	0.33	0.5	-1.78
0	C.ar	0.46	988	H.soft	0.49	0.5	-1.87
30	H.soft	1.25	361	H.soft	0.33	0.5	-1.22
30	H.soft	1.25	1829	H.soft	0.39	0.5	-1.18
11	O.3.alcohol	-2.21	537	H.N	2.62	1.0	0.3
19	O.3.alcohol	-3.32	991	H.N	3.44	1.0	1.03
32	H.O	3.6	1415	O.2.am	-7.62	1.0	-2.36
26	H.O	4.09	1718	O.2.other	-5.5	1.0	-1.21
13	O.3.alcohol	-2.6	1783	H.N	2.84	1.0	0.59
28	H.O	3.28	1415	O.2.am	-8.02	1.0	-1.54
15	O.3.alcohol	-4.34	1805	H.N	2.84	1.0	0.02
7	O.2.am	-5.13	255	H.N	2.84	1.0	-0.04
21	H.N	3.29	498	O.2.other	-5.5	1.0	-0.49
24	H.O	4.23	1570	O.3.any	-3.71	1.0	1.42
7	O.2.am	-5.48	225	H.N	2.84	1.0	-0.04

Table S62: 3U5J protein-ligand complex

Ligand atom number	Ligand atom type	Ligand AIP value	Protein atom number	Protein atom type	Protein AIP value	f	$\Delta\Delta G$ (kJ/mol)
0	C.ar	0.96	1289	H.soft	0.34	0.5	-1.56
1	C.ar	0.76	238	H.soft	0.47	0.5	-1.67
2	C.ar	0.66	232	H.soft	0.64	0.5	-1.63
4	C.ar	0.65	232	H.soft	0.64	0.5	-1.64
17	C.ar	-0.95	1289	H.soft	0.34	0.5	-1.99
21	C.ar	-0.73	1286	H.soft	0.22	0.5	-2.08
30	H.soft	0.87	238	H.soft	0.47	0.5	-1.57
30	H.soft	0.87	177	C.ar	-1.99	0.5	-1.4
2	C.ar	0.49	560	H.soft	0.34	0.5	-1.93
4	C.ar	0.37	563	H.soft	0.33	0.5	-2.0
24	H.soft	1.69	428	H.soft	0.33	1.0	-1.11
8	C.ar	-1.3	428	H.soft	0.33	0.5	-1.86
0	C.ar	0.76	562	H.soft	0.33	0.5	-1.75
6	C.ar	0.82	562	H.soft	0.33	0.5	-1.7
28	H.soft	1.06	601	H.soft	0.33	0.5	-1.46
31	H.soft	1.1	176	C.ar	-1.87	0.5	-1.36
12	C.ar	-2.0	1287	H.soft	0.23	0.5	-1.44
7	N.ar.no_lp	-0.02	1287	H.soft	0.23	0.5	-2.15
20	C.ar	-0.9	1277	N.pl3.am	-0.38	0.5	-1.92
25	H.soft	1.26	262	C.ar	-1.55	0.5	-1.36
27	H.soft	1.28	237	H.soft	0.48	0.5	-1.08
27	H.soft	1.28	227	C.2	-1.78	0.5	-1.27
18	C.ar	-1.25	178	C.ar	-1.96	0.5	-0.71

Table S63: 3WZ8 protein-ligand complex

Ligand atom number	Ligand atom type	Ligand AIP value	Protein atom number	Protein atom type	Protein AIP value	f	$\Delta\Delta G$ (kJ/mol)
16	C.ar	-4.31	704	H.soft	0.88	0.5	0.48
17	C.ar	-2.94	701	C.2	-1.76	0.5	0.7
18	C.ar	-2.66	704	H.soft	0.88	0.5	-0.97
31	C.ar	-0.51	883	C.ar	-1.56	0.5	-1.49
56	H.soft	1.27	778	H.O	2.88	1.0	5.85
39	H.soft	0.83	968	O.2.other	-0.1	0.5	-1.83
39	H.soft	0.83	966	C.2	-0.5	0.5	-1.85
28	C.ar	-0.44	1121	H.soft	0.34	0.5	-2.1
29	C.ar	-0.5	1124	H.soft	0.33	0.5	-2.1
30	C.ar	-0.51	881	C.ar	-1.55	0.5	-1.49
4	N.pl3.aniline	-0.66	332	H.soft	0.49	0.5	-2.02
5	C.ar	-1.75	332	H.soft	0.49	0.5	-1.61
9	C.ar	-2.15	301	H.soft	0.49	0.5	-1.35
10	C.ar	-2.03	294	O.2.am	-1.71	0.5	-0.29
1	C.2	-0.75	1361	H.soft	1.11	0.5	-1.61
23	C.2	-1.57	722	C.2	-0.5	0.5	-1.48
57	H.soft	1.23	723	O.2.other	-0.1	0.5	-1.42
14	C.ar	-0.38	1359	O.3.alcohol	-5.34	0.5	2.92
12	C.ar	-1.22	1359	O.3.alcohol	-5.34	0.5	3.48
27	C.ar	-0.3	654	C.ar	-1.52	0.5	-1.61
32	C.ar	-0.51	652	C.ar	-1.42	0.5	-1.58
51	H.soft	1.33	661	H.soft	0.5	0.5	-1.0
51	H.soft	1.33	662	H.soft	0.69	0.5	-0.84
15	C.ar	-1.11	1363	H.O	2.85	0.5	0.93
21	C.ar	-1.86	1666	H.soft	0.34	0.5	-1.54
22	C.ar	-1.19	1366	H.soft	0.39	0.5	-1.9
14	C.ar	-2.02	727	H.soft	0.49	0.5	-1.44
12	C.ar	-0.42	727	H.soft	0.49	0.5	-2.05
15	C.ar	-2.47	717	N.pl3.am	-0.48	0.5	-0.71
17	C.ar	-1.85	1540	H.soft	0.33	0.5	-1.55
49	H.soft	1.51	1536	H.soft	0.34	1.0	-1.69
18	C.ar	-1.94	1667	H.soft	0.34	0.5	-1.49
8	C.ar	-2.21	160	H.soft	0.34	0.5	-1.29
7	C.ar	-2.18	160	H.soft	0.34	0.5	-1.31
36	H.soft	1.27	1390	O.3.alcohol	-5.34	1.0	2.19
38	H.soft	0.8	1066	H.soft	0.33	1.0	-3.43
19	C.ar	-1.94	1596	H.soft	0.33	0.5	-1.49
50	H.N	2.91	1339	O.2.am	-7.21	1.0	-0.2
24	O.2.am	-6.39	705	H.N	2.84	1.0	-0.02
24	O.2.am	-7.77	728	H.N	2.84	1.0	0.02
37	H.N	2.45	407	O.2.other	-5.5	1.0	0.4

Table S64: 3TWJ protein-ligand complex

Ligand atom number	Ligand atom type	Ligand AIP value	Protein atom number	Protein atom type	Protein AIP value	f	$\Delta\Delta G$ (kJ/mol)
14	C.1	-2.38	599	H.soft	0.94	0.5	-1.15
15	N.1	-2.93	722	H.soft	0.94	0.5	-0.77
4	N.ar	-4.9	233	N.ar.no_lp	-1.17	0.5	2.82
5	C.ar	-0.33	233	N.ar.no_lp	-1.17	0.5	-1.81
18	H.soft	1.56	234	C.ar	-1.67	0.5	-1.06
20	H.soft	1.64	236	C.ar	-1.87	0.5	-0.94
20	H.soft	1.64	883	C.ar	-1.98	0.5	-0.92
22	H.soft	1.56	232	C.ar	-1.82	0.5	-1.03
22	H.soft	1.56	230	C.ar	-1.57	0.5	-1.07
6	C.ar	0.83	1556	S.3	-3.5	0.5	-0.27
14	C.1	-2.19	510	H.soft	0.7	0.5	-1.31
15	N.1	-2.97	492	N.pl3.am	-0.38	0.5	-0.23
5	C.ar	-0.48	1560	H.soft	0.53	0.5	-2.03
4	N.ar	-0.13	1560	H.soft	0.53	0.5	-2.03
4	N.ar	0.09	231	C.ar	-1.53	0.5	-1.74
23	H.soft	1.59	139	C.ar	-1.32	0.5	-1.07
23	H.soft	1.59	138	C.ar	-1.53	0.5	-1.05
3	Cl	0.0	1341	N.pl3.am	-0.48	0.5	-2.17
3	Cl	0.0	1463	O.2.am	-1.35	0.5	-1.84
21	H.soft	1.7	237	C.ar	-1.99	0.5	-0.86
21	H.soft	1.7	238	C.ar	-1.96	0.5	-0.87
1	C.ar	0.75	292	H.soft	1.11	0.5	-1.08
2	C.ar	-0.59	292	H.soft	1.11	0.5	-1.61
3	Cl	-0.12	295	H.soft	0.34	0.5	-2.12
3	Cl	-0.15	1471	H.soft	0.19	0.5	-2.17
14	C.1	-2.41	723	H.soft	0.7	0.5	-1.16
15	N.1	-2.01	711	C.ar	-1.45	0.5	-0.52
24	H.soft	1.76	712	C.ar	-1.52	0.5	-0.85
14	C.1	-2.81	505	H.soft	0.51	0.5	-0.81
12	C.2	0.71	715	C.ar	-1.32	0.5	-1.76
13	N.2	1.08	713	C.ar	-1.44	0.5	-1.52
15	N.1	-7.14	512	H.N	2.84	1.0	-0.0

Table S65: 4BKT protein-ligand complex

Ligand atom number	Ligand atom type	Ligand AIP value	Protein atom number	Protein atom type	Protein AIP value	f	$\Delta\Delta G$ (kJ/mol)
1	C.ar	-2.17	941	C.ar	-1.32	0.5	-0.46
3	O.2.one_lp	-3.53	1068	H.soft	1.27	0.5	-0.34
3	O.2.one_lp	-2.12	616	H.soft	0.69	0.5	-1.36
3	O.2.one_lp	-2.12	1068	H.soft	1.27	0.5	-1.15
4	C.ar	-1.79	937	C.ar	-1.45	0.5	-0.71
6	C.2	-2.03	948	H.soft	0.94	0.5	-1.35
17	C.ar	-3.41	939	C.ar	-1.44	0.5	1.01
23	H.soft	1.12	479	C.ar	-1.96	0.5	-1.31
20	H.soft	0.94	940	C.ar	-1.53	0.5	-1.58
20	H.soft	0.94	215	H.soft	0.65	0.5	-1.36
31	H.soft	1.28	1146	C.ar	-1.87	0.5	-1.24
31	H.soft	1.28	471	C.ar	-1.57	0.5	-1.33
33	H.soft	1.06	477	C.ar	-1.87	0.5	-1.38
33	H.soft	1.06	475	C.ar	-1.67	0.5	-1.46
30	H.soft	1.34	700	C.ar	-1.32	0.5	-1.33
30	H.soft	1.34	699	C.ar	-1.53	0.5	-1.29
32	H.soft	1.04	476	C.ar	-1.98	0.5	-1.34
32	H.soft	1.04	473	C.ar	-1.82	0.5	-1.41
9	C.2	-1.55	706	H.soft	0.85	0.5	-1.61
29	H.soft	1.0	1154	H.soft	0.81	1.0	-2.3
3	O.2.one_lp	-3.44	609	C.ar	-1.56	0.5	1.14
35	H.O	3.7	910	O.3.alcohol	-5.37	1.0	-0.75
25	H.N	2.63	865	O.2.am	-7.73	1.0	0.73
18	O.3.alcohol	-3.72	1069	H.N	3.44	1.0	0.66

Table S66: 4CR9 protein-ligand complex

Ligand atom number	Ligand atom type	Ligand AIP value	Protein atom number	Protein atom type	Protein AIP value	f	$\Delta\Delta G$ (kJ/mol)
2	C.ar	-1.7	853	C.2	-1.76	0.5	-0.54
3	C.ar	-2.74	911	N.pl3.am	-0.38	0.5	-0.48
1	C.ar	-1.41	579	C.2	-1.76	0.5	-0.77
4	N.ar	-1.79	583	H.soft	0.64	0.5	-1.57
5	C.ar	-2.73	583	H.soft	0.64	0.5	-0.91
6	C.ar	-2.05	599	N.pl3.am	-0.38	0.5	-1.16
7	C.ar	-2.27	608	H.soft	0.64	0.5	-1.26
8	C.ar	-2.78	609	H.soft	0.33	0.5	-0.79
14	H.soft	0.86	580	O.2.am	-1.35	0.5	-1.68
14	H.soft	0.86	694	N.pl3.am	-0.38	0.5	-1.83
3	C.ar	-1.99	557	O.2.am	-1.35	0.5	-0.61
1	C.ar	-1.61	865	H.soft	1.11	0.5	-1.45
6	C.ar	-1.77	865	H.soft	1.11	0.5	-1.39
2	C.ar	-1.44	556	C.2	-1.76	0.5	-0.75
13	H.soft	0.84	825	N.pl3.am	-0.38	0.5	-1.84
13	H.soft	0.84	827	C.2	-1.76	0.5	-1.53
19	H.soft	0.63	970	C.2	-1.76	0.5	-1.58
16	H.soft	1.01	805	H.soft	0.36	1.0	-2.99
22	H.N	2.04	971	O.2.am	-7.21	1.0	2.43
23	H.N	1.52	526	O.2.other	-5.5	1.0	1.91

Table S67: 4CRA protein-ligand complex

Ligand atom number	Ligand atom type	Ligand AIP value	Protein atom number	Protein atom type	Protein AIP value	f	$\Delta\Delta G$ (kJ/mol)
27	C.ar	-2.07	1519	H.soft	0.24	0.5	-1.39
25	C.ar	-2.07	1490	H.soft	0.64	0.5	-1.4
26	C.ar	-2.06	1490	H.soft	0.64	0.5	-1.4
31	N.ar	-3.29	1464	O.2.am	-7.21	0.5	7.97
32	C.ar	-1.3	1464	O.2.am	-1.35	0.5	-1.18
33	C.ar	-0.95	1463	C.2	-1.76	0.5	-1.09
3	C.ar	-0.93	1553	H.soft	0.76	0.5	-1.85
25	C.ar	-1.9	1818	N.pl3.am	-0.38	0.5	-1.29
30	C.ar	-0.89	1772	H.soft	1.11	0.5	-1.6
32	C.ar	-1.93	1760	C.2	-1.76	0.5	-0.34
33	C.ar	-1.0	1758	N.pl3.am	-0.38	0.5	-1.88
35	C.ar	-0.95	1772	H.soft	1.11	0.5	-1.6
4	C.ar	-0.79	433	H.soft	0.72	0.5	-1.9
5	C.ar	-0.35	1205	H.soft	0.34	0.5	-2.11
6	C.ar	-0.71	1205	H.soft	0.34	0.5	-2.06
8	C.ar	-0.79	989	H.soft	0.94	0.5	-1.75
0	C.2	-2.66	1517	H.soft	0.33	0.5	-0.91
2	C.ar	-1.18	1549	N.pl3.am	-0.38	0.5	-1.78
30	C.ar	-1.39	1486	C.2	-1.76	0.5	-0.79
61	H.soft	0.98	1609	H.soft	0.52	1.0	-2.84
50	H.soft	1.08	479	H.soft	0.33	1.0	-2.87
19	C.ar	-1.33	600	H.soft	0.72	0.5	-1.75
20	C.ar	-1.5	636	H.soft	0.64	0.5	-1.71
12	N.ar.no_lp	-0.49	1203	H.soft	0.23	0.5	-2.13
9	C.ar	-1.25	1202	H.soft	0.23	0.5	-1.89
46	H.soft	1.38	594	C.ar	-2.17	0.5	-1.07
16	C.2	-2.45	1515	H.soft	0.64	0.5	-1.13
56	H.soft	1.2	1712	H.soft	0.36	1.0	-2.54
39	H.soft	1.47	427	C.ar	-2.17	0.5	-1.0
39	H.soft	1.47	465	C.2	-1.76	0.5	-1.12
4	C.ar	-0.49	471	H.soft	1.11	0.5	-1.61
53	H.soft	0.68	1877	C.2	-1.76	0.5	-1.57
17	O.2.am	-7.23	1555	H.N	2.84	1.0	0.0
42	H.N	2.9	423	O.2.am	-7.73	1.0	-0.18
57	H.N	1.76	1433	O.2.other	-5.5	1.0	1.44
17	O.2.am	-6.62	1611	H.N	2.84	1.0	-0.02
58	H.N	2.29	1878	O.2.am	-7.21	1.0	1.62

Table S68: 4DE1 protein-ligand complex

Ligand atom number	Ligand atom type	Ligand AIP value	Protein atom number	Protein atom type	Protein AIP value	f	$\Delta\Delta G$ (kJ/mol)
10	C.ar	-0.6	1267	H.soft	0.76	0.5	-1.89
8	C.ar	-1.14	912	C.2	-1.76	0.5	-0.97
9	C.ar	-0.61	913	O.2.am	-1.35	0.5	-1.58
26	H.soft	1.21	1281	N.pl3.am	-0.48	0.5	-1.5
4	C.ar	-0.21	919	H.soft	0.84	0.5	-1.83
3	C.ar	-1.33	919	H.soft	0.84	0.5	-1.7
7	N.ar	1.28	953	H.soft	1.11	0.5	-0.44
3	C.ar	-1.5	303	N.pl3.am	-0.43	0.5	-1.57
32	H.soft	1.03	570	H.soft	0.52	1.0	-2.73
1	C.2	-1.39	917	N.pl3.am	-0.43	0.5	-1.64
2	C.ar	-0.53	915	C.2	-1.66	0.5	-1.4
30	H.soft	1.02	629	N.pl3.am	-0.43	0.5	-1.69
30	H.soft	1.02	303	N.pl3.am	-0.43	0.5	-1.69
29	H.soft	2.35	1240	O.2.am	-1.35	0.5	-0.01
15	C.ar	-0.56	338	C.ar	-1.45	0.5	-1.54
6	N.ar.no_lp	-0.27	957	H.soft	0.36	0.5	-2.11
19	N.ar.no_lp	0.17	1247	H.N	2.84	0.5	1.63
15	C.ar	-1.13	628	O.2.am	-1.23	0.5	-1.37
24	H.soft	1.98	815	O.2.am	-1.8	0.5	-0.58
24	H.soft	1.98	814	C.2	-1.78	0.5	-0.58
31	H.soft	1.2	346	H.soft	0.5	0.5	-1.17
31	H.soft	1.2	350	H.soft	0.7	0.5	-1.01
23	H.soft	1.09	824	H.soft	0.48	1.0	-2.65
18	C.ar	-0.99	1223	H.soft	0.76	0.5	-1.84
33	H.N	4.53	1242	O.3.alcohol	-5.38	1.0	-1.37
0	O.2.am	-6.12	633	H.N	2.62	1.0	0.35
0	O.2.am	-6.3	307	H.N	2.62	1.0	0.4
22	N.ar	-5.68	571	H.O	2.88	1.0	-0.09
21	N.ar	-6.57	1197	H.O	2.85	1.0	-0.04

Table S69: 4DE3 protein-ligand complex

Ligand atom number	Ligand atom type	Ligand AIP value	Protein atom number	Protein atom type	Protein AIP value	f	$\Delta\Delta G$ (kJ/mol)
5	C.ar	-0.36	912	H.soft	0.84	0.5	-1.84
3	C.ar	-0.28	1260	H.soft	0.76	0.5	-1.9
17	N.ar	1.82	1230	N.pl3.am	-0.38	0.5	-0.64
4	C.ar	-0.36	1274	N.pl3.am	-0.48	0.5	-2.08
22	H.soft	1.4	1274	N.pl3.am	-0.48	0.5	-1.27
8	C.ar	-0.36	1279	C.2	-0.5	0.5	-2.07
23	H.soft	0.99	817	H.soft	0.48	0.5	-1.44
23	H.soft	0.99	808	O.2.am	-1.8	0.5	-1.44
12	C.ar	-1.49	621	O.2.am	-1.23	0.5	-1.12
27	H.soft	1.11	339	H.soft	0.5	0.5	-1.29
27	H.soft	1.11	620	C.2	-1.66	0.5	-1.43
13	C.ar	-1.27	165	H.O	2.88	0.5	0.9
14	C.ar	-1.0	165	H.O	2.88	0.5	1.04
12	C.ar	-0.99	331	C.ar	-1.45	0.5	-1.31
28	H.soft	1.04	563	H.soft	0.52	1.0	-2.71
26	H.soft	0.89	622	N.pl3.am	-0.43	0.5	-1.81
16	C.ar	-0.5	1216	H.soft	0.76	0.5	-1.9
20	N.ar	-0.25	163	H.soft	0.54	0.5	-2.03
19	N.ar.no_lp	-2.95	1183	C.2	-1.76	0.5	0.71
1	C.2	-1.44	910	N.pl3.am	-0.43	0.5	-1.61
29	H.soft	1.28	1232	C.2	-1.76	0.5	-1.28
5	C.ar	-1.73	296	N.pl3.am	-0.43	0.5	-1.4
20	N.ar	-5.03	564	H.O	2.88	1.0	-0.06
0	O.2.am	-6.4	300	H.N	2.62	1.0	0.42
0	O.2.am	-6.22	626	H.N	2.62	1.0	0.38
25	H.N	3.17	1233	O.2.am	-7.21	1.0	-0.91

Table S70: 4DLI protein-ligand complex

Ligand atom number	Ligand atom type	Ligand AIP value	Protein atom number	Protein atom type	Protein AIP value	f	$\Delta\Delta G$ (kJ/mol)
18	C.ar	-1.22	342	C.ar	-1.96	0.5	-0.73
7	C.ar	-2.45	343	H.soft	1.11	0.5	-1.06
9	C.ar	-2.51	347	H.soft	1.17	0.5	-1.01
11	C.ar	-2.93	335	C.ar	-1.53	0.5	0.51
9	C.ar	-2.54	1069	H.N	2.84	0.5	0.31
11	C.ar	-3.25	1033	N.pl3.am	-0.38	0.5	0.1
19	C.ar	-2.54	1010	H.soft	0.33	0.5	-1.01
25	H.soft	0.71	1059	N.pl3.am	-0.38	0.5	-1.93
7	C.ar	-2.48	1067	H.soft	0.52	0.5	-1.1
1	C.ar	-2.73	334	C.ar	-1.57	0.5	0.31
2	N.ar	-2.43	338	C.ar	-1.67	0.5	0.07
3	C.ar	-2.72	336	C.ar	-1.82	0.5	0.51
4	C.ar	-1.16	339	C.ar	-1.98	0.5	-0.76
10	C.ar	-2.6	344	H.soft	0.49	0.5	-0.99
13	C.ar	-1.31	341	C.ar	-1.99	0.5	-0.64
14	C.ar	-2.66	340	C.ar	-1.87	0.5	0.48
19	C.ar	-2.47	344	H.soft	0.49	0.5	-1.1
27	H.soft	0.46	337	N.ar.no_lp	-1.17	0.5	-1.89
1	C.ar	-2.92	1005	H.soft	0.23	0.5	-0.62
0	N.ar	-1.48	1005	H.soft	0.23	0.5	-1.78
2	N.ar	-2.43	1003	H.soft	1.11	0.5	-1.07
12	C.ar	-1.45	969	H.soft	0.35	0.5	-1.79
18	C.ar	-2.23	969	H.soft	0.35	0.5	-1.28
28	H.soft	1.22	1400	C.2	-0.5	0.5	-1.49
28	H.soft	1.22	1341	O.2.other	-0.1	0.5	-1.43
4	C.ar	-1.19	849	H.soft	0.33	0.5	-1.91
13	C.ar	-1.42	849	H.soft	0.33	0.5	-1.8
31	H.soft	0.78	1008	H.soft	0.34	0.5	-1.73
34	H.soft	0.81	1300	O.2.am	-1.35	0.5	-1.71
24	H.soft	0.74	265	H.soft	0.33	1.0	-3.53
6	N.3.aniline	-2.12	963	H.soft	0.33	1.0	-2.72
36	H.soft	1.98	149	H.soft	1.11	1.0	1.52
33	H.soft	0.26	123	H.soft	0.48	1.0	-3.96
14	C.ar	-2.72	954	C.2	-1.76	0.5	0.46
21	H.N	2.54	1401	O.2.other	-5.5	1.0	0.3

Table S71: 4EKY protein-ligand complex

Ligand atom number	Ligand atom type	Ligand AIP value	Protein atom number	Protein atom type	Protein AIP value	f	$\Delta\Delta G$ (kJ/mol)
0	N.ar.no_lp	-0.69	272	H.soft	0.34	0.5	-2.06
7	C.ar	-0.08	280	H.soft	0.34	0.5	-2.12
21	O.3.any	-2.84	263	N.pl3.am	-0.38	0.5	-0.37
21	O.3.any	-2.84	272	H.soft	0.34	0.5	-0.74
42	H.soft	1.11	271	H.soft	1.11	1.0	-1.35
25	H.soft	2.05	1073	O.2.am	-1.35	0.5	-0.47
25	H.soft	2.05	1082	H.soft	0.3	0.5	0.08
34	H.soft	1.4	1073	O.2.am	-7.73	1.0	5.59
40	H.soft	0.86	247	C.2	-1.76	0.5	-1.52
40	H.soft	0.86	250	H.soft	0.88	0.5	-1.23
1	C.ar	-0.96	559	N.pl3.am	-0.43	0.5	-1.88
3	N.ar.no_lp	0.58	523	C.2	-0.5	0.5	-2.0
4	C.ar	-1.75	552	N.pl3.am	-0.38	0.5	-1.41
6	C.ar	-0.65	557	C.2	-1.66	0.5	-1.34
31	H.soft	0.66	1321	C.2	-0.5	0.5	-1.96
30	H.soft	0.65	819	C.2	-0.5	0.5	-1.97
30	H.soft	0.65	888	N.ar	-1.88	0.5	-1.51
4	C.ar	-1.61	278	H.soft	0.33	0.5	-1.7
3	N.ar.no_lp	0.3	273	H.soft	0.33	0.5	-2.04
38	H.soft	0.87	1595	H.soft	0.33	0.5	-1.65
38	H.soft	0.87	2104	H.soft	0.49	0.5	-1.55
41	H.soft	1.23	387	H.soft	0.33	0.5	-1.25
41	H.soft	1.23	2175	H.soft	0.39	0.5	-1.21
26	H.soft	1.0	1256	C.2	-1.76	0.5	-1.46
27	H.soft	0.97	1123	H.soft	0.34	0.5	-1.55
27	H.soft	0.97	820	O.2.other	-0.1	0.5	-1.7
33	H.soft	0.65	1328	H.soft	0.34	1.0	-3.66
43	H.O	3.6	1076	N.ar	-8.56	1.0	-3.05
15	O.3.alcohol	-2.38	563	H.N	2.62	1.0	0.23
37	H.O	3.61	2064	O.2.other	-5.5	1.0	-0.78
19	O.3.alcohol	-4.83	2151	H.N	2.84	1.0	-0.03
17	O.3.alcohol	-4.31	2129	H.N	2.84	1.0	0.02
39	H.O	3.13	1761	O.2.am	-8.02	1.0	-1.01
2	O.2.am	-5.6	281	H.N	2.84	1.0	-0.04
24	H.N	2.99	524	O.2.other	-5.5	1.0	-0.2
23	O.3.alcohol	-2.51	1085	H.N	3.29	1.0	1.62
2	O.2.am	-5.89	251	H.N	2.84	1.0	-0.04
35	H.O	3.92	1916	O.3.any	-3.71	1.0	1.12

Table S72: 4F9W protein-ligand complex

Ligand atom number	Ligand atom type	Ligand AIP value	Protein atom number	Protein atom type	Protein AIP value	f	$\Delta\Delta G$ (kJ/mol)
17	C.ar	-1.53	572	H.soft	0.35	0.5	-1.74
21	C.ar	-1.42	1079	N.pl3.am	-0.38	0.5	-1.64
21	C.ar	-1.64	1017	H.soft	0.34	0.5	-1.68
22	C.ar	-1.05	1089	H.soft	0.34	0.5	-1.96
22	C.ar	-1.34	567	H.soft	0.33	0.5	-1.84
40	H.soft	1.01	1011	O.2.am	-1.35	0.5	-1.59
40	H.soft	1.01	503	O.2.am	-1.35	0.5	-1.59
39	H.soft	0.97	1046	N.pl3.am	-0.38	0.5	-1.74
39	H.soft	0.97	1049	O.2.am	-1.35	0.5	-1.62
18	C.ar	-1.66	1767	O.2.other	-0.1	0.5	-1.59
20	C.ar	-1.45	1087	H.soft	0.38	0.5	-1.78
19	C.ar	-1.42	1087	H.soft	0.38	0.5	-1.8
37	H.soft	0.99	1766	C.2	-0.5	0.5	-1.72
3	C.ar	-0.55	1738	H.soft	0.33	0.5	-2.09
6	C.ar	-0.75	1737	H.soft	0.33	0.5	-2.05
7	C.ar	-0.21	1737	H.soft	0.33	0.5	-2.12
11	N.ar	-1.24	507	H.soft	0.49	0.5	-1.86
6	C.ar	-1.64	343	H.soft	0.33	0.5	-1.68
23	C.ar	-1.79	566	H.soft	0.33	0.5	-1.59
24	C.ar	-1.88	566	H.soft	0.33	0.5	-1.53
20	C.ar	-1.64	1010	C.2	-1.76	0.5	-0.59
42	H.soft	0.93	340	H.soft	0.33	1.0	-3.19
13	C.ar	-0.47	340	H.soft	0.33	0.5	-2.1
41	H.soft	1.0	525	C.2	-1.76	0.5	-1.46
41	H.soft	1.0	502	C.2	-1.76	0.5	-1.46
18	C.ar	-1.4	773	H.soft	0.33	0.5	-1.81
16	C.ar	-0.55	773	H.soft	0.33	0.5	-2.09
23	C.ar	-1.3	508	H.soft	0.49	0.5	-1.84
10	C.ar	-1.76	508	H.soft	0.49	0.5	-1.6
38	H.soft	0.96	734	H.soft	0.33	0.5	-1.56
38	H.soft	0.96	733	H.soft	0.33	0.5	-1.56
11	N.ar	-1.38	1191	N.pl3.am	-0.38	0.5	-1.67
9	C.ar	-0.53	1736	H.soft	0.34	0.5	-2.09
14	C.ar	-0.24	1739	H.soft	0.33	0.5	-2.12
11	N.ar	-7.28	1207	H.N	2.84	1.0	0.0
5	N.ar	-8.09	574	H.N	2.7	1.0	0.55

Table S73: 4GFM protein-ligand complex

Ligand atom number	Ligand atom type	Ligand AIP value	Protein atom number	Protein atom type	Protein AIP value	f	$\Delta\Delta G$ (kJ/mol)
5	C.2	-0.1	327	H.soft	0.33	0.5	-2.12
7	C.2	-1.86	638	H.soft	0.33	0.5	-1.54
14	Cl	0.0	160	C.2	-1.76	0.5	-1.59
14	Cl	0.0	158	N.pl3.am	-0.38	0.5	-2.19
14	Cl	-0.3	178	C.2	-1.76	0.5	-1.43
24	H.soft	1.59	176	N.pl3.am	-0.38	0.5	-1.0
5	C.2	0.84	1425	H.soft	0.33	0.5	-1.68
16	Cl	1.83	1425	H.soft	0.33	0.5	-0.31
9	N.pl3.am	0.55	1422	H.soft	0.34	0.5	-1.9
4	C.2	-0.7	1422	H.soft	0.34	0.5	-2.06
16	Cl	0.76	1337	C.2	-1.76	0.5	-1.55
18	H.soft	1.67	1343	H.soft	0.65	1.0	-0.67
14	Cl	-0.75	163	H.soft	0.88	0.5	-1.8
4	C.2	-0.51	330	H.soft	0.33	0.5	-2.1
9	N.pl3.am	0.93	330	H.soft	0.33	0.5	-1.59
15	C.ar	0.79	219	N.pl3.am	-0.38	0.5	-1.88
17	C.ar	0.81	224	H.soft	0.88	0.5	-1.28
20	H.soft	1.34	783	S.3	-3.5	1.0	-0.72
3	C.2	-2.26	1424	H.soft	0.33	0.5	-1.25
1	C.ar	1.29	1588	H.soft	0.49	0.5	-1.06
2	C.ar	0.93	1584	C.2	-0.5	0.5	-1.77
16	Cl	1.03	1588	H.soft	0.49	0.5	-1.39
16	Cl	0.93	1563	C.2	-1.76	0.5	-1.49
7	C.2	-1.77	790	H.soft	0.84	0.5	-1.52
14	Cl	-0.96	325	H.soft	0.23	0.5	-2.01
14	Cl	-3.55	122	C.2	-1.76	0.5	1.44
10	N.2	0.14	818	O.2.am	-1.71	0.5	-1.63
12	O.2.carbonyl	-6.94	924	H.N	2.84	1.0	-0.01

Table S74: 4HGE protein-ligand complex

Ligand atom number	Ligand atom type	Ligand AIP value	Protein atom number	Protein atom type	Protein AIP value	f	$\Delta\Delta G$ (kJ/mol)
34	H.soft	1.09	229	C.2	-1.76	0.5	-1.41
11	N.ar	1.03	445	H.soft	0.33	0.5	-1.49
23	C.ar	0.29	445	H.soft	0.33	0.5	-2.04
11	N.ar	1.3	1740	H.soft	0.34	0.5	-1.15
10	C.ar	-0.81	1740	H.soft	0.34	0.5	-2.03
2	C.ar	-0.21	1741	H.soft	0.33	0.5	-2.12
3	C.ar	-1.22	1161	H.soft	0.34	0.5	-1.89
12	N.ar.no_lp	0.83	1742	H.soft	0.33	0.5	-1.69
14	N.ar	1.25	1161	H.soft	0.34	0.5	-1.22
37	H.soft	1.97	1029	S.3	-3.5	1.0	-0.55
2	C.ar	-0.47	204	H.soft	0.33	0.5	-2.1
5	C.2	-2.02	204	H.soft	0.33	0.5	-1.43
23	C.ar	0.53	1743	H.soft	0.33	0.5	-1.92
24	C.ar	1.52	884	H.soft	0.33	0.5	-0.83
14	N.ar	1.17	618	H.soft	0.49	0.5	-1.22
7	C.ar	-2.71	199	H.soft	0.33	0.5	-0.86
8	C.ar	-1.8	191	C.2	-1.76	0.5	-0.45
7	C.ar	-2.5	1297	H.soft	0.52	0.5	-1.08
8	C.ar	-1.6	1297	H.soft	0.52	0.5	-1.69
26	H.soft	1.39	1863	H.soft	0.49	1.0	-1.83
27	H.soft	1.43	1108	C.ar	-1.44	0.5	-1.23
24	C.ar	1.31	1036	H.soft	0.84	0.5	-0.72
17	C.ar	-0.23	448	H.soft	0.33	0.5	-2.12
17	C.ar	-0.87	1859	C.2	-0.5	0.5	-1.9
25	H.soft	2.31	1064	O.2.am	-7.73	1.0	1.86
6	C.ar	-2.39	232	H.soft	0.88	0.5	-1.15
19	N.ar.no_lp	-1.25	232	H.soft	0.88	0.5	-1.7
18	C.ar	-0.41	230	O.2.am	-1.35	0.5	-1.67
16	C.ar	-0.38	447	H.soft	0.33	0.5	-2.11
14	N.ar	-4.43	1170	H.N	2.84	1.0	0.01

Table S75: 4IH5 protein-ligand complex

Ligand atom number	Ligand atom type	Ligand AIP value	Protein atom number	Protein atom type	Protein AIP value	f	$\Delta\Delta G$ (kJ/mol)
7	N.ar	-2.38	1181	H.soft	0.85	0.5	-1.17
25	H.soft	1.28	1174	C.ar	-1.53	0.5	-1.34
25	H.soft	1.28	1175	C.ar	-1.32	0.5	-1.39
15	C.ar	-1.51	227	H.soft	0.34	0.5	-1.76
16	C.ar	-1.38	1140	H.soft	0.84	0.5	-1.68
1	C.ar	-1.24	1401	H.soft	0.94	0.5	-1.67
5	C.ar	-0.87	1401	H.soft	0.94	0.5	-1.74
1	C.ar	-1.19	492	H.soft	0.97	0.5	-1.66
2	C.ar	-0.34	1139	H.soft	0.65	0.5	-1.97
3	C.ar	-0.96	1139	H.soft	0.65	0.5	-1.9
10	N.ar	-3.56	1142	H.soft	0.81	0.5	-0.21
23	H.soft	0.97	224	H.soft	0.64	1.0	-2.68
22	H.soft	1.1	280	H.soft	0.61	0.5	-1.21
22	H.soft	1.1	273	N.pl3.primary	-0.1	0.5	-1.57
24	H.soft	1.07	230	H.soft	0.47	1.0	-2.71
20	H.N	2.48	1176	O.3.any	-3.71	1.0	-0.14
10	N.ar	-7.17	1404	H.N	2.84	1.0	-0.0

Table S76: 4IH7 protein-ligand complex

Ligand atom number	Ligand atom type	Ligand AIP value	Protein atom number	Protein atom type	Protein AIP value	f	$\Delta\Delta G$ (kJ/mol)
12	C.ar	-1.63	1505	H.soft	0.94	0.5	-1.54
10	C.ar	-1.91	1505	H.soft	0.94	0.5	-1.41
11	C.ar	-1.67	1244	H.soft	0.84	0.5	-1.57
31	H.soft	0.73	233	H.soft	0.34	0.5	-1.77
10	C.ar	-1.47	577	H.soft	0.97	0.5	-1.58
9	C.ar	-1.47	577	H.soft	0.97	0.5	-1.58
1	C.2	-2.46	1246	H.soft	0.81	0.5	-1.12
2	N.2	0.93	1243	H.soft	0.65	0.5	-1.37
20	H.soft	1.34	1243	H.soft	0.65	0.5	-0.86
17	H.soft	1.46	1103	C.2	-1.76	0.5	-1.13
5	C.2	0.43	1278	C.ar	-1.53	0.5	-1.74
8	C.ar	-3.65	1506	H.soft	0.7	0.5	-0.07
28	H.soft	0.65	319	C.2	-2.4	0.5	-1.17
28	H.soft	0.65	318	N.pl3.primary	-0.1	0.5	-1.96
32	H.soft	0.77	230	H.soft	0.64	0.5	-1.54
32	H.soft	0.77	328	H.soft	0.61	0.5	-1.56
25	H.soft	0.76	752	H.soft	0.33	0.5	-1.75
25	H.soft	0.76	747	H.soft	0.33	0.5	-1.75
24	H.soft	1.09	1241	H.soft	0.53	1.0	-2.57
0	O.2.carbonyl	-6.05	1508	H.N	2.84	1.0	-0.03

Table S77: l4IVB protein-ligand complex

Ligand atom number	Ligand atom type	Ligand AIP value	Protein atom number	Protein atom type	Protein AIP value	f	$\Delta\Delta G$ (kJ/mol)
7	N.ar	-1.82	1144	H.soft	0.76	0.5	-1.52
3	C.ar	-1.31	194	H.soft	0.33	0.5	-1.86
4	N.ar.no_lp	0.32	194	H.soft	0.33	0.5	-2.03
28	H.soft	0.74	1050	O.2.am	-1.35	0.5	-1.74
28	H.soft	0.74	1006	C.ar	-1.55	0.5	-1.66
24	H.soft	1.04	1271	C.2	-0.5	0.5	-1.68
5	C.ar	-0.77	199	H.soft	0.33	0.5	-2.05
6	C.ar	-2.0	199	H.soft	0.33	0.5	-1.45
8	C.ar	-2.55	1016	H.soft	0.68	0.5	-1.05
9	N.ar	0.3	1016	H.soft	0.68	0.5	-1.83
10	C.ar	-1.71	600	H.soft	0.49	0.5	-1.63
7	N.ar	-1.46	198	H.soft	0.33	0.5	-1.78
12	C.ar	-1.35	460	H.soft	0.33	0.5	-1.84
4	N.ar.no_lp	-0.42	1571	H.soft	0.34	0.5	-2.1
5	C.ar	-0.85	1571	H.soft	0.34	0.5	-2.02
11	C.ar	-1.18	1574	H.soft	0.33	0.5	-1.91
12	C.ar	-1.32	1574	H.soft	0.33	0.5	-1.85
13	C.ar	-1.26	782	H.soft	0.33	0.5	-1.88
40	H.soft	0.99	186	C.2	-1.76	0.5	-1.46
40	H.soft	0.99	222	N.pl3.am	-0.38	0.5	-1.72
39	H.soft	1.12	227	H.soft	0.88	1.0	-1.87
35	H.soft	1.13	1497	H.soft	0.65	1.0	-2.29
9	N.ar	0.22	1056	H.soft	0.34	0.5	-2.07
10	C.ar	-1.67	1573	H.soft	0.33	0.5	-1.66
14	N.ar.no_lp	-0.83	601	H.soft	0.49	0.5	-1.99
6	C.ar	-2.07	1572	H.soft	0.33	0.5	-1.4
25	H.soft	0.78	1145	H.soft	0.88	1.0	-2.62
9	N.ar	-7.37	1065	H.N	2.84	1.0	0.01
31	H.N	3.15	962	O.2.am	-7.21	1.0	-0.85
27	H.O	3.49	1272	O.2.other	-5.5	1.0	-0.67
2	O.3.alcohol	-4.27	1168	H.N	2.84	1.0	0.03

Table S78: 4IVC protein-ligand complex

Ligand atom number	Ligand atom type	Ligand AIP value	Protein atom number	Protein atom type	Protein AIP value	f	$\Delta\Delta G$ (kJ/mol)
29	H.soft	0.67	1165	O.2.am	-1.35	0.5	-1.77
29	H.soft	0.67	1121	C.ar	-1.55	0.5	-1.68
43	H.soft	1.52	1848	C.2	-0.5	0.5	-1.12
6	C.ar	-2.21	204	H.soft	0.33	0.5	-1.29
8	C.ar	-2.72	1131	H.soft	0.68	0.5	-0.92
9	N.ar	0.13	1124	H.soft	0.64	0.5	-1.94
10	C.ar	-1.91	646	H.soft	0.49	0.5	-1.51
25	H.soft	1.02	1386	C.2	-0.5	0.5	-1.7
6	C.ar	-2.33	1687	H.soft	0.33	0.5	-1.19
7	N.ar	-2.01	1259	H.soft	0.76	0.5	-1.42
3	C.ar	-1.49	199	H.soft	0.33	0.5	-1.77
4	N.ar.no_lp	0.06	199	H.soft	0.33	0.5	-2.13
7	N.ar	-1.48	203	H.soft	0.33	0.5	-1.77
12	C.ar	-1.66	465	H.soft	0.33	0.5	-1.67
4	N.ar.no_lp	-0.68	1686	H.soft	0.34	0.5	-2.06
5	C.ar	-1.04	1686	H.soft	0.34	0.5	-1.96
11	C.ar	-1.38	1689	H.soft	0.33	0.5	-1.82
12	C.ar	-1.52	1689	H.soft	0.33	0.5	-1.75
13	C.ar	-1.48	897	H.soft	0.33	0.5	-1.77
41	H.soft	0.88	191	C.2	-1.76	0.5	-1.51
41	H.soft	0.88	227	N.pl3.am	-0.38	0.5	-1.81
26	H.soft	0.74	1260	H.soft	0.88	1.0	-2.69
39	H.soft	1.05	230	O.2.am	-1.35	0.5	-1.57
40	H.soft	1.09	232	H.soft	0.88	1.0	-1.94
9	N.ar	0.17	1171	H.soft	0.34	0.5	-2.08
10	C.ar	-1.86	1688	H.soft	0.33	0.5	-1.54
13	C.ar	-1.62	1049	H.soft	0.84	0.5	-1.59
14	N.ar.no_lp	-1.08	647	H.soft	0.49	0.5	-1.92
36	H.soft	0.86	1852	H.soft	0.49	0.5	-1.56
36	H.soft	0.86	1612	H.soft	0.65	0.5	-1.44
9	N.ar	-7.65	1180	H.N	2.84	1.0	0.01
28	H.O	3.44	1387	O.2.other	-5.5	1.0	-0.63
32	H.N	3.03	1077	O.2.am	-7.21	1.0	-0.53
2	O.3.alcohol	-4.32	1283	H.N	2.84	1.0	0.02

Table S79: 4IVD protein-ligand complex

Ligand atom number	Ligand atom type	Ligand AIP value	Protein atom number	Protein atom type	Protein AIP value	f	$\Delta\Delta G$ (kJ/mol)
3	C.ar	-1.65	204	H.soft	0.33	0.5	-1.68
4	N.ar.no_lp	-0.16	204	H.soft	0.33	0.5	-2.12
7	N.ar	-2.36	1223	H.soft	0.76	0.5	-1.19
30	H.soft	0.64	1129	O.2.am	-1.35	0.5	-1.78
30	H.soft	0.64	1085	C.ar	-1.55	0.5	-1.69
5	C.ar	-1.19	209	H.soft	0.33	0.5	-1.91
6	C.ar	-2.22	209	H.soft	0.33	0.5	-1.28
8	C.ar	-2.84	1095	H.soft	0.68	0.5	-0.82
9	N.ar	-0.05	1095	H.soft	0.68	0.5	-1.93
10	C.ar	-2.02	610	H.soft	0.49	0.5	-1.44
26	H.soft	0.99	1350	C.2	-0.5	0.5	-1.72
7	N.ar	-1.76	208	H.soft	0.33	0.5	-1.61
12	C.ar	-1.77	470	H.soft	0.33	0.5	-1.6
43	H.soft	0.7	473	H.soft	0.33	0.5	-1.8
4	N.ar.no_lp	-0.95	1650	H.soft	0.34	0.5	-1.99
5	C.ar	-1.22	1650	H.soft	0.34	0.5	-1.89
11	C.ar	-1.54	1653	H.soft	0.33	0.5	-1.74
12	C.ar	-1.66	1653	H.soft	0.33	0.5	-1.67
14	N.ar.no_lp	-1.07	861	H.soft	0.33	0.5	-1.96
41	H.soft	0.97	237	H.soft	0.88	1.0	-2.23
42	H.soft	0.84	196	C.2	-1.76	0.5	-1.53
42	H.soft	0.84	232	N.pl3.am	-0.38	0.5	-1.84
6	C.ar	-2.44	1651	H.soft	0.33	0.5	-1.1
37	H.soft	0.74	1576	H.soft	0.65	1.0	-3.11
9	N.ar	-0.42	1135	H.soft	0.34	0.5	-2.1
10	C.ar	-1.96	1652	H.soft	0.33	0.5	-1.48
27	H.soft	0.66	1224	H.soft	0.88	1.0	-2.83
14	N.ar.no_lp	-1.19	611	H.soft	0.49	0.5	-1.88
13	C.ar	-1.7	1013	H.soft	0.84	0.5	-1.55
44	H.soft	1.13	1812	C.2	-0.5	0.5	-1.59
9	N.ar	-7.82	1144	H.N	2.84	1.0	0.02
33	H.N	2.98	1041	O.2.am	-7.21	1.0	-0.39
29	H.O	3.39	1351	O.2.other	-5.5	1.0	-0.58
2	O.3.alcohol	-4.54	1247	H.N	2.84	1.0	-0.01

Table S80: 4J21 protein-ligand complex

Ligand atom number	Ligand atom type	Ligand AIP value	Protein atom number	Protein atom type	Protein AIP value	f	$\Delta\Delta G$ (kJ/mol)
0	C.ar	-2.77	1155	H.soft	0.64	0.5	-0.87
1	C.ar	-1.15	1257	C.ar	-1.42	0.5	-1.23
2	C.ar	-1.78	1267	H.soft	0.69	0.5	-1.56
5	C.ar	-1.44	1261	C.ar	-1.53	0.5	-0.94
6	C.ar	-1.11	1259	C.ar	-1.52	0.5	-1.18
8	C.ar	-1.02	1260	C.ar	-1.44	0.5	-1.3
9	C.ar	-1.79	1258	C.ar	-1.45	0.5	-0.71
11	N.ar.no_lp	-0.77	1265	H.soft	0.51	0.5	-1.99
12	C.ar	-1.87	1266	H.soft	0.5	0.5	-1.54
18	Cl	-1.46	1407	N.pl3.am	-0.38	0.5	-1.61
18	Cl	-1.9	1267	H.soft	0.69	0.5	-1.49
18	Cl	0.0	1266	H.soft	0.5	0.5	-2.07
18	Cl	0.0	1391	C.2	-1.76	0.5	-1.59
19	C.ar	-1.53	1419	H.soft	0.34	0.5	-1.74
23	H.soft	0.99	1262	C.ar	-1.32	0.5	-1.62
19	C.ar	-1.1	599	H.soft	0.51	0.5	-1.91
28	H.soft	1.14	586	N.pl3.am	-0.38	0.5	-1.57
32	H.soft	0.98	591	C.ar	-1.42	0.5	-1.59
32	H.soft	0.98	592	C.ar	-1.45	0.5	-1.58
6	C.ar	-0.85	868	H.soft	0.51	0.5	-1.98
5	C.ar	-1.09	868	H.soft	0.51	0.5	-1.91
0	C.ar	-2.75	182	N.ar.no_lp	-0.75	0.5	-0.25
11	N.ar.no_lp	-0.88	184	C.ar	-2.05	0.5	-0.88
18	Cl	-0.94	1393	H.soft	0.76	0.5	-1.85
24	H.soft	1.01	862	C.ar	-1.52	0.5	-1.54
24	H.soft	1.01	860	C.ar	-1.42	0.5	-1.57
22	H.soft	1.0	1581	C.2	-0.5	0.5	-1.71
17	C.ar	-1.1	1417	H.soft	0.23	0.5	-1.96
21	H.soft	1.17	593	C.ar	-1.52	0.5	-1.43
18	Cl	-0.85	243	H.soft	0.64	0.5	-1.92
27	H.soft	1.19	244	H.soft	0.54	1.0	-2.31
16	N.pl3.aniline	-0.77	307	H.soft	0.71	0.5	-1.9
17	C.ar	-1.07	272	H.soft	0.34	0.5	-1.95
3	C.ar	-1.19	1420	H.soft	0.34	0.5	-1.91
13	C.ar	-1.02	1420	H.soft	0.34	0.5	-1.97
10	O.2.am	-8.34	225	H.N	2.84	1.0	0.03
26	H.N	2.5	222	O.2.am	-7.73	1.0	1.18

Table S81: 4J3L protein-ligand complex

Ligand atom number	Ligand atom type	Ligand AIP value	Protein atom number	Protein atom type	Protein AIP value	f	$\Delta\Delta G$ (kJ/mol)
2	C.ar	-0.44	332	H.soft	0.71	0.5	-1.93
13	C.ar	-0.35	332	H.soft	0.71	0.5	-1.93
5	C.ar	-0.3	926	H.soft	0.51	0.5	-2.05
22	C.ar	-0.19	926	H.soft	0.51	0.5	-2.04
4	C.ar	-1.03	1477	H.soft	0.34	0.5	-1.97
7	C.ar	-1.39	1324	H.soft	0.5	0.5	-1.79
15	C.ar	-1.35	1316	C.ar	-1.45	0.5	-1.07
16	C.ar	-0.99	1318	C.ar	-1.44	0.5	-1.31
17	C.ar	-0.61	1317	C.ar	-1.52	0.5	-1.47
19	C.ar	-2.18	1213	H.soft	0.64	0.5	-1.32
21	N.ar.no_lp	-0.39	1323	H.soft	0.51	0.5	-2.04
23	C.ar	-0.55	1477	H.soft	0.34	0.5	-2.09
24	Cl	-1.38	1465	N.pl3.am	-0.38	0.5	-1.67
24	Cl	0.0	1449	C.2	-1.76	0.5	-1.59
24	Cl	0.0	1324	H.soft	0.5	0.5	-2.07
24	Cl	-1.46	1328	H.soft	0.7	0.5	-1.71
24	Cl	-1.16	1451	H.soft	0.76	0.5	-1.79
31	H.soft	1.08	1320	C.ar	-1.32	0.5	-1.55
31	H.soft	1.08	1319	C.ar	-1.53	0.5	-1.49
5	C.ar	-0.61	1315	C.ar	-1.42	0.5	-1.54
4	C.ar	-0.53	657	H.soft	0.51	0.5	-2.03
28	H.soft	1.35	644	N.pl3.am	-0.38	0.5	-1.32
29	H.soft	1.15	649	C.ar	-1.42	0.5	-1.48
29	H.soft	1.15	651	C.ar	-1.52	0.5	-1.45
18	C.ar	-0.86	915	C.2	-1.76	0.5	-1.14
19	C.ar	-2.29	916	O.2.am	-1.35	0.5	-0.32
21	N.ar.no_lp	-0.46	209	C.ar	-2.05	0.5	-1.12
32	H.soft	1.11	919	C.ar	-1.45	0.5	-1.5
30	H.soft	1.11	1639	C.2	-0.5	0.5	-1.61
25	C.ar	-2.0	1475	H.soft	0.23	0.5	-1.44
24	Cl	-0.48	268	H.soft	0.64	0.5	-1.97
2	C.ar	-0.79	1478	H.soft	0.34	0.5	-2.04
3	C.ar	-0.45	1478	H.soft	0.34	0.5	-2.1
13	C.ar	-0.56	656	H.soft	1.11	0.5	-1.61
42	H.soft	0.97	1021	H.soft	0.49	1.0	-2.9
41	H.soft	1.31	650	C.ar	-1.45	0.5	-1.34
17	C.ar	-0.6	927	H.soft	0.5	0.5	-2.03
20	O.2.am	-7.75	250	H.N	2.84	1.0	0.02
43	H.N	2.73	247	O.2.am	-7.73	1.0	0.39

Table S82: 4K18 protein-ligand complex

Ligand atom number	Ligand atom type	Ligand AIP value	Protein atom number	Protein atom type	Protein AIP value	f	$\Delta\Delta G$ (kJ/mol)
17	O.2.am	-2.13	1000	H.soft	0.33	0.5	-1.35
34	H.soft	1.74	199	C.2	-1.76	0.5	-0.86
11	C.ar	0.52	1842	H.soft	0.33	0.5	-1.92
12	N.ar	1.46	1842	H.soft	0.33	0.5	-0.93
13	C.ar	-0.72	1837	H.soft	0.22	0.5	-2.08
14	C.ar	-1.81	1866	N.pl3.am	-0.48	0.5	-1.31
26	O.3.any	0.0	282	C.ar	-1.44	0.5	-1.79
26	O.3.any	0.0	283	C.ar	-1.52	0.5	-1.75
35	H.soft	1.99	285	C.ar	-1.55	0.5	-0.56
32	H.soft	1.66	197	N.pl3.am	-0.38	0.5	-0.89
18	N.pl3.am	-1.53	380	H.soft	0.33	0.5	-1.75
19	C.ar	-2.01	380	H.soft	0.33	0.5	-1.44
19	C.ar	-1.66	1687	H.soft	0.33	0.5	-1.67
12	N.ar	1.55	383	H.soft	0.33	0.5	-0.79
11	C.ar	0.32	383	H.soft	0.33	0.5	-2.03
10	N.ar.no_lp	1.33	1875	H.soft	0.49	0.5	-1.0
41	H.soft	0.92	834	H.soft	0.33	1.0	-3.21
21	C.ar	-2.19	174	H.soft	0.33	0.5	-1.31
25	F	-1.64	1222	H.soft	0.33	0.5	-1.68
25	F	-1.64	1077	H.soft	0.34	0.5	-1.68
40	H.soft	0.93	1073	H.soft	0.65	1.0	-2.75
1	C.ar	0.54	1684	H.soft	0.34	0.5	-1.9
20	C.ar	-1.9	1684	H.soft	0.34	0.5	-1.52
31	H.soft	1.54	169	H.soft	0.33	1.0	-1.61
4	C.ar	-0.32	202	H.soft	0.88	0.5	-1.81
24	C.ar	-1.61	829	H.soft	0.23	0.5	-1.7
22	C.ar	-1.53	1686	H.soft	0.33	0.5	-1.75
14	C.ar	-1.59	636	H.soft	0.33	0.5	-1.71
15	N.ar	0.09	636	H.soft	0.33	0.5	-2.12
30	H.soft	1.53	1296	H.soft	0.49	1.0	-1.41
26	O.3.any	-0.77	1871	C.2	-0.5	0.5	-1.94
23	C.ar	-1.99	568	H.soft	0.49	0.5	-1.46
18	N.pl3.am	-1.2	1840	H.soft	0.34	0.5	-1.9
15	N.ar	1.91	1874	H.soft	1.11	0.5	0.62
15	N.ar	-4.64	642	H.N	2.7	1.0	0.01
42	H.O	5.49	1873	O.2.other	-5.5	1.0	-2.31

Table S83: 4K77 protein-ligand complex

Ligand atom number	Ligand atom type	Ligand AIP value	Protein atom number	Protein atom type	Protein AIP value	f	$\Delta\Delta G$ (kJ/mol)
10	N.ar	-1.64	179	H.soft	0.33	0.5	-1.68
9	C.ar	-2.45	174	H.soft	0.33	0.5	-1.09
6	C.ar	-0.35	391	H.soft	0.33	0.5	-2.11
0	C.ar	-0.65	391	H.soft	0.33	0.5	-2.07
11	N.pl3.aniline	-1.15	394	H.soft	0.33	0.5	-1.92
6	C.ar	-0.01	1477	H.soft	0.33	0.5	-2.12
7	C.ar	-0.66	1474	H.soft	0.34	0.5	-2.07
19	H.soft	1.79	925	S.3	-3.5	1.0	-0.64
1	C.ar	-0.25	780	H.soft	0.33	0.5	-2.12
28	H.soft	0.67	1636	C.2	-0.5	0.5	-1.95
3	C.ar	-3.07	1476	H.soft	0.33	0.5	-0.51
31	H.soft	0.68	204	C.2	-1.76	0.5	-1.57
31	H.soft	0.68	220	N.pl3.am	-0.48	0.5	-1.95
24	H.soft	0.72	1640	H.soft	0.49	1.0	-3.38
10	N.ar	-1.53	1475	H.soft	0.33	0.5	-1.75
1	C.ar	-0.53	928	H.soft	0.51	0.5	-2.03
23	H.soft	0.83	207	H.soft	0.88	1.0	-2.52
26	H.soft	0.72	1354	H.soft	0.34	1.0	-3.55
3	C.ar	-2.9	572	H.soft	0.49	0.5	-0.73
20	H.N	3.1	960	O.2.am	-7.21	1.0	-0.72
4	O.2.am	-6.97	1063	H.N	2.84	1.0	-0.01

Table S84: 4KZQ protein-ligand complex

Ligand atom number	Ligand atom type	Ligand AIP value	Protein atom number	Protein atom type	Protein AIP value	f	$\Delta\Delta G$ (kJ/mol)
3	C.ar	-0.91	858	C.2	-1.76	0.5	-1.11
6	C.ar	-1.98	859	O.2.am	-1.35	0.5	-0.62
4	C.ar	-0.55	869	H.soft	0.51	0.5	-2.03
8	C.ar	-0.63	1258	C.ar	-1.42	0.5	-1.53
8	C.ar	-0.42	869	H.soft	0.51	0.5	-2.04
9	O.2.one_lp	0.0	1261	C.ar	-1.44	0.5	-1.79
15	C.ar	-0.89	1259	C.ar	-1.45	0.5	-1.37
26	H.soft	1.16	593	C.ar	-1.45	0.5	-1.46
26	H.soft	1.16	592	C.ar	-1.42	0.5	-1.47
0	C.ar	-0.97	874	H.soft	0.7	0.5	-1.87
3	C.ar	-1.26	1260	C.ar	-1.52	0.5	-1.08
4	C.ar	-0.59	1262	C.ar	-1.53	0.5	-1.47
5	C.ar	-0.99	1263	C.ar	-1.32	0.5	-1.4
6	C.ar	-2.16	1156	H.soft	0.64	0.5	-1.34
2	C.ar	-1.11	910	C.2	-1.76	0.5	-0.99
15	C.ar	-0.79	600	H.soft	0.51	0.5	-1.99
14	C.ar	-0.79	600	H.soft	0.51	0.5	-1.99
20	H.soft	0.86	957	N.pl3.am	-0.38	0.5	-1.83
0	C.ar	-1.09	1121	H.soft	0.33	0.5	-1.95
7	C.ar	-0.79	224	C.ar	-2.05	0.5	-0.93
7	C.ar	-1.34	262	O.2.am	-7.73	0.5	6.99
11	C.ar	-0.78	1267	H.soft	0.5	0.5	-2.0
22	H.soft	1.25	262	O.2.am	-1.35	0.5	-1.41
22	H.soft	1.25	259	N.pl3.am	-0.38	0.5	-1.45
23	H.soft	1.35	284	H.soft	0.54	0.5	-0.94
23	H.soft	1.35	262	O.2.am	-7.21	0.5	2.5
25	H.soft	1.15	594	C.ar	-1.52	0.5	-1.45
25	H.soft	1.15	1421	H.soft	0.34	0.5	-1.34
1	C.ar	-1.11	911	O.2.am	-1.35	0.5	-1.3
19	H.soft	1.06	1593	H.soft	0.34	0.5	-1.45
19	H.soft	1.06	964	H.soft	0.49	0.5	-1.35
1	C.ar	-1.26	1587	C.2	-0.5	0.5	-1.69
2	C.ar	-2.16	1120	H.soft	0.24	0.5	-1.32
5	C.ar	-0.91	862	C.ar	-1.45	0.5	-1.36
14	C.ar	-0.91	1419	H.soft	0.23	0.5	-2.03
13	C.ar	-0.67	1419	H.soft	0.23	0.5	-2.09
24	H.soft	1.58	312	H.soft	0.34	1.0	-1.46
16	O.2.carbonyl	-7.02	265	H.N	2.84	1.0	-0.01

Table S85: 4KZU protein-ligand complex

Ligand atom number	Ligand atom type	Ligand AIP value	Protein atom number	Protein atom type	Protein AIP value	f	$\Delta\Delta G$ (kJ/mol)
0	C.ar	-0.24	224	N.ar.no_lp	-0.75	0.5	-2.02
1	C.ar	-1.54	894	O.2.am	-1.35	0.5	-1.0
3	C.ar	-0.56	893	C.2	-1.76	0.5	-1.31
17	C.ar	-0.27	1302	H.soft	0.5	0.5	-2.05
18	H.soft	1.41	226	C.ar	-2.05	0.5	-1.09
18	H.soft	1.41	264	O.2.am	-1.35	0.5	-1.26
26	H.soft	1.32	264	O.2.am	-7.21	1.0	5.12
1	C.ar	-1.71	1191	H.soft	0.64	0.5	-1.61
3	C.ar	-0.84	1295	C.ar	-1.52	0.5	-1.34
7	C.ar	-0.64	1298	C.ar	-1.32	0.5	-1.59
8	C.ar	-0.28	1297	C.ar	-1.53	0.5	-1.61
9	O.2.one_lp	-0.6	1296	C.ar	-1.44	0.5	-1.53
9	O.2.one_lp	-0.21	628	C.ar	-1.45	0.5	-1.69
9	O.2.one_lp	-0.42	628	C.ar	-1.45	0.5	-1.6
10	C.ar	-0.05	1293	C.ar	-1.42	0.5	-1.76
11	C.ar	-0.08	1294	C.ar	-1.45	0.5	-1.73
12	C.ar	-0.25	1306	H.soft	0.7	0.5	-1.94
23	H.soft	1.49	627	C.ar	-1.42	0.5	-1.17
12	C.ar	-0.3	635	H.soft	0.51	0.5	-2.05
4	C.ar	-0.78	945	C.2	-1.76	0.5	-1.19
6	C.ar	-0.75	1156	H.soft	0.33	0.5	-2.05
5	C.ar	-0.78	905	H.soft	0.5	0.5	-2.0
6	C.ar	-0.66	909	H.soft	0.7	0.5	-1.92
19	H.soft	0.97	992	N.pl3.am	-0.38	0.5	-1.74
24	H.soft	1.38	629	C.ar	-1.52	0.5	-1.26
24	H.soft	1.38	1404	H.soft	0.34	0.5	-1.04
4	C.ar	-1.48	1155	H.soft	0.24	0.5	-1.78
5	C.ar	-0.91	1570	C.2	-0.5	0.5	-1.88
10	C.ar	-0.01	904	H.soft	0.51	0.5	-2.03
8	C.ar	-0.2	904	H.soft	0.51	0.5	-2.04
7	C.ar	-0.58	897	C.ar	-1.45	0.5	-1.53
0	C.ar	-1.11	264	O.2.am	-7.73	0.5	6.86
25	H.soft	1.36	314	H.soft	0.34	1.0	-2.13
20	H.soft	1.16	999	H.soft	0.49	0.5	-1.23
20	H.soft	1.16	1576	H.soft	0.34	0.5	-1.33
13	C.ar	-0.29	634	H.soft	1.11	0.5	-1.59
2	O.2.carbonyl	-6.36	267	H.N	2.84	1.0	-0.03

Table S86: 4LLX protein-ligand complex

Ligand atom number	Ligand atom type	Ligand AIP value	Protein atom number	Protein atom type	Protein AIP value	f	$\Delta\Delta G$ (kJ/mol)
3	C.ar	-1.91	722	C.ar	-1.44	0.5	-0.62
5	N.ar	-0.78	724	C.ar	-1.53	0.5	-1.37
6	C.ar	-1.8	726	C.ar	-1.56	0.5	-0.62
14	H.soft	0.87	723	C.ar	-1.52	0.5	-1.62
1	C.ar	-1.81	727	C.ar	-1.56	0.5	-0.61
2	C.ar	-1.47	725	C.ar	-1.55	0.5	-0.9
1	C.ar	-1.82	475	H.soft	0.34	0.5	-1.57
6	C.ar	-1.85	479	H.soft	0.33	0.5	-1.55
8	N.ar	-0.81	475	H.soft	0.34	0.5	-2.03
13	H.soft	0.87	666	N.pl3.am	-0.26	0.5	-1.81
15	H.soft	0.68	562	C.ar	-1.56	0.5	-1.67
15	H.soft	0.68	568	H.soft	0.71	0.5	-1.56
12	H.soft	0.91	569	H.soft	0.69	1.0	-2.72
2	C.ar	-1.47	569	H.soft	0.69	0.5	-1.71
10	H.soft	0.8	265	H.soft	0.34	1.0	-3.42
16	H.N	2.59	665	O.2.am	-7.46	1.0	0.79
5	N.ar	-7.59	672	H.N	2.7	1.0	0.46

Table S87: 4LZS protein-ligand complex

Ligand atom number	Ligand atom type	Ligand AIP value	Protein atom number	Protein atom type	Protein AIP value	f	$\Delta\Delta G$ (kJ/mol)
28	H.soft	0.92	281	N.pl3.am	-0.26	0.5	-1.77
1	C.ar	-1.28	1226	H.soft	0.34	0.5	-1.87
5	C.2	-2.07	167	H.soft	0.47	0.5	-1.4
23	H.soft	0.57	1226	H.soft	0.34	0.5	-1.89
3	C.ar	-0.5	357	H.soft	0.33	0.5	-2.1
10	N.ar.no_lp	-0.16	357	H.soft	0.33	0.5	-2.12
18	H.soft	0.92	1022	C.ar	-1.52	0.5	-1.59
18	H.soft	0.92	1030	H.soft	0.69	0.5	-1.35
4	C.2	-1.69	1224	H.soft	0.23	0.5	-1.65
0	C.ar	-1.28	1224	H.soft	0.23	0.5	-1.88
15	H.soft	0.82	189	C.ar	-1.52	0.5	-1.64
5	C.2	-1.77	426	H.soft	0.33	0.5	-1.6
24	H.soft	0.21	424	H.soft	0.33	1.0	-4.15
11	N.pl3.am	-0.48	161	H.soft	0.64	0.5	-1.97
25	H.soft	0.58	500	H.soft	0.33	1.0	-3.77
3	C.ar	-0.98	1227	H.soft	0.34	0.5	-1.99
16	H.soft	0.91	191	C.ar	-1.55	0.5	-1.59
22	H.soft	0.42	1230	H.soft	0.33	1.0	-3.96
29	H.soft	0.91	423	H.soft	0.34	1.0	-3.22
1	C.ar	-1.05	425	H.soft	0.33	0.5	-1.96
27	H.N	3.79	157	O.2.am	-8.69	1.0	-3.92
12	O.2.am	-7.14	1078	H.N	2.62	1.0	0.61
26	H.N	4.16	157	O.2.am	-8.39	1.0	-5.04

Table S88: 4M0Y protein-ligand complex

Ligand atom number	Ligand atom type	Ligand AIP value	Protein atom number	Protein atom type	Protein AIP value	f	$\Delta\Delta G$ (kJ/mol)
21	N.pl3.am	-2.06	1058	N.pl3.am	-0.38	0.5	-1.15
8	C.ar	-1.01	1335	H.soft	0.33	0.5	-1.98
10	N.ar.no_lp	-0.16	1335	H.soft	0.33	0.5	-2.12
28	H.soft	1.05	1332	H.soft	0.34	0.5	-1.46
3	C.ar	-1.05	395	H.soft	0.33	0.5	-1.96
4	C.ar	-0.99	395	H.soft	0.33	0.5	-1.98
9	C.ar	-1.64	668	H.soft	0.35	0.5	-1.68
10	N.ar.no_lp	-1.12	392	H.soft	0.33	0.5	-1.94
19	C.2	-2.45	150	H.soft	0.33	0.5	-1.09
0	C.ar	-1.4	194	C.2	-1.76	0.5	-0.78
22	H.soft	1.01	192	N.pl3.am	-0.38	0.5	-1.7
12	C.ar	-0.83	1336	H.soft	0.34	0.5	-2.03
18	N.pl3.am	-0.84	1062	H.soft	0.76	0.5	-1.87
19	C.2	-1.95	1062	H.soft	0.76	0.5	-1.45
17	N.pl3.am	-0.86	598	H.soft	0.49	0.5	-1.98
21	N.pl3.am	-0.48	897	C.ar	-1.55	0.5	-1.51
25	H.soft	1.09	222	H.soft	0.76	1.0	-2.19
1	C.ar	-1.46	390	H.soft	0.23	0.5	-1.79
23	H.soft	0.96	176	C.2	-1.76	0.5	-1.47
15	C.2	-1.29	947	H.soft	0.51	0.5	-1.84
16	O.2.am	-6.37	954	H.N	2.84	1.0	-0.03
30	H.N	2.33	853	O.2.am	-7.21	1.0	1.5
34	H.N	2.53	941	O.2.am	-7.21	1.0	0.89
32	H.N	2.47	941	O.2.am	-7.73	1.0	1.28

Table S89: 4M0Z protein-ligand complex

Ligand atom number	Ligand atom type	Ligand AIP value	Protein atom number	Protein atom type	Protein AIP value	f	$\Delta\Delta G$ (kJ/mol)
14	C.ar	-1.32	652	H.soft	0.81	0.5	-1.72
20	C.ar	-1.39	387	C.ar	-1.56	0.5	-0.95
17	C.ar	-0.95	385	C.ar	-1.55	0.5	-1.26
18	C.ar	-1.32	383	C.ar	-1.52	0.5	-1.03
4	C.ar	-2.08	1735	C.ar	-1.55	0.5	-0.37
5	N.pl3.am	-0.96	1737	C.ar	-1.56	0.5	-1.24
6	C.2	-2.36	1736	C.ar	-1.56	0.5	-0.09
15	C.ar	-1.31	649	H.soft	0.65	0.5	-1.79
17	C.ar	-0.95	649	H.soft	0.65	0.5	-1.9
0	N.ar.no_lp	-0.07	987	H.soft	0.34	0.5	-2.12
21	C.ar	-1.28	987	H.soft	0.34	0.5	-1.87
7	N.pl3.am	-1.47	1673	H.soft	0.64	0.5	-1.73
2	C.ar	-0.71	985	H.soft	0.33	0.5	-2.06
9	C.2	-1.67	985	H.soft	0.33	0.5	-1.66
1	N.ar	-4.47	647	H.soft	0.53	1.0	1.86
1	N.ar	0.14	1744	H.soft	0.7	0.5	-1.89
2	C.ar	-1.1	1744	H.soft	0.7	0.5	-1.84
13	C.ar	-1.26	650	H.soft	0.84	0.5	-1.72
16	C.ar	-1.37	647	H.soft	0.53	0.5	-1.8
20	C.ar	-1.12	547	H.soft	0.64	0.5	-1.86
5	N.pl3.am	-1.31	975	N.pl3.am	-0.38	0.5	-1.71
6	C.2	-2.35	1204	H.soft	0.7	0.5	-1.2
30	H.soft	1.08	1733	C.ar	-1.52	0.5	-1.5
30	H.soft	1.08	1730	O.2.am	-1.35	0.5	-1.55
10	N.pl3.am	-0.47	683	H.soft	1.11	0.5	-1.61
10	N.pl3.am	-1.41	765	H.soft	0.34	0.5	-1.81
19	C.ar	-1.3	542	N.pl3.am	-0.38	0.5	-1.71
19	C.ar	-1.33	386	C.ar	-1.56	0.5	-1.0
34	H.soft	1.0	380	O.2.am	-1.35	0.5	-1.6
34	H.soft	1.0	549	H.soft	0.49	0.5	-1.42
32	H.soft	1.11	1932	H.soft	0.69	1.0	-2.27
33	H.soft	1.15	513	H.soft	0.34	0.5	-1.34
33	H.soft	1.15	1926	C.ar	-1.56	0.5	-1.44
18	C.ar	-1.29	505	C.2	-2.23	0.5	-0.43
38	H.soft	1.21	675	N.pl3.am	-0.38	0.5	-1.49
31	H.soft	1.05	1781	H.soft	0.88	0.5	-1.02
31	H.soft	1.05	1778	C.2	-1.76	0.5	-1.43
9	C.2	-1.25	1743	H.soft	0.69	0.5	-1.79
37	H.soft	0.98	986	H.soft	0.19	1.0	-3.24
14	C.ar	-1.29	1931	H.soft	0.71	0.5	-1.77
7	N.pl3.am	-1.2	1205	H.soft	0.68	0.5	-1.81
26	H.N	2.55	1672	O.3.alcohol	-5.37	1.0	0.24
27	H.N	2.75	904	O.2.am	-7.73	1.0	0.32
11	O.2.am	-4.82	993	H.N	2.84	1.0	-0.03
25	H.N	2.53	904	O.2.am	-7.21	1.0	0.89
[illegible]	[illegible]	[illegible]	[illegible]	[illegible]	[illegible]	[illegible]	[illegible]

Table S90: 4MGD protein-ligand complex

Ligand atom number	Ligand atom type	Ligand AIP value	Protein atom number	Protein atom type	Protein AIP value	f	$\Delta\Delta G$ (kJ/mol)
2	C.ar	-0.73	806	H.soft	0.33	0.5	-2.06
4	C.ar	-1.03	798	C.2	-1.76	0.5	-1.04
9	Cl	-1.54	1171	H.soft	0.7	0.5	-1.68
6	C.ar	-1.64	1171	H.soft	0.7	0.5	-1.63
15	C.ar	-1.27	298	H.soft	1.11	0.5	-1.54
13	C.ar	-0.74	1620	H.soft	0.34	0.5	-2.05
14	C.ar	-0.78	1620	H.soft	0.34	0.5	-2.04
0	C.ar	-1.02	403	H.soft	0.49	0.5	-1.94
12	C.ar	-1.79	403	H.soft	0.49	0.5	-1.59
1	C.ar	-1.66	1172	H.soft	0.68	0.5	-1.62
4	C.ar	-1.68	942	H.soft	0.33	0.5	-1.66
5	C.ar	-1.69	942	H.soft	0.33	0.5	-1.65
9	Cl	0.0	268	H.soft	0.33	1.0	-4.29
0	C.ar	-1.65	263	H.soft	0.33	0.5	-1.68
9	Cl	0.52	263	H.soft	0.33	0.5	-1.92
18	C.ar	-1.59	255	C.2	-1.76	0.5	-0.63
19	H.soft	0.96	256	O.2.am	-1.35	0.5	-1.63
24	H.soft	1.16	697	H.soft	0.33	1.0	-2.67
9	Cl	-1.2	1270	S.3	-3.5	0.5	0.95
11	Cl	0.66	845	H.soft	0.65	0.5	-1.62
28	H.soft	1.03	291	N.pl3.am	-0.38	0.5	-1.68
14	C.ar	-1.57	1822	H.soft	0.34	0.5	-1.72
9	Cl	-1.37	1355	H.soft	0.71	0.5	-1.74
25	H.soft	0.97	696	H.soft	0.33	1.0	-3.11
22	H.soft	0.98	937	H.soft	0.34	1.0	-3.07
27	H.O	4.12	296	O.3.alcohol	-5.34	1.0	-1.02
21	H.O	4.05	513	O.2.other	-5.5	1.0	-1.17
3	O.3.any	-3.79	1020	H.N	2.7	1.0	0.01

Table S91: 4QAC protein-ligand complex

Ligand atom number	Ligand atom type	Ligand AIP value	Protein atom number	Protein atom type	Protein AIP value	f	$\Delta\Delta G$ (kJ/mol)
15	C.ar	-0.85	882	C.ar	-1.32	0.5	-1.48
9	N.ar	-0.78	219	C.ar	-1.44	0.5	-1.44
9	N.ar	-6.13	221	C.ar	-1.32	0.5	4.67
7	C.ar	-0.82	459	C.ar	-1.99	0.5	-0.97
10	C.ar	-2.64	457	C.ar	-1.98	0.5	0.56
12	N.ar	-0.71	461	H.soft	1.11	0.5	-1.61
6	N.pl3.aniline	-1.9	698	C.ar	-1.32	0.5	-0.72
7	C.ar	-1.68	695	C.ar	-1.52	0.5	-0.75
9	N.ar	0.28	697	C.ar	-1.53	0.5	-1.75
34	H.soft	0.93	1221	C.ar	-1.99	0.5	-1.38
36	H.soft	1.17	1222	C.ar	-1.96	0.5	-1.28
36	H.soft	1.17	1220	C.ar	-1.87	0.5	-1.32
42	H.soft	1.11	454	C.ar	-1.82	0.5	-1.37
42	H.soft	1.11	456	C.ar	-1.67	0.5	-1.43
14	C.ar	-0.61	450	O.2.am	-7.21	0.5	5.82
22	C.ar	-0.82	514	H.soft	1.11	0.5	-1.61
23	C.ar	-0.81	450	O.2.am	-1.35	0.5	-1.48
27	H.soft	0.8	696	C.ar	-1.44	0.5	-1.68
35	H.soft	1.16	694	C.ar	-1.45	0.5	-1.46
19	F	-0.15	1948	N.pl3.am	-0.38	0.5	-2.14
19	F	-0.15	1960	H.soft	0.65	0.5	-1.96
10	C.ar	-0.85	877	C.ar	-1.42	0.5	-1.41
12	N.ar	-1.03	879	C.ar	-1.52	0.5	-1.23
8	C.ar	-0.83	460	C.ar	-1.96	0.5	-0.99
32	H.soft	0.75	460	C.ar	-1.96	0.5	-1.45
25	H.soft	0.68	217	C.ar	-1.45	0.5	-1.72
25	H.soft	0.68	468	H.soft	0.48	0.5	-1.73
17	C.ar	-1.09	1228	H.soft	0.81	0.5	-1.78
22	C.ar	-1.25	1957	H.soft	0.51	0.5	-1.85
28	H.soft	0.71	2132	H.soft	0.5	1.0	-3.38
33	H.soft	0.81	2123	C.ar	-1.42	0.5	-1.68
33	H.soft	0.81	1219	C.ar	-1.98	0.5	-1.42
24	H.soft	0.62	220	C.ar	-1.53	0.5	-1.7
24	H.soft	0.62	218	C.ar	-1.52	0.5	-1.71
39	H.soft	1.0	880	C.ar	-1.44	0.5	-1.57
21	F	-0.08	517	H.soft	0.34	0.5	-2.12
21	F	-0.08	1681	H.soft	0.34	0.5	-2.12
26	H.soft	0.9	225	H.soft	0.5	1.0	-3.03
41	H.soft	1.11	453	C.ar	-1.53	0.5	-1.47
41	H.soft	1.11	455	N.ar.no_lp	-1.17	0.5	-1.56
29	H.soft	0.77	1102	H.soft	0.34	1.0	-3.47
38	H.N	1.77	424	O.2.am	-7.21	1.0	3.37
37	H.N	1.99	222	O.3.any	-3.71	1.0	-0.4

Table S92: 4QD6 protein-ligand complex

Ligand atom number	Ligand atom type	Ligand AIP value	Protein atom number	Protein atom type	Protein AIP value	f	$\Delta\Delta G$ (kJ/mol)
20	N.ar.no_lp	-1.6	229	H.soft	0.49	0.5	-1.69
21	N.ar	-3.33	698	H.soft	0.68	0.5	-0.38
5	C.ar	-1.25	853	H.soft	0.76	0.5	-1.76
16	N.pl3.aniline	-1.03	853	H.soft	0.76	0.5	-1.83
19	C.ar	-1.57	1174	H.soft	0.33	0.5	-1.72
20	N.ar.no_lp	-1.61	740	H.soft	0.65	0.5	-1.66
0	C.ar	-1.69	849	N.pl3.am	-0.38	0.5	-1.45
17	C.ar	-1.57	738	H.soft	0.51	0.5	-1.71
18	C.ar	-1.75	1173	H.soft	0.33	0.5	-1.62
21	N.ar	-3.15	738	H.soft	0.51	0.5	-0.49
31	C.ar	-1.43	597	C.ar	-1.44	0.5	-1.01
58	H.soft	0.72	599	C.ar	-1.53	0.5	-1.67
58	H.soft	0.72	601	C.ar	-1.56	0.5	-1.66
1	C.ar	-1.74	854	H.soft	0.88	0.5	-1.52
2	C.ar	-1.31	854	H.soft	0.88	0.5	-1.68
33	C.ar	-1.43	297	H.soft	0.33	0.5	-1.8
30	C.ar	-1.53	367	H.soft	0.33	0.5	-1.75
31	C.ar	-1.55	1288	H.soft	0.54	0.5	-1.71
16	N.pl3.aniline	-1.21	688	C.ar	-1.55	0.5	-1.09
35	H.soft	1.21	690	C.ar	-1.56	0.5	-1.39
7	C.ar	-0.43	696	H.soft	0.69	0.5	-1.95
12	C.ar	-0.6	696	H.soft	0.69	0.5	-1.94
57	H.soft	1.09	605	H.soft	0.57	1.0	-2.51
29	C.ar	-1.32	1175	H.soft	0.33	0.5	-1.85
21	N.ar	-6.49	745	H.N	2.84	1.0	-0.02
45	H.N	3.38	644	O.2.am	-7.21	1.0	-1.47
43	H.N	2.42	732	O.2.am	-7.73	1.0	1.46

Table S93: 4RFM protein-ligand complex

Ligand atom number	Ligand atom type	Ligand AIP value	Protein atom number	Protein atom type	Protein AIP value	f	$\Delta\Delta G$ (kJ/mol)
14	C.ar	-2.26	1374	N.pl3.am	-0.38	0.5	-0.97
12	N.ar	-2.46	226	H.soft	0.22	0.5	-1.06
43	H.soft	0.95	255	N.pl3.am	-0.38	0.5	-1.75
62	H.soft	1.04	363	C.ar	-1.44	0.5	-1.55
62	H.soft	1.04	364	C.ar	-1.52	0.5	-1.52
9	N.pl3.am	0.06	231	H.soft	0.33	0.5	-2.13
10	C.ar	-0.97	1222	H.soft	0.7	0.5	-1.87
3	C.ar	-1.32	1748	H.soft	0.33	0.5	-1.85
5	N.ar.no_lp	-0.85	1263	H.soft	0.51	0.5	-1.98
6	C.ar	-0.68	1747	H.soft	0.33	0.5	-2.07
10	C.ar	-2.67	1378	H.soft	0.76	0.5	-0.96
9	N.pl3.am	-1.47	1378	H.soft	0.76	0.5	-1.68
30	C.ar	-1.19	473	H.soft	0.33	0.5	-1.91
61	H.soft	0.68	476	H.soft	0.33	0.5	-1.81
4	N.ar	-3.13	1254	N.pl3.am	-0.38	0.5	-0.04
58	H.soft	1.2	145	N.pl3.am	-0.26	0.5	-1.49
58	H.soft	1.2	143	C.2	-1.35	0.5	-1.45
39	H.soft	0.75	944	H.soft	0.33	1.0	-3.52
38	H.soft	0.83	1122	C.ar	-1.44	0.5	-1.67
38	H.soft	0.83	1124	C.ar	-1.53	0.5	-1.63
60	H.soft	0.58	1746	H.soft	0.34	0.5	-1.88
60	H.soft	0.58	365	C.ar	-1.53	0.5	-1.71
25	C.ar	-0.4	1221	H.soft	0.69	0.5	-1.95
26	C.ar	-0.59	1221	H.soft	0.69	0.5	-1.94
40	H.soft	0.89	731	H.soft	0.49	0.5	-1.53
40	H.soft	0.89	1129	H.soft	0.57	0.5	-1.48
14	C.ar	-0.73	1213	C.ar	-1.55	0.5	-1.38
41	H.N	4.07	1211	C.ar	-1.52	0.5	2.73
4	N.ar	-3.03	1216	H.soft	0.64	0.5	-0.64
5	N.ar.no_lp	-1.06	730	H.soft	0.49	0.5	-1.92
44	H.soft	1.84	1294	C.2	-2.23	0.5	-0.69
44	H.soft	1.84	1215	C.ar	-1.56	0.5	-0.76
27	C.ar	-0.6	553	H.soft	0.33	0.5	-2.08
28	C.ar	-0.58	233	H.soft	0.33	0.5	-2.09
34	F	-0.96	472	H.soft	0.33	0.5	-1.99
34	F	-0.96	729	H.soft	0.49	0.5	-1.95
42	H.N	3.81	1257	O.2.am	-7.73	1.0	-3.1
4	N.ar	-5.89	1270	H.N	2.84	1.0	-0.04
12	N.ar	-5.55	261	H.N	2.84	1.0	-0.04

Table S94: 4TWP protein-ligand complex

Ligand atom number	Ligand atom type	Ligand AIP value	Protein atom number	Protein atom type	Protein AIP value	f	$\Delta\Delta G$ (kJ/mol)
17	N.ar.no_lp	-1.05	646	H.soft	0.49	0.5	-1.93
6	C.ar	-0.82	357	H.soft	0.69	0.5	-1.91
37	H.soft	1.08	348	C.ar	-1.45	0.5	-1.52
41	H.soft	1.05	350	C.ar	-1.44	0.5	-1.54
41	H.soft	1.05	352	C.ar	-1.32	0.5	-1.58
4	C.ar	-0.68	356	H.soft	0.5	0.5	-2.01
8	C.ar	-1.11	356	H.soft	0.5	0.5	-1.91
22	C.ar	-1.19	1296	H.soft	0.76	0.5	-1.78
21	C.2	-1.1	1296	H.soft	0.76	0.5	-1.81
26	C.ar	-0.49	1242	C.2	-1.76	0.5	-1.35
27	C.ar	-0.49	1292	N.pl3.am	-0.38	0.5	-2.07
11	C.ar	-2.01	461	H.soft	0.33	0.5	-1.44
12	C.ar	-1.9	461	H.soft	0.33	0.5	-1.52
18	C.ar	-0.9	224	H.soft	0.33	0.5	-2.01
21	C.2	-1.24	219	H.soft	0.33	0.5	-1.89
22	C.ar	-1.37	223	H.soft	0.33	0.5	-1.83
35	H.soft	0.98	1674	H.soft	0.33	1.0	-3.09
40	H.soft	1.42	1130	C.ar	-1.55	0.5	-1.22
40	H.soft	1.42	1128	C.ar	-1.52	0.5	-1.22
45	H.soft	1.4	1174	O.2.am	-1.35	0.5	-1.27
45	H.soft	1.4	1132	C.ar	-1.56	0.5	-1.23
4	C.ar	-1.02	1837	H.soft	0.49	0.5	-1.94
5	C.ar	-1.0	1833	C.2	-0.5	0.5	-1.83
36	H.soft	0.87	347	C.ar	-1.42	0.5	-1.65
36	H.soft	0.87	349	C.ar	-1.52	0.5	-1.62
15	C.ar	-1.94	1673	H.soft	0.33	0.5	-1.49
17	N.ar.no_lp	-1.15	1187	H.N	2.84	0.5	0.89
18	C.ar	-0.74	1673	H.soft	0.33	0.5	-2.05
38	H.soft	0.8	1061	H.soft	0.33	1.0	-3.43
13	C.ar	-1.21	1671	H.soft	0.34	0.5	-1.9
23	N.ar	-2.91	359	H.soft	0.94	0.5	-0.78
15	C.ar	-2.14	645	H.soft	0.49	0.5	-1.36
7	C.ar	-1.13	1602	H.soft	0.65	0.5	-1.85
16	N.ar	-3.27	647	H.soft	0.49	0.5	-0.37
23	N.ar	-6.62	361	H.O	3.66	1.0	-1.74
0	O.2.am	-7.6	715	H.N	2.7	1.0	0.46
39	H.N	3.5	1086	O.2.am	-7.21	1.0	-1.79

Table S95: 4W9C protein-ligand complex

Ligand atom number	Ligand atom type	Ligand AIP value	Protein atom number	Protein atom type	Protein AIP value	f	$\Delta\Delta G$ (kJ/mol)
34	H.soft	0.55	568	C.ar	-1.99	0.5	-1.46
26	N.ar	-3.16	873	H.soft	0.48	0.5	-0.47
20	C.ar	-0.85	1092	H.soft	0.22	0.5	-2.05
21	C.ar	-0.86	1092	H.soft	0.22	0.5	-2.04
21	C.ar	-1.28	816	C.ar	-1.52	0.5	-1.06
22	C.ar	-1.08	824	H.soft	0.69	0.5	-1.85
23	C.ar	-0.44	870	H.soft	0.34	0.5	-2.1
24	O.2.one_lp	-0.47	870	H.soft	0.34	0.5	-2.1
24	O.2.one_lp	-0.91	823	H.soft	0.5	1.0	-3.93
30	H.soft	0.43	576	H.soft	0.54	1.0	-3.71
37	H.soft	0.8	567	C.ar	-1.87	0.5	-1.48
37	H.soft	0.8	569	C.ar	-1.96	0.5	-1.43
40	H.soft	1.1	565	C.ar	-1.67	0.5	-1.43
40	H.soft	1.1	1327	H.soft	1.27	0.5	-0.47
13	C.2	-1.48	825	H.soft	0.85	0.5	-1.64
17	C.ar	-1.33	825	H.soft	0.85	0.5	-1.69
20	C.ar	-1.35	818	C.ar	-1.53	0.5	-1.0
43	H.soft	1.37	819	C.ar	-1.32	0.5	-1.3
41	H.soft	1.33	1405	C.ar	-1.87	0.5	-1.21
41	H.soft	1.33	561	C.ar	-1.57	0.5	-1.29
26	N.ar	-3.04	993	H.soft	0.61	0.5	-0.63
27	C.ar	-1.3	993	H.soft	0.61	0.5	-1.8
39	H.soft	0.98	566	C.ar	-1.98	0.5	-1.36
39	H.soft	0.98	563	C.ar	-1.82	0.5	-1.44
24	O.2.one_lp	-0.83	1099	H.soft	0.33	0.5	-2.03
44	H.soft	1.02	1413	H.soft	0.81	1.0	-2.26
22	C.ar	-0.77	1100	H.soft	0.33	0.5	-2.05
23	C.ar	-0.46	1100	H.soft	0.33	0.5	-2.1
52	H.soft	1.13	1407	C.ar	-1.96	0.5	-1.3
5	C.2	-4.26	1207	H.soft	0.94	0.5	0.4
53	H.soft	1.78	538	H.soft	0.34	1.0	-0.79
42	H.O	3.76	1169	O.3.alcohol	-5.37	1.0	-0.79
46	H.N	2.74	1124	O.2.am	-7.73	1.0	0.35
26	N.ar	-6.2	995	H.N	2.7	1.0	0.22
10	O.3.alcohol	-3.44	1328	H.N	3.44	1.0	0.92

Table S96: 4W9H protein-ligand complex

Ligand atom number	Ligand atom type	Ligand AIP value	Protein atom number	Protein atom type	Protein AIP value	f	$\Delta\Delta G$ (kJ/mol)
17	C.2	-1.42	895	H.soft	0.85	0.5	-1.66
21	C.ar	-0.54	895	H.soft	0.85	0.5	-1.84
51	H.soft	1.39	889	C.ar	-1.32	0.5	-1.28
51	H.soft	1.39	888	C.ar	-1.53	0.5	-1.25
26	C.ar	-0.65	894	H.soft	0.69	0.5	-1.93
27	C.ar	-0.65	940	H.soft	0.34	0.5	-2.07
28	S.2.phene	-0.58	893	H.soft	0.5	1.0	-4.06
29	C.ar	-1.74	934	O.2.am	-8.69	0.5	8.62
30	N.ar	-3.08	943	H.soft	0.48	0.5	-0.55
61	H.soft	1.54	934	O.2.am	-1.8	0.5	-1.05
1	C.2	-2.27	1295	C.ar	-1.45	0.5	-0.27
3	N.pl3.am	-2.09	1297	C.ar	-1.44	0.5	-0.45
9	C.2	-3.6	1306	H.soft	0.94	0.5	-0.21
22	C.ar	-1.36	1191	H.soft	0.22	0.5	-1.84
23	C.ar	-1.25	1191	H.soft	0.22	0.5	-1.89
34	H.soft	0.8	275	H.soft	0.65	0.5	-1.5
34	H.soft	0.8	1298	C.ar	-1.53	0.5	-1.64
28	S.2.phene	-0.16	1198	H.soft	0.33	0.5	-2.12
28	S.2.phene	-0.16	451	C.ar	-1.53	0.5	-1.65
28	S.2.phene	0.0	575	H.soft	0.34	0.5	-2.14
49	H.soft	1.28	631	C.ar	-1.57	0.5	-1.33
49	H.soft	1.28	1504	C.ar	-1.87	0.5	-1.24
40	H.soft	0.62	769	C.ar	-1.56	0.5	-1.69
40	H.soft	0.62	646	H.soft	0.54	0.5	-1.73
43	H.soft	0.68	638	C.ar	-1.99	0.5	-1.44
43	H.soft	0.68	639	C.ar	-1.96	0.5	-1.46
37	H.soft	0.97	1426	H.soft	1.27	1.0	-1.26
48	H.soft	0.99	637	C.ar	-1.87	0.5	-1.41
62	H.soft	0.79	1092	H.soft	0.61	1.0	-3.08
47	H.soft	1.0	633	C.ar	-1.82	0.5	-1.43
47	H.soft	1.0	636	C.ar	-1.98	0.5	-1.35
52	H.soft	1.0	1512	H.soft	0.81	1.0	-2.3
30	N.ar	-2.92	1091	H.soft	0.34	0.5	-0.66
29	C.ar	-1.66	1091	H.soft	0.34	0.5	-1.67
60	H.soft	1.21	942	H.soft	0.34	1.0	-2.54
26	C.ar	-1.02	1199	H.soft	0.33	0.5	-1.97
50	H.O	3.62	1268	O.3.alcohol	-5.37	1.0	-0.69
54	H.N	2.82	1223	O.2.am	-7.73	1.0	0.08
14	O.3.alcohol	-3.79	1427	H.N	3.44	1.0	0.59

Table S97: 4W9I protein-ligand complex

Ligand atom number	Ligand atom type	Ligand AIP value	Protein atom number	Protein atom type	Protein AIP value	f	$\Delta\Delta G$ (kJ/mol)
26	C.ar	-0.98	858	H.soft	0.69	0.5	-1.87
27	C.ar	-0.63	904	H.soft	0.34	0.5	-2.07
28	S.2.phene	-0.55	857	H.soft	0.5	0.5	-2.03
28	S.2.phene	-0.55	904	H.soft	0.34	0.5	-2.09
21	C.ar	-1.32	859	H.soft	0.85	0.5	-1.7
17	C.2	-1.36	859	H.soft	0.85	0.5	-1.68
29	C.ar	-1.67	898	O.2.am	-8.69	0.5	8.57
30	N.ar	-2.95	907	H.soft	0.48	0.5	-0.68
57	H.soft	1.57	898	O.2.am	-1.8	0.5	-1.02
45	H.soft	1.29	1439	C.ar	-1.87	0.5	-1.24
45	H.soft	1.29	595	C.ar	-1.57	0.5	-1.33
28	S.2.phene	-0.16	1133	H.soft	0.33	0.5	-2.12
28	S.2.phene	-0.16	415	C.ar	-1.53	0.5	-1.65
42	H.soft	0.97	603	C.ar	-1.96	0.5	-1.38
44	H.soft	1.02	601	C.ar	-1.87	0.5	-1.4
44	H.soft	1.02	599	C.ar	-1.67	0.5	-1.48
24	C.ar	-0.74	1126	H.soft	0.22	0.5	-2.08
25	C.ar	-0.76	1126	H.soft	0.22	0.5	-2.07
28	S.2.phene	0.0	539	H.soft	0.34	0.5	-2.14
43	H.soft	1.06	597	C.ar	-1.82	0.5	-1.4
43	H.soft	1.06	600	C.ar	-1.98	0.5	-1.33
47	H.soft	1.43	853	C.ar	-1.32	0.5	-1.24
47	H.soft	1.43	852	C.ar	-1.53	0.5	-1.21
41	H.soft	1.33	602	C.ar	-1.99	0.5	-1.17
58	H.soft	0.81	1027	H.soft	0.61	1.0	-3.05
29	C.ar	-1.64	971	H.soft	0.33	0.5	-1.68
48	H.soft	1.06	1447	H.soft	0.81	1.0	-2.16
26	C.ar	-0.64	1134	H.soft	0.33	0.5	-2.08
46	H.O	3.7	1203	O.3.alcohol	-5.37	1.0	-0.75
50	H.N	2.88	1158	O.2.am	-7.73	1.0	-0.12
14	O.3.alcohol	-3.82	1362	H.N	3.44	1.0	0.57

Table S98: 5C28 protein-ligand complex

Ligand atom number	Ligand atom type	Ligand AIP value	Protein atom number	Protein atom type	Protein AIP value	f	$\Delta\Delta G$ (kJ/mol)
0	C.ar	-1.17	1027	C.ar	-1.56	0.5	-1.11
1	C.ar	-1.7	1023	C.ar	-1.52	0.5	-0.74
5	C.ar	-1.58	1026	C.ar	-1.56	0.5	-0.8
6	N.ar	-1.08	1025	C.ar	-1.55	0.5	-1.18
7	C.ar	-0.34	367	H.soft	0.33	0.5	-2.11
0	C.ar	-0.52	606	H.soft	0.34	0.5	-2.09
1	C.ar	-1.32	610	H.soft	0.33	0.5	-1.85
2	N.ar	-1.04	610	H.soft	0.33	0.5	-1.97
7	C.ar	-0.13	606	H.soft	0.34	0.5	-2.12
2	N.ar	-1.2	1024	C.ar	-1.53	0.5	-1.11
20	H.soft	0.78	1022	C.ar	-1.44	0.5	-1.69
20	H.soft	0.78	928	N.pl3.am	-0.26	0.5	-1.88
14	H.soft	0.58	640	C.ar	-1.44	0.5	-1.75
14	H.soft	0.58	928	N.pl3.am	-0.26	0.5	-2.01
6	N.ar	-0.14	699	H.soft	0.71	0.5	-1.92
13	H.soft	0.77	641	C.ar	-1.53	0.5	-1.66
2	N.ar	-6.27	934	H.N	2.7	1.0	0.24

References

(1) Storer, M. C.; Zator, K. J.; Reynolds, D. P.; Hunter, C. A. An atomic surface site interaction point description of non-covalent interactions. *Chemical Science* **2024**, *24*, 160–170.

(2) Su, M.; Yang, Q.; Du, Y.; Feng, G.; Liu, Z.; Li, Y.; Wang, R. Comparative Assessment of Scoring Functions: The CASF-2016 Update. *Journal of Chemical Information and Modeling* **2019**, *59*, 895–913.

(3) O'Boyle, N. M.; Banck, M.; James, C. A.; Morley, C.; Vandermeersch, T.; Hutchison, G. R. Open Babel: An open chemical toolbox. *Journal of Cheminformatics* **2011**, *3*.

(4) NWChem: A comprehensive and scalable open-source solution for large scale molecular simulations. *Computer Physics Communications* **2010**, 1477–1489.

(5) Storer, M. C. Remodelling Surface Site Interaction Points. Ph.D. thesis, University of Cambridge, 2023.

(6) Zator, K. J.; Hunter, C. A. Prediction of solution phase association constants by mapping contact points in intermolecular complexes. *Physical Chemistry Chemical Physics* **2025**, *27*, 11343–11352.
